# Tandem Three-Component Reactions of Aldehyde, Alkyl Acrylate, and Dialkylmalonate Catalyzed by Ethyl Diphenylphosphine

**DOI:** 10.3390/molecules17032529

**Published:** 2012-03-02

**Authors:** Yeong-Jiunn Jang, Siang-En Syu, Yi-Wun Jhang, Yu-Ting Lee, Chia-Jui Lee, Ko-Wei Chen, Utpal Das, Wenwei Lin

**Affiliations:** Department of Chemistry, National Taiwan Normal University No. 88, Section 4, Tingchow Road, Taipei 11677, Taiwan

**Keywords:** multicomponent reaction, Morita-Baylis-Hillman, chemoselectivity, Michael addition, aldehyde

## Abstract

A new highly efficient three-component reaction of alkyl acrylate, aldehyde and dialkyl malonate using ethyl diphenylphosphine as organocatalyst has been described. Various highly functional compounds bearing hydroxyl groups and the ester functions can be easily prepared in moderate to good yields according to our one-step procedure. The reactions are believed to proceed via Morita-Baylis-Hillman reactions of alkyl acrylate and aldehydes, followed by the Michael addition reactions of dialkyl malonates. Our reactions indicated that the intermediate species formed in the phosphine-catalyzed MBH reaction are an effective organic base to catalyze the Michael addition reactions of dialkyl malonates to the preformed MBH adducts.

## 1. Introduction

Carbon-carbon bond formation is the most important organic reaction because it plays a fundamental role in making carbon-frameworks of organic compounds for numerous interesting studies concerning reactivity, chemoselectivity, and stereoselectivity [1,2]. Multicomponent reactions have long been recognized to play a key role in the development of synthetic methodologies because of their possible generation of an adduct in a single step from three or more reactants usually accompanied by bond-forming efficiency and atom economy [3,4,5,6,7,8,9]. Successful application of multicomponent reaction highly relies on the good chemoselectivity in the presence of all the reactants [10,11,12].

The Baylis-Hillman reaction adduct, resulting from alkyl acrylate and aldehyde is a good Michael acceptor because of the activated ester functionality by the neighboring hydroxy group [13,14,15,16,17,18,19,20]. Successful applications of the Baylis-Hillman adducts for further transformation by Michael additions of nucleophiles had been reported [21,22,23,24]. However, the Baylis-Hillman reactions are well known for their slow reaction rates and moderate to high yields, and therefore the whole processes often take several days to complete the following Michael reactions [4]. Further effort to simplify the whole process was undertaken by one-pot sequential Baylis-Hillman and Michael reactions with aldehyde, methyl acrylate, and nitroalkane in the presence of DBU (1.0 equiv.) with 26–62% yields [21], albeit the one-step three-component reaction failed due to the dominant Michael addition of DBU-deprotonated nitroalkane toward methyl acrylate. Therefore, a strong demand remains for an efficient approach.

**Scheme 1:**
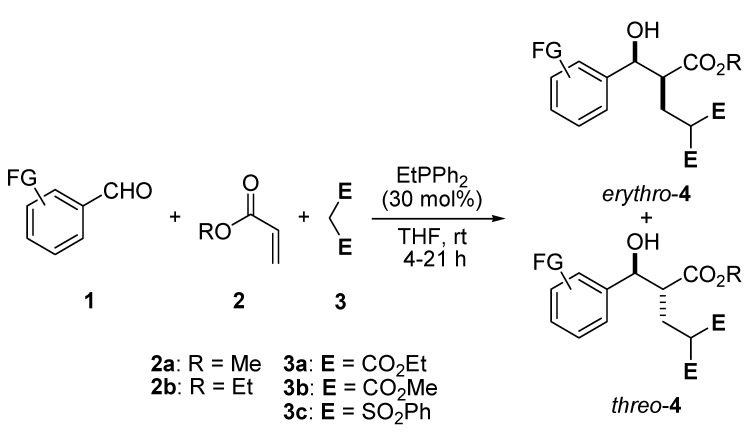
A three-component reaction of aromatic aldehyde **1**, alkyl acrylate **2**, and functional alkane **3** catalysed by EtPPh_2_.

In continuation of our efforts to simplify the whole process, we envisaged that instead of using a tertiary amine as an organocatalyst, it should be possible to carry out a phosphine-catalyzed three-component reaction starting from the Baylis-Hillman reaction of aldehyde **1** and alkyl acrylate **2**, which is followed by the Michael addition of dialkylmalonate **3** to the resulting adduct [25,26,27,28]. Herein, dialkylmalonates **3** become activated by the action of the intermediate species formed in the phosphine-catalyzed MBH reaction, because phosphines were much poorer bases than amines though the former are stronger nucleophiles [29]. In particular, phosphines are known catalysts capable of promoting Michael reactions in the absence of added bases [30,31,32,33,34]. However, to the best of our knowledge, there is no report of a successful reaction or related study that utilizes **3** as the reacting partner. Therefore, we wish to report a highly efficient three-component reaction of **1**, **2**, and **3** catalyzed by ethyl diphenylphosphine (Scheme 1).

## 2. Results and Discussion

4-Nitrobenzaldehyde (**1a**), methyl acrylate (**2a**, 2 equiv.) and diethyl malonate (**3a**, 1.2 equiv.) were initially chosen and reacted in *t*-BuOH in the presence of EtPPh_2_ at room temperature, providing the densely functionalized three component adduct **4a** in moderate yield (47%) and good chemoselectivity (21:79 dr) within 0.5 h (Table 1, entry 1).

**Table 1:** Optimization of reaction conditions for a three-component reaction of 4-nitrobenzaldehyde(**1a**), methyl acrylate (**2a**) and diethyl malonate (**3a**) catalyzed by different organocatalysts ^a^. 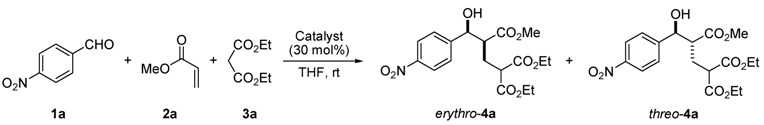

Entry	CH_2_(CO_2_Et)_2_ (3a) (equiv.)	Catalyst	Solvent	Time (h)	dr of 4a ^b^	Yield of 4a (%) ^c^
1	1.2	EtPPh_2_	*t*-BuOH	0.5	21:79	47
2	1.2	PPh_3_	*t*-BuOH	24	-	Trace
3	1.2	PBu_3_	*t*-BuOH	4	-	Trace ^d^
4	1.2	**DABCO**	*t*-BuOH	24	33:67	25
5	1.2	EtPPh_2_	*i*-PrOH	2.5	25:75	38
6	1.2	EtPPh_2_	CH_2_Cl_2_	3	12:88	38
7	1.2	EtPPh_2_	Toluene	4	15:85	51
8	1.2	EtPPh_2_	THF	4	18:82	60
9	1.2	EtPPh_2_^e^	THF	2	12:88	49
10	1.2	EtPPh_2_^f^	THF	24	21:79	62
11	1.5	EtPPh_2_	THF	5	16:84	54
12 ^g^	1.2	EtPPh_2_	THF/*t*-BuOH ^h^	1.5	19:81	43
13	1.2	**DMAP**	THF	24	-	Trace
14	1.2	**DBU**	THF	24	-	Trace

^a^ Unless stated otherwise, the reaction was performed using 4-nitrobenzaldehyde (**1a**) (0.5 mmol) and methyl acrylate (**2a**) (1.0 mmol) with 30 mol% catalyst in solvent (0.5 mL) at room temperature; ^b^ The diastereometric ratio of **4a** was determined by crude ^1^H-NMR analysis; ^c^ Yield of analytically pure isolated product; ^d^ Only significant amount of 4-nitrobenzaldehyde (**1a**) and diethyl malonate (**3a**) were observed even after 8 h; ^e^ The reaction was performed with 50 mol% EtPPh_2_; ^f^ The reaction was performed with 20 mol% EtPPh_2_; ^g^ The reaction was performed at 10 °C; ^h^ THF/*t*-BuOH = 1/4.

Trace amounts of the product **4a** were obtained when the less reactive PPh_3_ was used (entry 2). Significant amounts of 4-nitrobenzaldehyde (**1a**) and diethyl malonate (**3a**) were recovered, because the acrylate was prone to undergo polymerization when the extremely active PBu_3_ was used as the catalyst (entry 3). DABCO, which has weaker nucleophilicity than that of EtPPh_2_, catalyzed the three component reaction of **1a**, **2a** and **3a** and afforded **4a** in only 25% yield within 24 h (entry 4). Inferior results were obtained when the reactions were carried out in *i*-PrOH, CH_2_Cl_2_ and toluene (entries 5–7). Interestingly, when a polar aprotic solvent was used, such as THF, a significantly increased yield was observed (**4a**, 60% yield, entry 8). Increasing the amount of EtPPh_2_, prolonging the reaction time with 1.5 equiv. of **3a**, or using a co-solvent system (THF/*t*-BuOH = 1/4) did not improve the final results either (entries 9, 11 and 12), and furthermore a worse diastereometric excess resulted from decreasing the amount of EtPPh_2_ (entry 10). DMAP and DBU were also examined, but only trace amounts of adduct **4a** could be observed (entries 13 and 14).

The broad reaction scope of our protocol was demonstrated by further studies disclosed in Table 2. It showed that chemoselective three-component reactions of various aromatic aldehydes **1a**–**f**, alkyl acrylate **2** (2 equiv.), and **3a** (1.2 equiv.) in the presence of EtPPh_2_ (30 mol%) took place in 4–21 h, leading to the corresponding adducts **4a**–**j** in 36–63% yields.

**Table 2:** A three component reaction of aromatic aldehyde **1**, alkyl acrylate **2**, and dialkyl malonate **3** catalysed by EtPPh_2_^a^. 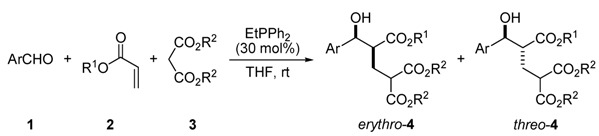

Entry	Ar	R^1^	R^2^	Time (h)	dr of 4 ^b^	Yield of 4 (%) ^c^
1	4-NO_2_C_6_H_4_ (**1a**)	CH_3_ (**2a**)	C_2_H_5_ (**3a**)	4	18:82	**4a** (60)
2	3-NO_2_C_6_H_4 _(**1b**)	**2a**	**3a**	4	13:87	**4b** (63)
3	2-NO_2_C_6_H_4 _(**1c**)	**2a**	**3a**	4	37:63	**4c** (36)
4	4-CF_3_C_6_H_4 _(**1d**)	**2a**	**3a**	10	20:80	**4d** (57)
5	4-CNC_6_H_4 _(**1e**)	**2a**	**3a**	4	17:83	**4e** (50)
6	3-Pyridyl (**1f**)	**2a**	**3a**	21	18:82	**4f** (55)
7	4-NO_2_C_6_H_4 _(**1a**)	C_2_H_5_ (**2b**)	**3a**	4	9:91	**4g** (50)
8	3-NO_2_C_6_H_4 _(**1b**)	**2b**	**3a**	4	15:85	**4h** (53)
9	4-CNC_6_H_4 _(**1e**)	**2b**	**3a**	6	11:89	**4i** (51)
10	3-Pyridyl (**1f**)	**2b**	**3a**	20	14:86	**4j** (52)
11	4-NO_2_C_6_H_4_ (**1a**)	CH_3_ (**2a**)	CH_3_ (**3b**)	5	13:87	**4k** (39)

^a^ Unless stated otherwise, the reaction was performed using **1** (0.5 mmol), **2** (2.0 equiv.), and **3a** (1.2 equiv.) in the presence of EtPPh_2_ (30 mol%) in THF (0.5 mL) at room temperature; ^b^ The diastereomeric ratio of **4** was determined by ^1^H-NMR analysis. The stereochemistry of **4a**–**b**, **4d**, **4e**, and **4g**–**i** was determined by ^1^H-NMR analysis in comparison to **5**. For **4c**, **4f**, and **4j**, their stereochemistry is not determined; ^c^ Yield of analytically pure isolated product.

**Scheme 2:**
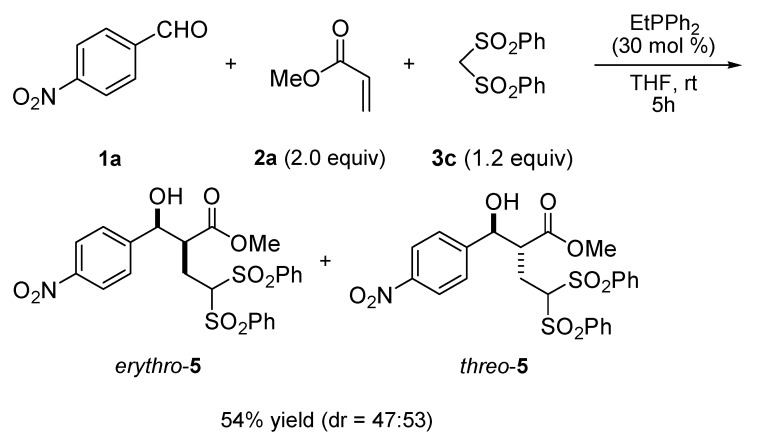
A three-component reaction of 4-nitrobenzaldehyde (**1a**), methyl acrylate (**2a**, 2 equiv.) and bis(phenylsulfonyl)methane (**3c**, 1.2 equiv.) catalyzed by EtPPh_2_.

A steric effect was found in case of reactions with an *ortho*-substituted aromatic aldehydes. For example, aromatic aldehydes bearing a nitro group in the *para*- or *meta*-position, like **1a** or **1b**, reacted with **2a** and **3a** within 4 h to provide the corresponding adducts **4a** or **4b** with good stereoselectivities (18:82 dr, 13:87 dr) in 60% or 63% yield, respectively (entries 1 and 2). However, in the case of 2-nitrobenzaldehyde (**1c**), poor results for the formation of **4c** were obtained (36% yield and 37:63 dr, entry 3). Other substituted aromatic aldehydes, such as 4-trifluoromethylbenzaldehyde (**1d**) or 4-cyanobenzaldehyde (**1e**), reacted like **2a** and **3a**, generating **4d** or **4e** in 57% or 50% yield with 20:80 dr or 17:83 dr, resepectively (entries 4 and 5). Moderate yield and good stereoselectivity of **4f** was also produced when heteroaromatic aldehyde **1f** was employed in our protocol (entry 6). The reaction of **1a**, **1b**, **1e**, or **1f** and **3a** also proceeded well when ethyl acrylate (**2b**) was used as the activated olefin, furnishing the corresponding adducts **4g**, **4h**, **4i**, or **4j** in moderate yield with high stereoselectivity (entries 7–10). In addition to diethyl malonate (**3a**), dimethyl malonate (**3b**) and bis(phenylsulfonyl)methane (**3c**) were also investigated as carbon nucleophiles for this protocol. Both of them worked smoothly with 4-nitrobenzaldehyde (**1a**) and methyl acrylate (**2a**, 2 equiv.) in the presence of EtPPh_2_ to give the corresponding adducts **4k** (39% yield, 13:87 dr) and **5** (54% yield, 47:53 dr) (entry 11 and Scheme 2) [35].

**Scheme 3:**
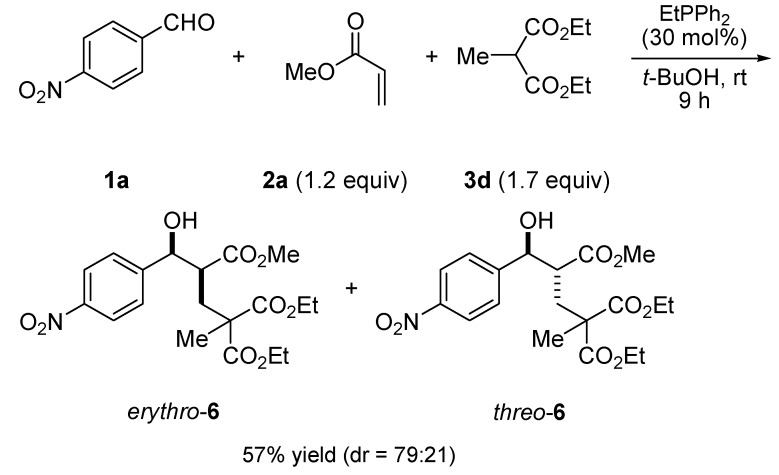
A three-component reaction of 4-nitrobenzaldehyde (**1a**), methyl acrylate (**2a**, 1.2equiv.), and diethyl methylmalonate (**3d**, 1.7 equiv.) catalyzed by EtPPh_2_.

Remarkably, a tandem three-component reaction of 4-nitrobenzaldehyde (**1a**), methyl acrylate (**2a**, 1.2 equiv.) and a sterically bulky carbon nucleophile, diethyl methylmalonate (**3d**) (1.7 equiv.), ocurred successfully at room temperature within 9 h to provide the desired product **6n** in 57% yield with good stereoselectivity (Scheme 3).

## 3. Experimental

### 3.1. General

All reactions were carried out under a nitrogen atmosphere in dried glassware. All starting materials were purchased from commercial sources and used without further purification. THF was continuously refluxed and freshly distilled from sodium benzophenone ketyl under nitrogen. *t*-BuOH was dried and degassed before use. Yields refer to isolated yields of compounds estimated to be >95% pure as determined by ^1^H-NMR in a AV-400 or AV-500 Bruker using CDCl_3_ as solvent at 400 Hz, respectively. MS and HRMS were recorded in a Finnigan TSQ 700 and JEOL JMS-700 mass spectrometers. Analytical thin layer chromatography (TLC) was performed using Merck 60 F254 precoated silica gel plate (0.2 mm thickness). Flash chromatography was performed using Merck silica gel 60.

### 3.2. Typical Synthetic Procedure

#### 3.2.1. Optimization of Reaction Conditions for an Organocatalytic Three-Component Reaction of 4-Nitrobenzaldehyde (**1a**), Methyl Acrylate (**2a**), and Diethyl Malonate (**3a**) (TP for Table 1)

A dry and nitrogen-flushed 10-mL Schlenk flask, equipped with a magnetic stirring bar and a septum, was charged with a solution of **1a** (75.6 mg, 0.5 mmol) and **3a** (91.0 μL, 1.2 equiv.) in solvent (dry degassed *t*-BuOH, *i*-PrOH or dry THF) (0.5 mL). Methyl acrylate (**2a**) (90.0 μL, 2.0 equiv.) and catalyst (EtPPh_2_, PPh_3_, PBu_3_, DABCO, DMAP or DBU) (30 mol%) were added, and the reaction mixture was stirred for indicated time at room temperature. Thereafter, the solvent was removed by evaporation in vacuo. Purification by flash chromatography (*n*-hexanes/ethyl acetate 4:1) furnished the adduct **4a**.

#### 3.2.2. Typical Procedure for a Three-Component Reaction of Aromatic Aldehyde, Alkyl Acrylate, and Diethyl Malonate Catalyzed by EtPPh_2_ (TP for Table 2)

A dry and nitrogen-flushed 10-mL Schlenk flask, equipped with a magnetic stirring bar and a septum, was charged with a solution of 1 (0.5 mmol) and **3a** or **3b** (1.2 equiv.) in dry degassed THF (0.5 mL). Alkyl acrylate 2a or 2b (2.0 equiv.) and EtPPh_2_ (30.7 μL, 30 mol%) were added, and the reaction mixture was stirred for 4–21 h at room temperature. Thereafter, the solvent was removed by evaporation in vacuo. Purification by flash chromatography furnished the adducts *erythro*-**4** and *threo*-**4**.

#### 3.2.3. Procedure for Preparation of **5** (TP for Scheme 2)

A dry and nitrogen-flushed 10-mL Schlenk flask, equipped with a magnetic stirring bar and a septum, was charged with a solution of **1a** (151.1 mg, 1.0 mmol) and **3c** (355.6 mg, 1.2 equiv.) in dry THF (1.0 mL). Methyl acrylate **2a** (180.0 μL, 2.0 equiv.) and EtPPh_2_ (61.0 μL, 30 mol%) were added, and the reaction mixture was stirred for 5 h at room temperature. Thereafter, the solvent was removed by evaporation in vacuo. Purification by flash chromatography furnished the adducts *erythro*-**5** and *threo*-**5** (dr = 47:53, 288.1 mg, 54%).

#### 3.2.4. Procedure for Preparation of **6** (TP for Scheme 3)

A dry and nitrogen-flushed 10-mL Schlenk flask, equipped with a magnetic stirring bar and a septum, was charged with a solution of **1a** (0.5 mmol) and **3d** (102.3 μL, 1.2 equiv.) in dry *t*-BuOH (0.5 mL). Methyl acrylate **2a** (90.0 μL, 2.0 equiv.) and EtPPh_2_ (30.7 μL, 30 mol%) were added, and the reaction mixture was stirred for 9 h at room temperature. Thereafter, the solvent was removed by evaporation in vacuo. Purification by flash chromatography furnished the adducts **6** (dr = 79:21, 117.1 mg, 57%).

*Erythro*-*1,1-diethyl 3-methyl 4-hydroxy-4-(4-nitrophenyl)butane-1,1,3-tricarboxylate* (**erythro-4a**). ^1^H-NMR (400 MHz, CDCl_3_, 25 °C) *δ*/ppm: 8.18 (d, 2H, * J* = 8.4 Hz), 7.52 (d, 2H, * J* = 8.4 Hz), 5.13 (d, 1H, *J* = 3.7 Hz), 4.22–4.01 (m, 4H), 3.63 (s, 3H), 3.44 (s, 1H), 3.40–3.27 (m, 1H), 2.89–2.76 (m, 1H), 2.36–2.20 (m, 1H), 2.17–2.01 (m, 1H), 1.27–1.08 (m, 6H); ^13^C-NMR (100 MHz, CDCl_3_, 25 °C) *δ*/ppm: 173.8, 168.8, 168.6, 148.3, 147.4, 127.0, 123.5, 72.9, 61.7, 61.5, 52.1, 49.8, 49.7, 25.3, 13.9; MS (70 eV, EI) *m/z* (%): 398 (2) [M+1]^+^, 380 (8), 319 (80), 302 (77), 274 (43), 245 (72), 160 (100), 133 (82), 127 (85), 113 (48), 105 (52), 55 (81); HRMS (MALDI) for C_18_H_23_NO_9_Na, [M+Na]^+^ (420.1270) found: 420.1275.

*Threo*-*1,1-diethyl 3-methyl 4-hydroxy-4-(4-nitrophenyl)butane-1,1,3-tricarboxylate* (**threo-4a**). ^1^H-NMR (400 MHz, CDCl_3_, 25 °C) *δ*/ppm: 8.22 (d, 2H, * J* = 8.8 Hz), 7.50 (d, 2H, * J* = 8.6 Hz), 4.98 (d, 1H, *J* = 5.8 Hz), 4.23–4.14 (m, 4H), 3.64 (s, 3H), 3.38 (dd, 1H *J* = 5.7 Hz, 9.3 Hz), 2.97–2.90 (m, 1H), 2.30–2.21 (m, 1H), 2.18–2.09 (m, 1H), 1.29–1.21 (m, 6H); ^13^C-NMR (100 MHz, CDCl_3_, 25 °C) *δ*/ppm: 173.8, 168.6, 168.5, 148.7, 147.6, 127.1, 123.7, 73.8, 61.8, 61.7, 52.1, 49.8, 49.6, 27.9, 14.0; MS (70 eV, EI) *m/z* (%): 398 (1) [M+1]^+^, 380 (5), 319 (85), 302 (79), 274 (40), 245 (77), 223 (86), 160 (100), 133 (93), 127 (85), 113 (51), 105 (49), 55 (81); HRMS (MALDI) for C_18_H_23_NO_9_Na, [M+Na]^+^ (420.1270) found: 420.1283.

*Erythro-1,1-diethyl 3-methyl 4-hydroxy-4-(3-nitrophenyl)butane-1,1,3-tricarboxylate* (**erythro-4b**). ^1^H-NMR (400 MHz, CDCl_3_, 25 °C) *δ*/ppm: 8.23 (s, 1H), 8.17–8.11 (m, 1H), 7.69 (d, 1H, *J* = 7.7 Hz), 7.52 (t, 1H *J* = 7.9 Hz), 5.14 (*pseudo* t, 1H, *J* = 4.1 Hz), 4.22–4.06 (m, 4H), 3.65 (s, 3H), 3.37 (dd, 1H *J* = 9.3 Hz, 5.3 Hz), 2.90–2.82 (m, 1H), 2.35–2.25 (m, 1H), 2.18–2.07 (m, 1H), 1.28–1.14 (m, 6H). ^13^C-NMR (100 MHz, CDCl_3_, 25 °C) *δ*/ppm: 173.9, 168.9, 168.7, 148.3, 143.2, 132.2, 129.3, 122.8, 121.2, 72.7, 61.7, 61.6, 52.2, 49.9, 49.8, 25.4, 13.9. MS (20 eV, EI) *m/z* (%): 398 (5) [M+1]^+^, 380 (10), 348 (5), 246 (41), 214 (11), 187 (11), 173 (7), 160 (100), 134 (20). HRMS (ESI) for C_18_H_23_NO_9_Na, [M+Na]^+^ (420.1270) found: 420.1268.

*Threo-1,1-diethyl 3-methyl 4-hydroxy-4-(3-nitrophenyl)butane-1,1,3-tricarboxylate* (**threo-4b**). ^1^H-NMR (400 MHz, CDCl_3_, 25 °C) *δ*/ppm: 8.16 (s, 1H), 8.15–8.08 (m, 1H), 7.65 (d, 1H, *J* = 7.7 Hz), 7.51 (t, 1H *J* = 7.9 Hz), 4.95 (*pseudo* s, 1H), 4.18–4.07 (m, 4H), 3.63 (s, 3H), 3.60 (brs, 1H), 3.33 (dd, 1H *J* = 9.4 Hz, 5.6 Hz), 2.95–2.85 (m, 1H), 2.24–2.12 (m, 1H), 2.08–1.98 (m, 1H), 1.25–1.15 (m, 6H). ^13^C-NMR (100 MHz, CDCl_3_, 25 °C) *δ*/ppm: 173.7, 168.6, 168.4, 148.2, 143.6, 132.3, 129.4, 122.9, 121.2, 73.7, 61.7, 61.6, 52.0, 50.0, 49.6, 27.7, 13.9, 13.7. MS (20 eV, EI) *m/z* (%):398 (1) [M+1]^+^, 380 (5), 348 (5), 301 (6), 246 (41), 214 (8), 187 (11), 173 (8), 160 (100), 134 (5). HRMS (ESI) for C_18_H_23_NO_9_Na, [M+Na]^+^ (420.1270) found: 420.1273.

*Erythro-1,1-diethyl 3-methyl 4-hydroxy-4-(2-nitrophenyl)butane-1,1,3-tricarboxylat* (**erythro-4c**). ^1^H-NMR (400 MHz, CDCl_3_, 25 °C) *δ*/ppm: 8.00 (d, 1H, * J* = 8.2 Hz), 7.89 (d, 1H, *J* = 7.8 Hz), 7.68 (t, 1H *J* = 7.6 Hz), 7.47 (t, 1H *J* = 7.8 Hz), 5.72 (*pseudo* s, 1H), 4.19–4.10 (m, 2H), 4.07 (quart, 2H *J* = 7.1 Hz), 3.69 (s, 3H), 3.43 (d, 1H, *J* = 3.0 Hz), 3.36 (dd, 1H, *J* = 9.8 Hz, *J* = 5.3 Hz), 2.98 (dt, 1H, *J* = 10.4 Hz, *J* = 3.8 Hz), 2.38–2.28 (m, 1H), 2.17–2.08 (m, 1H), 1.23 (t, 3H, *J* = 7.2 Hz), 1.15 (t, 3H, *J* = 7.1 Hz). ^13^C-NMR (100 MHz, CDCl_3_, 25 °C) *δ*/ppm: 174.6, 168.9, 168.6, 147.5, 136.1, 133.5, 129.3, 128.7, 124.9, 69.2, 61.6, 61.5, 52.2, 49.9, 47.6, 25.1, 13.9. MS (20 eV, EI) *m/z* (%): 398 (100) [M+1]^+^, 385 (62), 380 (61), 363 (75). HRMS (FAB) for C_18_H_24_NO_9_, [M+H]^+^ (398.1451) found: 398.1457.

*Threo-1,1-diethyl 3-methyl 4-hydroxy-4-(2-nitrophenyl)butane-1,1,3-tricarboxylate* (**threo-4c**). ^1^H-NMR (400 MHz, CDCl_3_, 25 °C) *δ*/ppm: 7.98 (dd, 1H,* J* = 8.0 Hz, *J* = 0.6 Hz), 7.71–7.61 (m, 2H), 7.49–7.42 (m, 1H), 5.46 (dd, 1H, *J* = 6.7 Hz, *J* = 5.2 Hz), 4.25–4.14 (m, 4H), 3.84 (d, 1H *J* = 7.5 Hz), 3.58 (s, 3H), 3.43 (dd, 1H, *J* = 8.9 Hz, *J* = 6.2 Hz), 3.11–3.04 (m, 1H), 2.45–2.34 (m, 1H), 2.30–2.20 (m, 1H), 1.30–1.22 (m, 6H). ^13^C-NMR (100 MHz, CDCl_3_, 25 °C) *δ*/ppm: 174.3, 168.9, 168.8, 148.1, 137.4, 133.7, 129.0, 128.6, 125.1, 70.2, 61.9, 52.1, 49.9, 48.9, 28.8, 14.1. MS (FAB) *m/z* (%): 398 (100) [M+1]^+^, 385 (60), 380 (65), 363 (72). HRMS (FAB) for C_18_H_24_NO_9_, [M+H]^+^ (398.1451) found: 398.1454.

*Erythro-1,1-diethyl 3-methyl 4-hydroxy-4-(4-(trifluoromethyl)phenyl)butane-1,1,3-tricarboxylate* (**erythro-4d**). ^1^H-NMR (400 MHz, CDCl_3_, 25 °C) *δ*/ppm: 7.60 (d, 2H,* J* = 8.2 Hz), 7.47 (d, 2H, * J* = 8.1 Hz), 5.10 (pseudo t, 1H, *J* = 3.9 Hz), 4.19–4.03 (m, 4H), 3.64 (s, 3H), 3.36 (dd, 1H, *J* = 5.4 Hz, 9.5 Hz), 3.17 (d, *J* = 3.4 Hz), 2.88–2.79 (m, 1H), 2.35–2.24 (m, 1H), 2.19–2.09 (m, 1H), 1.22 (t, 3H, *J *= 7.2 Hz), 1.17 (t, 3H, *J *= 7.2 Hz). ^13^C-NMR (100 MHz, CDCl_3_, 25 °C). *δ*/ppm: 174.1, 168.9, 168.7, 144.9, 130.0 (quartet,*J *= 32 Hz), 126.7, 125.2 (quartet,*J *= 4 Hz), 123.9 (quartet,*J *= 270 Hz), 130.0 (quartet,*J *= 33 Hz), 126.4, 125.3 (quartet,*J *= 3 Hz), 123.8 (quartet,*J *= 269 Hz), 73.1, 61.6, 61.5, 52.1, 49.9, 49.8, 25.4, 14.0, 13.9. MS (FAB) *m/z* (%): 420 (1) [M]^+^, 403 (10), 360 (58), 245 (42), 173 (31), 160 (100), 126 (38), 55 (20). HRMS (ESI) for C_19_H_23_F_3_NaO_7_, [M+Na]^+^ (443.1294) found: 400.1290.

*Threo-1,1-diethyl 3-methyl 4-hydroxy-4-(4-(trifluoromethyl)phenyl)butane-1,1,3-tricarboxylate* (**threo-4d**). ^1^H-NMR (400 MHz, CDCl_3_, 25 °C) *δ*/ppm: 7.57 (d, 2H,* J* = 8.2 Hz), 7.40 (d, 2H,* J* = 8.1 Hz), 4.85 (d, 1H, *J* = 6.4 Hz), 4.15–4.07 (m, 4H), 3.61 (s, 4H), 3.30 (dd, 1H, *J* = 5.5 Hz, 9.6 Hz), 2.90–2.81 (m, 1H), 2.19–2.08 (m, 1H), 2.01–1.90 (m, 1H), 1.24–1.13 (m, 6H). ^13^C-NMR (100 MHz, CDCl_3_, 25 °C) *δ*/ppm: 73.8, 168.5, 168.4, 145.3, 130.0 (quartet, *J *= 32 Hz), 126.7, 125.2 (quartet, *J *= 4 Hz), 123.9 (quartet, *J *= 270 Hz), 74.2, 61.5, 61.4, 52.7, 50.2, 49.6, 27.5, 13.6. MS (20 eV, EI) *m/z* (%): 420 (3) [M]^+^, 403 (14), 360 (69), 245 (34), 173 (27), 160 (100), 126 (42), 55 (20). HRMS (ESI) for C_19_H_23_F_3_NaO_7_, [M+Na]^+^ (443.1294) found: 400.1288.

*Erythro-1,1-diethyl 3-methyl 4-(4-cyanophenyl)-4-hydroxybutane-1,1,3-tricarboxylate* (**erythro-4e**). ^1^H-NMR (400 MHz, CDCl_3_, 25 °C) *δ*/ppm: 7.65 (d, 2H, * J* = 8.4 Hz), 7.48 (d, 2H, * J* = 8.2 Hz), 5.10 (*pseudo* t, 1H, *J* = 3.9 Hz), 4.20–4.07 (m, 4H), 3.66 (s, 3H), 3.36 (dd, 1H, *J* = 5.4 Hz, 9.6 Hz), 3.20 (d, *J* = 3.4 Hz), 2.88–2.80 (m, 1H), 2.34–2.24 (m, 1H), 2.15–2.05 (m, 1H), 1.28–1.16 (m, 6H). ^13^C-NMR (100 MHz, CDCl_3_, 25 °C) *δ*/ppm: 174.0, 168.9, 168.7, 146.2, 132.2, 126.7, 118.6, 111.7, 73.0, 61.7, 61.6, 52.2, 49.8, 49.7, 25.3, 14.0. HRMS (MALDI) for C_19_H_23_NNaO_7_, [M+Na]^+^ (400.1372) found: 400.1385.

*Threo-1,1-diethyl 3-methyl 4-(4-cyanophenyl)-4-hydroxybutane-1,1,3-tricarboxylate* (**threo-4e**). ^1^H-NMR (400 MHz, CDCl_3_, 25 °C) *δ*/ppm: 7.61 (d, 2H, * J* = 8.3 Hz), 7.41 (d, 2H, * J* = 8.2 Hz), 4.87 (d, 1H, *J* = 6.2 Hz), 4.18–4.07 (m, 4H), 3.60 (s, 3H), 3.55 (brs, 1H), 3.30 (dd, 1H, *J* = 5.6 Hz, 9.5 Hz), 2.89–2.80 (m, 1H), 2.20–2.10 (m, 1H), 2.03–1.91 (m, 1H), 1.25–1.16 (m, 6H). ^13^C-NMR (100 MHz, CDCl_3_, 25 °C) *δ*/ppm: 173.7, 168.5, 168.4, 146.7, 132.2, 127.0, 118.5, 111.7, 74.0, 61.7, 61.6, 52.0, 49.9, 49.5, 27.7, 13.9. MS (20 eV, EI) *m/z* (%):78 (14) [M+1]^+^, 360 (13), 317 (39), 300 (29), 282 (16), 246 (21), 203 (31), 186 (29), 160 (100), 133 (15). HRMS (MALDI) for C_19_H_23_NNaO_7_, [M+Na]^+^ (400.1372) found: 400.1385.

*1,1-Diethyl 3-methyl 4-hydroxy-4-(pyridin-3-yl)butane-1,1,3-tricarboxylate* (**4f**). ^1^H-NMR (400 MHz, CDCl_3_, 25 °C) *δ*/ppm: 8.44 (s, 2H), 7.71–7.61 (m, 2H), 7.50–7.40 (m, 1H), 7.27–7.22 (m, 1H), 4.99 and 4.85 (d, 1H, *J* = 5.9&7.2 Hz), 4.42 (brs, 1H), 4.19–4.04 (m, 4H), 3.65 and 3.55 (s, 3H), 3.44–3.22 (m, 1H), 2.95–2.74 (m, 1H), 2.36–1.86 (m, 1H), 1.29–1.12 (m, 6H). ^13^C-NMR (100 MHz, CDCl_3_, 25 °C) *δ*/ppm: 173.8, 173.5, 168.9, 168.7, 168.6, 168.4, 149.2, 148.1, 137.1, 134.2, 134.1, 131.6, 130.8, 130.7, 128.6, 128.5, 123.5, 72.7, 71.6, 61.6, 61.5, 61.4, 51.9, 51.8, 50.4, 50.2, 49.9, 49.7, 27.7, 26.0, 13.9.

*Threo-triethyl 4-hydroxy-4-(4-nitrophenyl)butane-1,1,3-tricarboxylate* (**threo-4g**). ^1^H-NMR (400 MHz, CDCl_3_, 25 °C) *δ*/ppm: 8.16 (d, 2H, * J* = 8.7 Hz), 7.48 (d, 2H, * J* = 8.7 Hz), 4.93 (t, 1H, *J* = 6.2 Hz), 4.20–4.09 (m, 4H), 4.09–4.01 (m, 2H), 3.68 (d, 1H, *J* = 6.7 Hz), 3.33 (dd, 1H, *J* = 5.5 Hz, 9.6 Hz), 2.90–2.81 (m, 1H), 2.24–2.13 (m, 1H), 2.09–2.00 (m, 1H), 1.21 (t, 3H, *J *= 7.2), 1.12 (t, 3H, *J* = 7.2). ^13^C-NMR (100 MHz, CDCl_3_, 25 °C) *δ*/ppm: 173.3, 168.6, 168.4, 148.8, 147.4, 127.1, 123.5, 73.6, 61.7, 61.6, 61.2, 49.8, 49.5, 27.9, 13.9, 13.8. MS (MALDI) *m/z* (%): 434 (100) [M+Na]^+^, 368 (55), 352 (63). HRMS (MALDI) for C_19_H_25_NNaO_9_, [M+Na]^+^ (434.1427) found: 434.1436.

*Threo-triethyl 4-hydroxy-4-(3-nitrophenyl)butane-1,1,3-tricarboxylate* (**threo-4h**). ^1^H-NMR (400 MHz, CDCl_3_, 25 °C) *δ*/ppm: 8.18, (s, 1H), 8.15–8.09 (m, 1H), 7.66 (d, 1H, * J* = 7.7 Hz), 7.52, (d, 1H, * J* = 7.9 Hz), 4.96 (t, 1H, *J* = 5.2 Hz), 4.21–4.12 (m, 4H), 4.09 (quartet, 2H, *J* = 7.2 Hz), 3.60 (d, 1H, *J* = 6.2 Hz), 3.37 (dd, 1H, *J* = 5.5 Hz, 9.5 Hz), 2.95–2.84 (m, 1H), 2.28–2.18 (m, 1H), 2.14–2.03 (m, 1H), 1.28–1.19 (m, 6H), 1.14 (t, 3H, *J* = 7.2 Hz). ^13^C-NMR (100 MHz, CDCl_3_, 25 °C) *δ*/ppm: 173.3, 168.6, 168.5, 148.3, 143.7, 132.3, 129.4, 122.9, 121.2, 73.7, 61.7, 61.6, 61.2, 49.8, 49.6, 27.9, 13.9. MS (70 eV, EI) *m/z* (%):412 (4) [M+1]^+^, 393 (5), 320 (14), 302 (15), 260 (25), 185 (39), 160 (100), 133 (15), 55 (16). HRMS (MALDI) for C_19_H_25_NNaO_9_, [M+Na]^+^ (434.1427) found: 434.1431.

*Threo-triethyl 4-(4-cyanophenyl)-4-hydroxybutane-1,1,3-tricarboxylate* (**threo-4i**). ^1^H-NMR (400 MHz, CDCl_3_, 25 °C) *δ*/ppm: 7.65 (d, 2H, * J* = 8.2 Hz), 7.45 (d, 2H, * J* = 8.2 Hz), 4.91 (t, 1H, *J* = 6.4 Hz), 4.25–4.14 (m, 4H), 4.09 (quartet, *J* = 7.0 Hz), 3.38 (dd, 1H, *J* = 5.7 Hz, 9.4 Hz), 3.34 (d, 1H, *J* = 7.2 Hz), 2.93–2.83 (m, 1H), 2.31–2.20 (m, 1H), 2.19–2.07 (m, 1H), 1.26 (t, 6H, *J* = 7.2 Hz), 1.15 (t, 3H, *J* = 7.1 Hz). ^13^C-NMR (100 MHz, CDCl_3_, 25 °C) *δ*/ppm: 173.1, 168.5, 168.3, 146.8, 132.0, 126.9, 118.4, 111.5, 73.9, 61.5, 61.4, 61.0, 49.8, 49.4, 27.7, 13.8. MS (20 eV, EI) *m/z* (%): 391 (4) [M]^+^, 218 (19), 160 (100), 132 (10), 102 (30). HRMS (MALDI) for C_20_H_25_NNaO_7_, [M+Na]^+^ (414.1529) found: 414.1535.

*Threo-ethyl 3-(4-cyanophenyl)-2-((N,4-dimethylphenylsulfonamido)methyl)-3-hydroxypropanoate* (**threo-4j**). ^1^H-NMR (400 MHz, CDCl_3_, 25 °C) *δ*/ppm: 8.35 (s, 2H), 7.65 (d, 1H, * J* = 7.9 Hz), 7.22 (dd, 1H, * J* = 4.8 Hz, *J* = 7.7 Hz), 4.81 (d, 1H, *J* = 7.2 Hz), 4.16–3.96 (m, 6H), 3.26 (dd, 1H *J* = 6.2 Hz, 10.1 Hz), 2.85–2.74 (m, 1H), 2.14–2.01 (m, 1H), 1.94–1.82 (m, 1H), 1.22–1.09 (m, 9H). ^13^C-NMR (100 MHz, CDCl_3_, 25 °C) *δ*/ppm: 173.2, 168.5, 168.4, 148.7, 147.8, 137.3, 134.1, 123.5, 72.5, 61.5, 61.4, 60.9, 50.2, 49.6, 27.7, 13.9, 13.8, 13.7. MS (MALDI) *m/z* (%): 368 (100) [M+1]^+^, 350 (38), 305 (50), 289 (37). HRMS (MALDI) for C_18_H_26_NO_7_, [M+H]^+^ (368.1709) found: 368.1719.

*Threo-trimethyl 4-hydroxy-4-(4-nitrophenyl)butane-1,1,3-tricarboxylate* (**threo-4k**). ^1^H-NMR (400 MHz, CDCl_3_, 25 °C) *δ*/ppm: 8.20 (d, 2H, * J* = 8.7 Hz), 7.50 (d, 2H, * J* = 8.7 Hz), 4.96 (d, 1H, *J* = 6.0 Hz), 3.71 (s, 6H), 3.65 (s, 3H), 3.41 (dd, 1H, *J* = 5.7 Hz, 9.3 Hz), 2.92–2.88 (m, 1H), 2.24–2.19 (m, 1H), 2.13–2.08 (m, 1H). ^13^C-NMR (100 MHz, CDCl_3_, 25 °C) *δ*/ppm: 173.6, 168.9, 168.6, 148.5, 147.6, 127.1, 123.7, 73.9, 52.8, 52.7, 52.1, 49.8, 49.2, 27.9. MS (20 eV, EI) *m/z* (%): 369 (8) [M]^+^, 317 (39), 300 (29), 282 (16), 246 (21), 152 (31), 145 (29), 131 (100), 86 (46). HRMS (MALDI) for C_16_H_19_NNaO_9_, [M+Na]^+^ (392.0957) found: 369.0968.

*Erythro-methyl 2-(hydroxy(4-nitrophenyl)methyl)-4,4-bis(phenylsulfonyl)butanoate* (**erythro-5**). mp.: 96.1–96.5 °C.^1^H-NMR (400 MHz, CDCl_3_, 25 °C) *δ*/ppm: 8.21 (d, 2H, * J* = 8.6 Hz), 7.88 (d, 2H, * J* = 7.4 Hz), 7.74–7.60 (m, 4H), 7.60–7.49 (m, 4H), 7.45 (t, 2H, *J* = 7.9), 5.26 (t, 1H, *J* = 4.2 Hz), 4.87 (dd, 1H, *J* = 3.2 Hz, 7.9 Hz), 3.60 (s, 3H), 3.41–3.31 (m, 1H), 3.12 (d, *J* = 3.8 Hz), 2.72–2.60 (m, 1H), 2.31–2.19 (m, 1H). ^13^C-NMR (100 MHz, CDCl_3_, 25 °C) *δ*/ppm: 172.7, 147.9, 147.5, 137.4, 136.8, 134.7, 129.6, 129.4, 129.2, 129.0, 127.2, 123.6, 80.6, 72.5, 52.5, 49.7, 22.5. HRMS (ESI) for C_24_H_23_NNaO_9_S_2_, [M+Na]^+^ (556.0712) found: 556.0717. CCDC number: 837000.

*Threo-methyl 2-(hydroxy(4-nitrophenyl)methyl)-4,4-bis(phenylsulfonyl)butanoate* (**threo-5**). mp.: 140.2–140.7 °C. ^1^H-NMR (400 MHz, CDCl_3_, 25 °C) *δ*/ppm: 8.18 (d, 2H, * J* = 8.6 Hz), 7.86 (d, 2H, * J* = 7.4 Hz), 7.81 (d, 2H, * J* = 7.7 Hz), 7.69 (t, 2H,* J* = 7.5 Hz), 7.60–7.49 (m, 4H), 7.47(t, 2H, *J* = 8.6), 5.07 (t, 1H,*J* = 6.0 Hz), 4.73 (dd, 1H, *J* = 4.0 Hz, 7.6 Hz), 3.57 (s, 3H), 3.54–3.45 (m, 1H), 3.36 (d, *J* = 6.5 Hz), 2.54–2.42 (m, 1H), 2.38–2.26 (m, 1H). ^13^C-NMR (100 MHz, CDCl_3_, 25 °C) *δ*/ppm: 172.4, 147.8, 147.6, 137.2, 137.1, 134.8, 134.7, 129.6, 129.5, 129.2, 129.1, 127.2, 123.7, 80.6, 73.4, 52.4, 49.9, 24.8. HRMS (ESI) for C_24_H_23_NNaO_9_S_2_, [M+Na]^+^ (556.0712) found: 556.0710.

*2,2-Diethyl 4-methyl 5-hydroxy-5-(4-nitrophenyl)pentane-2,2,4-tricarboxylate* (**6**). ^1^H-NMR (400 MHz, CDCl_3_, 25 °C) *δ*/ppm: 8.18 (d, 2H, * J* = 8.7 Hz), 7.47 (d, 2H, * J* = 8.7 Hz), 4.99 (*pseudo* t, 1H, *J* = 5.5 Hz), 4.17–4.11 (m, 4H), 3.56 (s, 4H), 2.98–2.97 (m, 1H), 2.31 (dd, 1H, *J* = 14.7, 9.2 Hz), 2.15 (dd, 1H, *J* = 14.7, 2.6 Hz), 1.36 (s, 3H), 1.24–1.17 (m, 6H). ^13^C-NMR (100 MHz, CDCl_3_, 25 °C) *δ*/ppm: 174.0, 171.9, 171.5, 148.8, 147.5, 127.0, 123.5, 74.8, 61.7, 61.6, 52.9, 51.9, 48.6, 34.3, 20.2, 13.9, 13.8. MS (ESI) *m/z* (%): 434 (100) [M+Na]^+^, 305 (5). HRMS (MALDI) for C_19_H_25_NNaO_9_, [M+Na]^+^ (434.1427) found: 434.1438.

## 4. Conclusions

In summary, we have developed a novel, simple approach toward the synthesis of highly functionalized molecules via three-component reactions of substituted aromatic aldehydes, alkyl acrylates and activated alkane catalyzed by ethyl diphenylphosphine. Furthermore, we presented the first organocatalytic addition of carbon-nucleophile to the *in-situ* generated MBH adducts. This multicomponent reaction has a broad reaction scope with all three components of acrylate, aldehyde and activated alkane. Further mechanistic details and development of their asymmetric three-component reactions, are now underway in our laboratory.

## Supplementary Materials

Supplementary materials can be accessed at: http://www.mdpi.com/1420-3049/17/3/2529/s1.
