# Supplementary material for: Tandem Three-Component Reactions of Aldehyde, Alkyl Acrylate, and Dialkylmalonate Catalyzed by Ethyl Diphenylphosphine
Source: Molecules. 2012 Mar 2;17(3):2529–41. doi: 10.3390/molecules17032529 (PMC6268679; doi:10.3390/molecules17032529)
Supplement: Supplementary file 1 [file molecules-17-02529-s001.pdf]

Supporting Materials

I. X-ray Crystallography (*erythro*-5)

X-ray crystal structure (10835.cif) of *erythreo*-**5**: CCDC No. **837000**

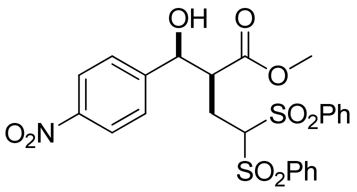

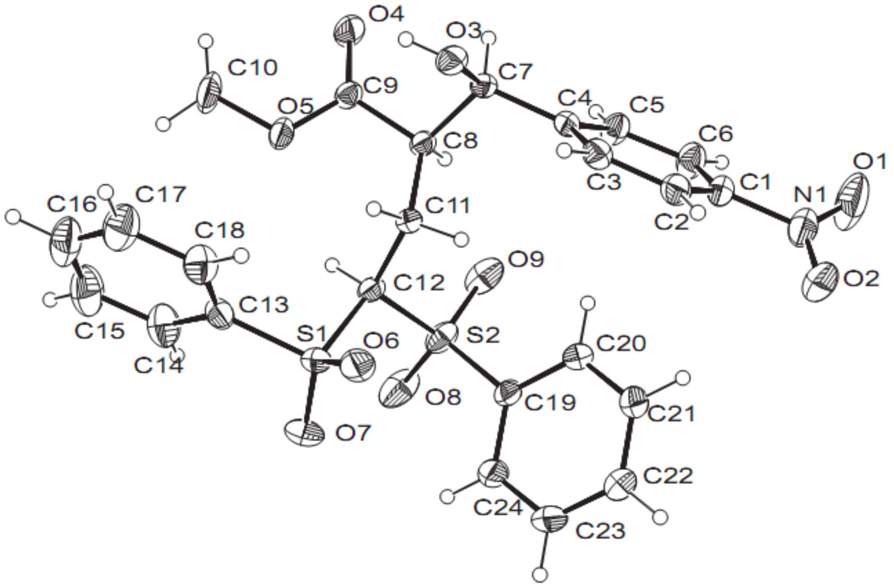

II. ^{1}H-NMR and ^{13}C-NMR Spectra

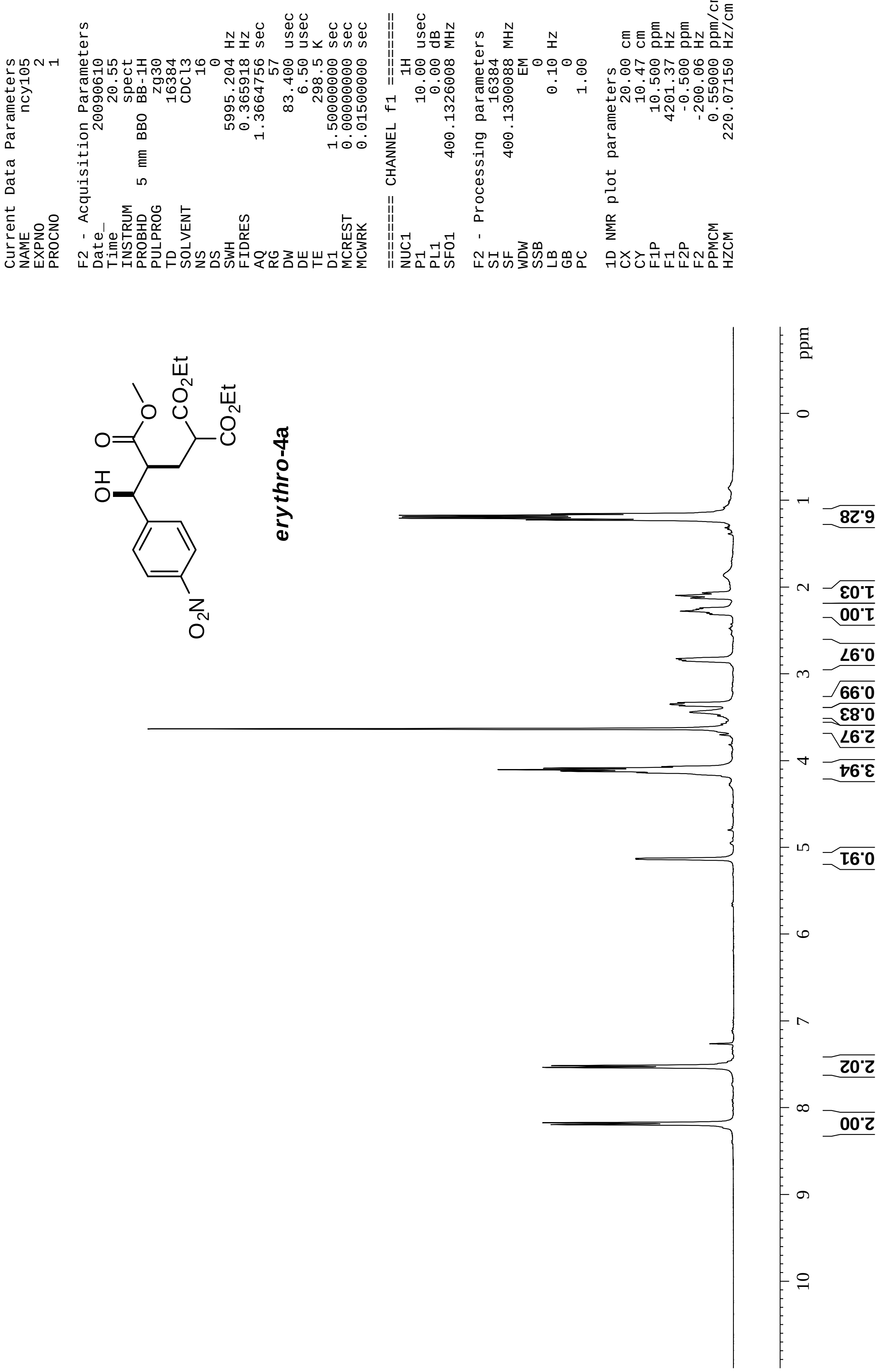

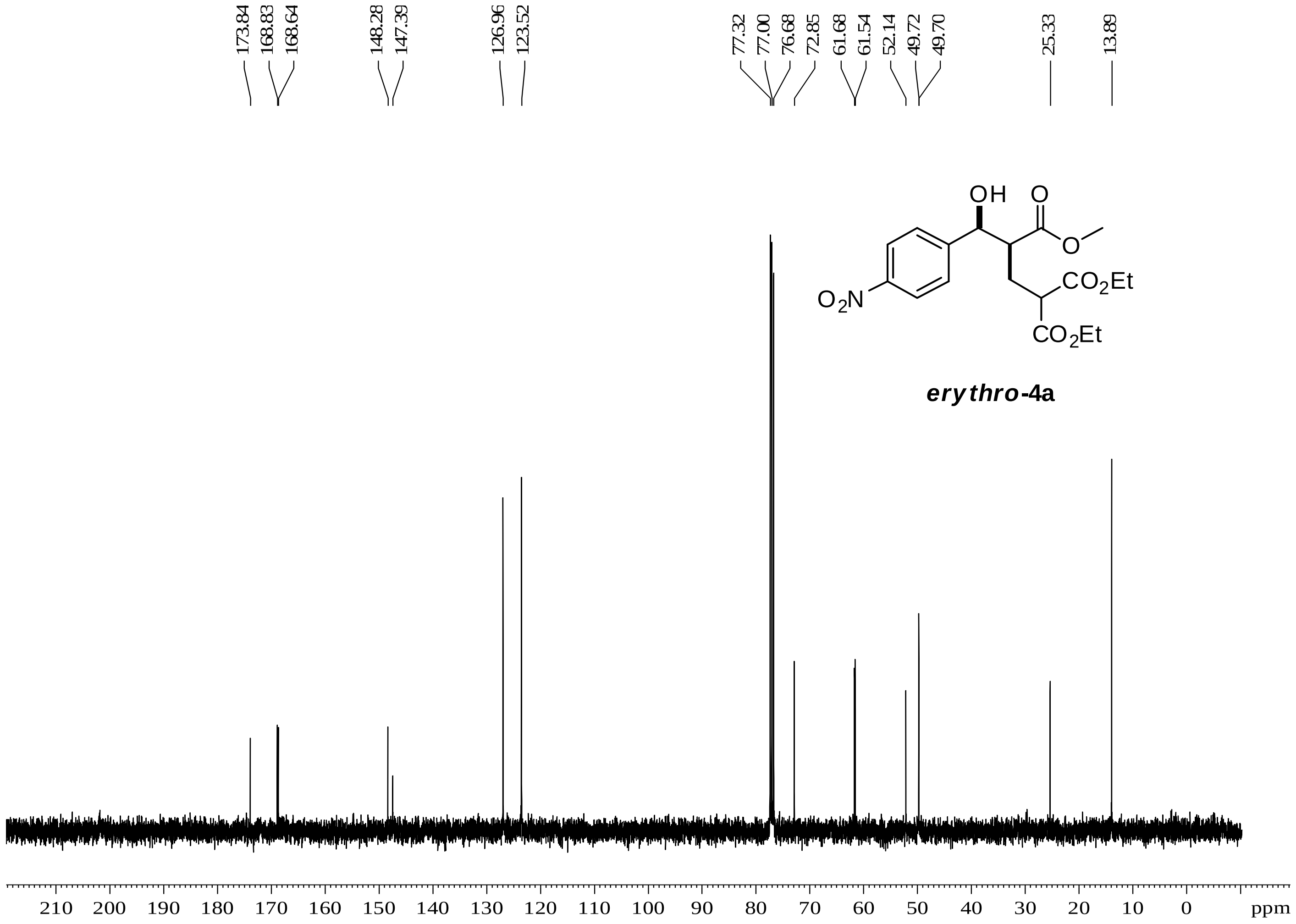


```
Current Data Parameters
NAME              ncy105
EXPNO                  4
PROCNO                 1

F2 - Acquisition Parameters
Date_           20090610
Time               20.57
INSTRUM            spect
PROBHD   5 mm BBO BB-1H
PULPROG          zgpg30
TD                 65536
SOLVENT            CDCl3
NS                    42
DS                     0
SWH           25125.629 Hz
FIDRES         0.383387 Hz
AQ            1.3042164 sec
RG                  4096
DW                19.900 use
DE                  6.50 use
TE                 298.5 K
D1           2.00000000 sec
d11          0.03000000 sec
DELTA        1.89999998 sec
MCREST       0.00000000 sec
MCWRK        0.01500000 sec

======== CHANNEL f1 =======
NUC1                 13C
P1                  8.90 use
PL1                 5.00 dB
SFO1        100.6242995 MHz

======== CHANNEL f2 =======
CPDPRG2          waltz16
NUC2                  1H
PCPD2              90.00 use
PL2                 0.00 dB
PL12               19.10 dB
PL13               22.10 dB
SFO2        400.1319000 MHz

F2 - Processing parameters
SI                 32768
SF          100.6127752 MHz
WDW                   EM
SSB                    0
LB                  1.00 Hz
GB                     0
PC                  1.40

1D NMR plot parameters
CX                 20.00 cm
CY                 11.95 cm
F1P              220.000 ppm
F1              22134.81 Hz
F2P              -10.000 ppm
F2              -1006.13 Hz
PPMCM           11.50000 ppm
HZCM          1157.04688 Hz/
```

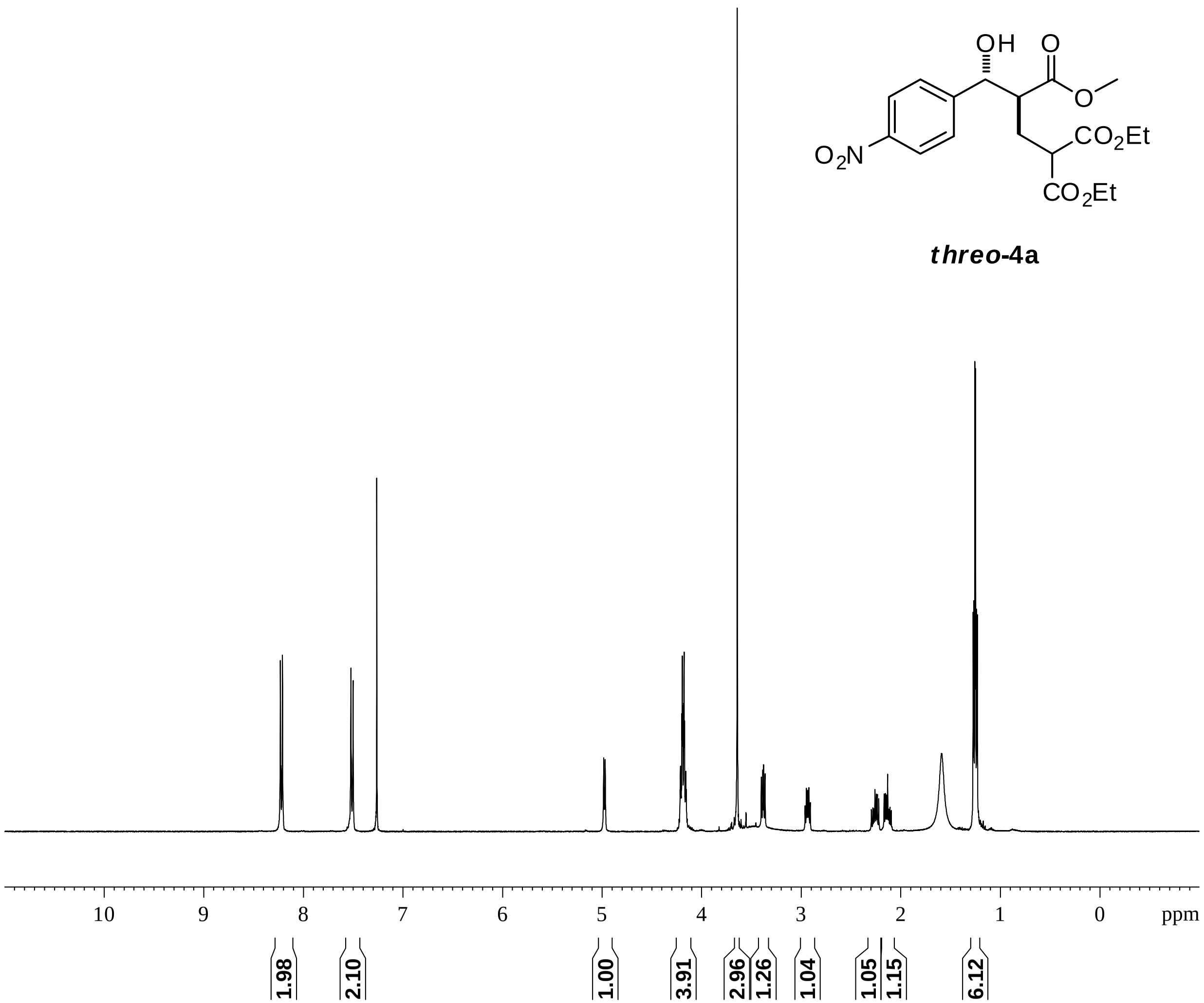

threo-**4a**

```
Current Data Parameters
NAME              ncy083
EXPNO                  8
PROCNO                 1

F2 - Acquisition Parameters
Date_           20090924
Time               16.57
INSTRUM            spect
PROBHD   5 mm BBO BB-1H
PULPROG             zg30
TD                 16384
SOLVENT            CDCl3
NS                    16
DS                     0
SWH             5995.204 Hz
FIDRES          0.365918 Hz
AQ             1.3664756 sec
RG                   256
DW                83.400 usec
DE                  6.50 usec
TE                 299.5 K
D1            1.50000000 sec
MCREST        0.00000000 sec
MCWRK         0.01500000 sec

======== CHANNEL f1 ========
NUC1                  1H
P1                 10.00 usec
PL1                 0.00 dB
SFO1         400.1326008 MHz

F2 - Processing parameters
SI                 16384
SF           400.1300088 MHz
WDW                   EM
SSB                    0
LB                  0.10 Hz
GB                     0
PC                  1.00

1D NMR plot parameters
CX                 20.00 cm
CY                 10.47 cm
F1P               10.500 ppm
F1               4201.37 Hz
F2P               -0.500 ppm
F2               -200.06 Hz
PPMCM            0.55000 ppm/cm
HZCM           220.07150 Hz/cm
```

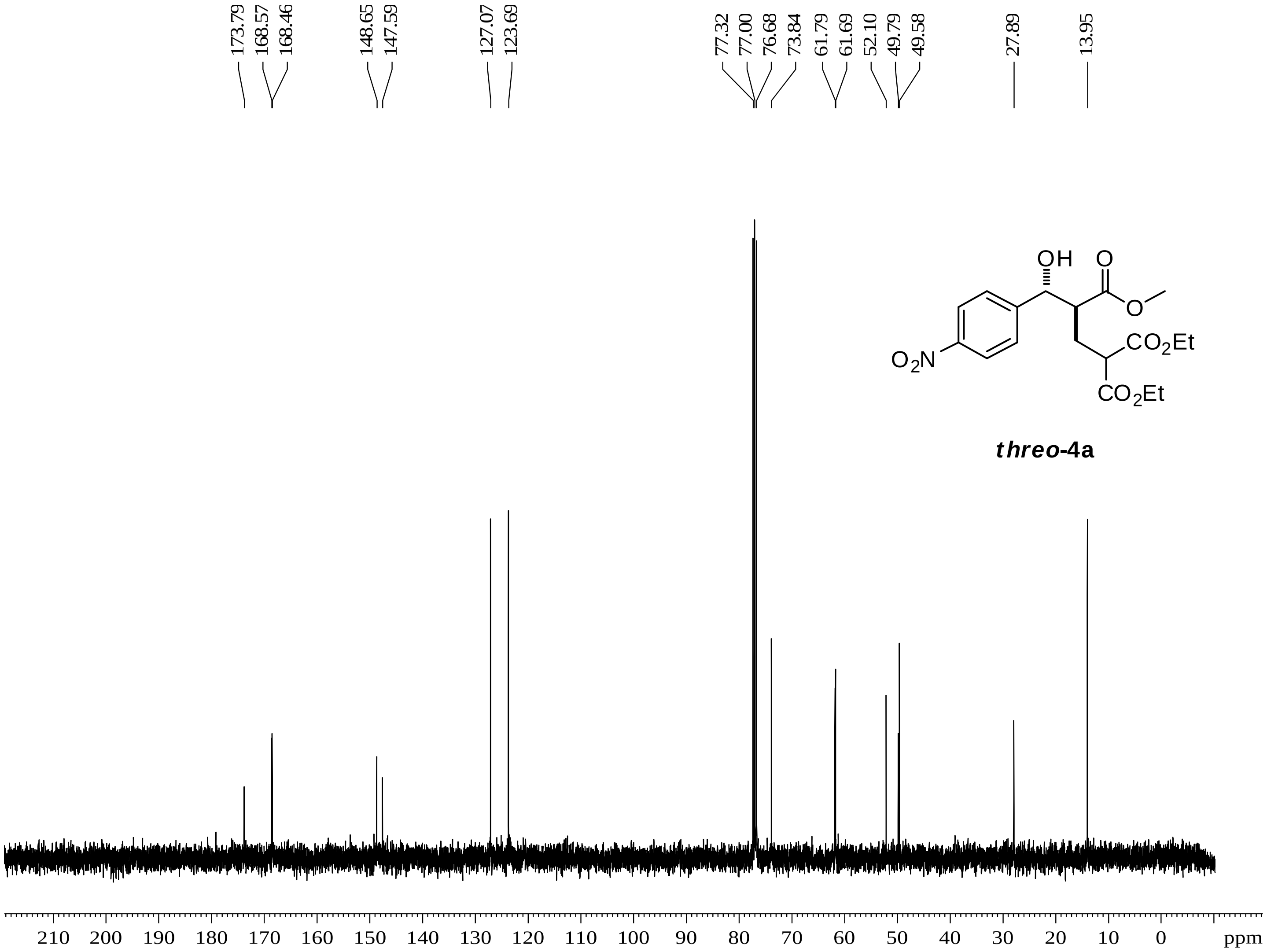

***threo*-4a**

```
Current Data Parameters
NAME             ncy083
EXPNO                 5
PROCNO                1

F2 - Acquisition Parameters
Date_          20090328
Time              13.24
INSTRUM           spect
PROBHD   5 mm BBO BB-1H
PULPROG         zgpg30
TD                65536
SOLVENT           CDCl3
NS                   60
DS                    0
SWH           25125.629 Hz
FIDRES         0.383387 Hz
AQ            1.3042164 sec
RG                 4096
DW               19.900 usec
DE                 6.50 usec
TE                298.7 K
D1           2.00000000 sec
d11          0.03000000 sec
DELTA        1.89999998 sec
MCREST       0.00000000 sec
MCWRK        0.01500000 sec

======== CHANNEL f1 ========
NUC1                13C
P1                 8.90 usec
PL1                5.00 dB
SFO1        100.6242995 MHz

======== CHANNEL f2 ========
CPDPRG2         waltz16
NUC2                 1H
PCPD2             90.00 usec
PL2                0.00 dB
PL12              19.10 dB
PL13              22.10 dB
SFO2        400.1319000 MHz

F2 - Processing parameters
SI                32768
SF          100.6127736 MHz
WDW                  EM
SSB                   0
LB                 1.00 Hz
GB                    0
PC                 1.40

1D NMR plot parameters
CX                20.00 cm
CY                11.95 cm
F1P             220.000 ppm
F1             22134.81 Hz
```

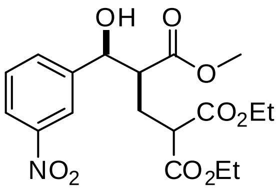

***erythro*-4b**

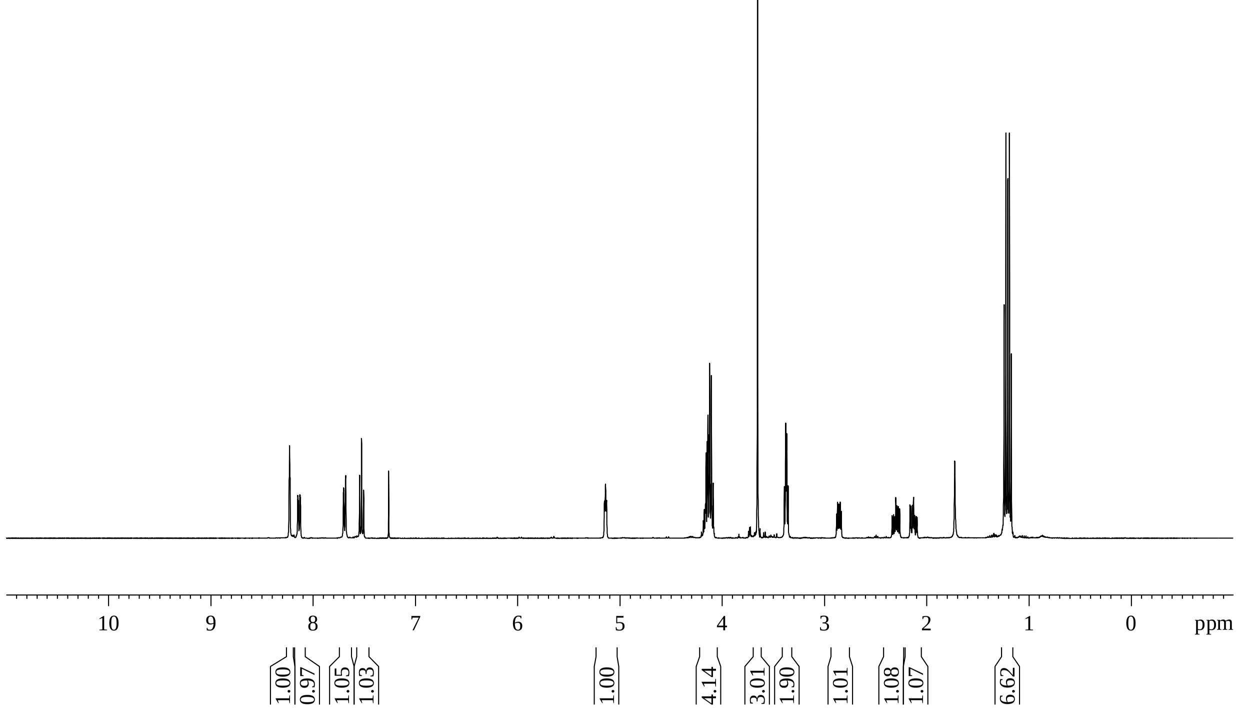


```
Current Data Parameters
NAME             ncy123
EXPNO                 5
PROCNO                1

F2 - Acquisition Parameters
Date_          20090702
Time              14.08
INSTRUM           spect
PROBHD   5 mm BBO BB-1H
PULPROG            zg30
TD                16384
SOLVENT           CDCl3
NS                   16
DS                    0
SWH            5995.204 Hz
FIDRES         0.365918 Hz
AQ            1.3664756 sec
RG                 90.5
DW               83.400 usec
DE                 6.50 usec
TE                299.8 K
D1           1.50000000 sec
MCREST       0.00000000 sec
MCWRK        0.01500000 sec

======== CHANNEL f1 ========
NUC1                 1H
P1                10.00 usec
PL1                0.00 dB
SFO1        400.1326008 MHz

F2 - Processing parameters
SI                16384
SF          400.1300088 MHz
WDW                  EM
SSB                   0
LB                 0.10 Hz
GB                    0
PC                 1.00

1D NMR plot parameters
CX                20.00 cm
CY                10.45 cm
F1P              10.500 ppm
F1              4201.37 Hz
F2P              -0.500 ppm
F2              -200.06 Hz
PPMCM           0.55000 ppm/cm
HZCM          220.07150 Hz/cm
```

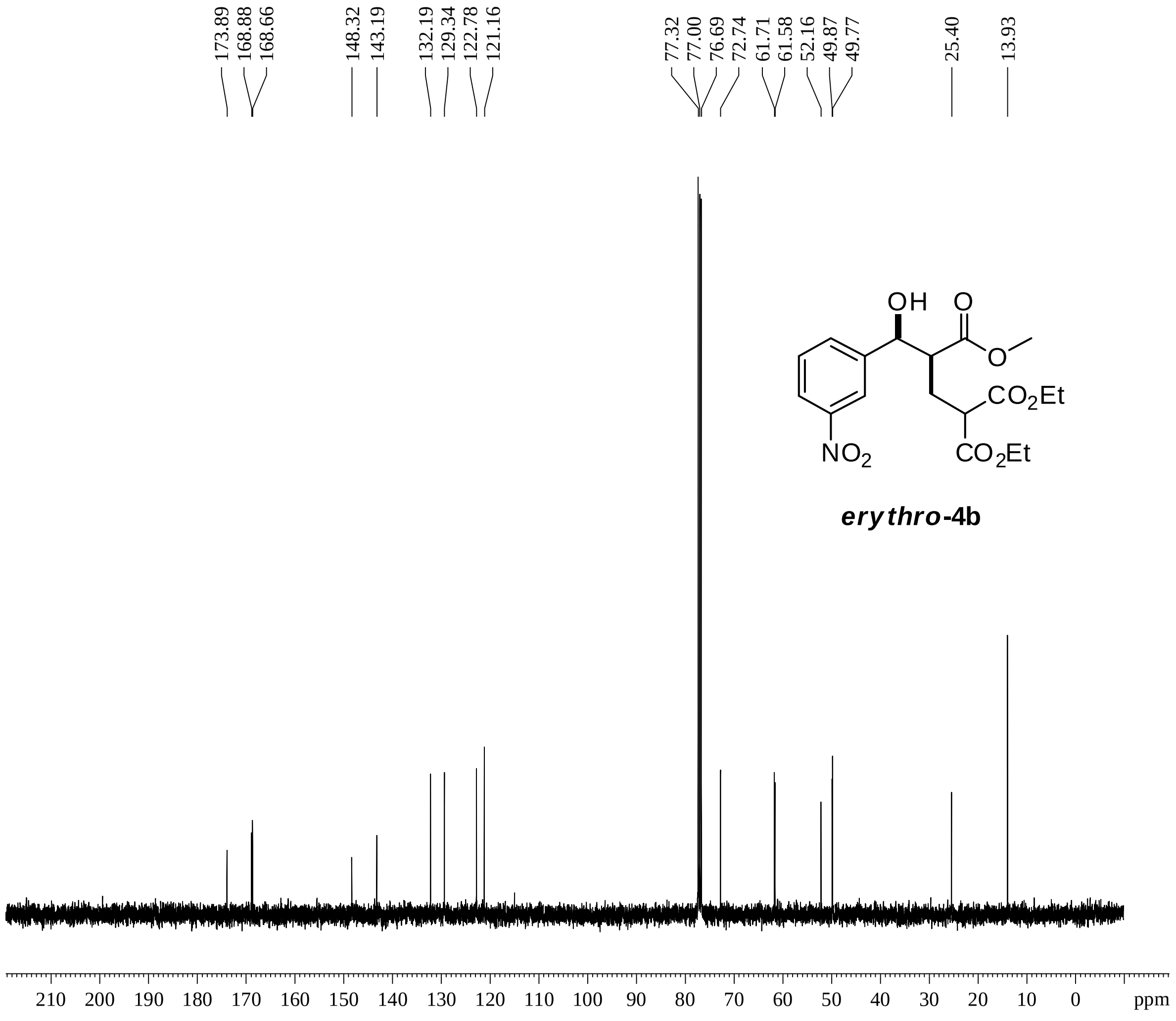


```
Current Data Parameters
NAME             ncy123
EXPNO                 6
PROCNO                1

F2 - Acquisition Parameters
Date_          20090702
Time              14.12
INSTRUM           spect
PROBHD   5 mm BBO BB-1H
PULPROG          zgpg30
TD                65536
SOLVENT           CDCl3
NS                   89
DS                    0
SWH           25125.629 Hz
FIDRES         0.383387 Hz
AQ            1.3042164 sec
RG                 4096
DW               19.900 usec
DE                 6.50 usec
TE                300.3 K
D1           2.00000000 sec
d11          0.03000000 sec
DELTA        1.89999998 sec
MCREST       0.00000000 sec
MCWRK        0.01500000 sec

======== CHANNEL f1 ========
NUC1                13C
P1                 8.90 usec
PL1                5.00 dB
SFO1        100.6243390 MHz

======== CHANNEL f2 ========
CPDPRG2         waltz16
NUC2                 1H
PCPD2             90.00 usec
PL2                0.00 dB
PL12              19.10 dB
PL13              22.10 dB
SFO2        400.1319000 MHz

F2 - Processing parameters
SI                32768
SF          100.6127717 MHz
WDW                  EM
SSB                   0
LB                 1.00 Hz
GB                    0
PC                 1.40

1D NMR plot parameters
CX                20.00 cm
CY                11.89 cm
F1P             220.000 ppm
F1             22134.81 Hz
```

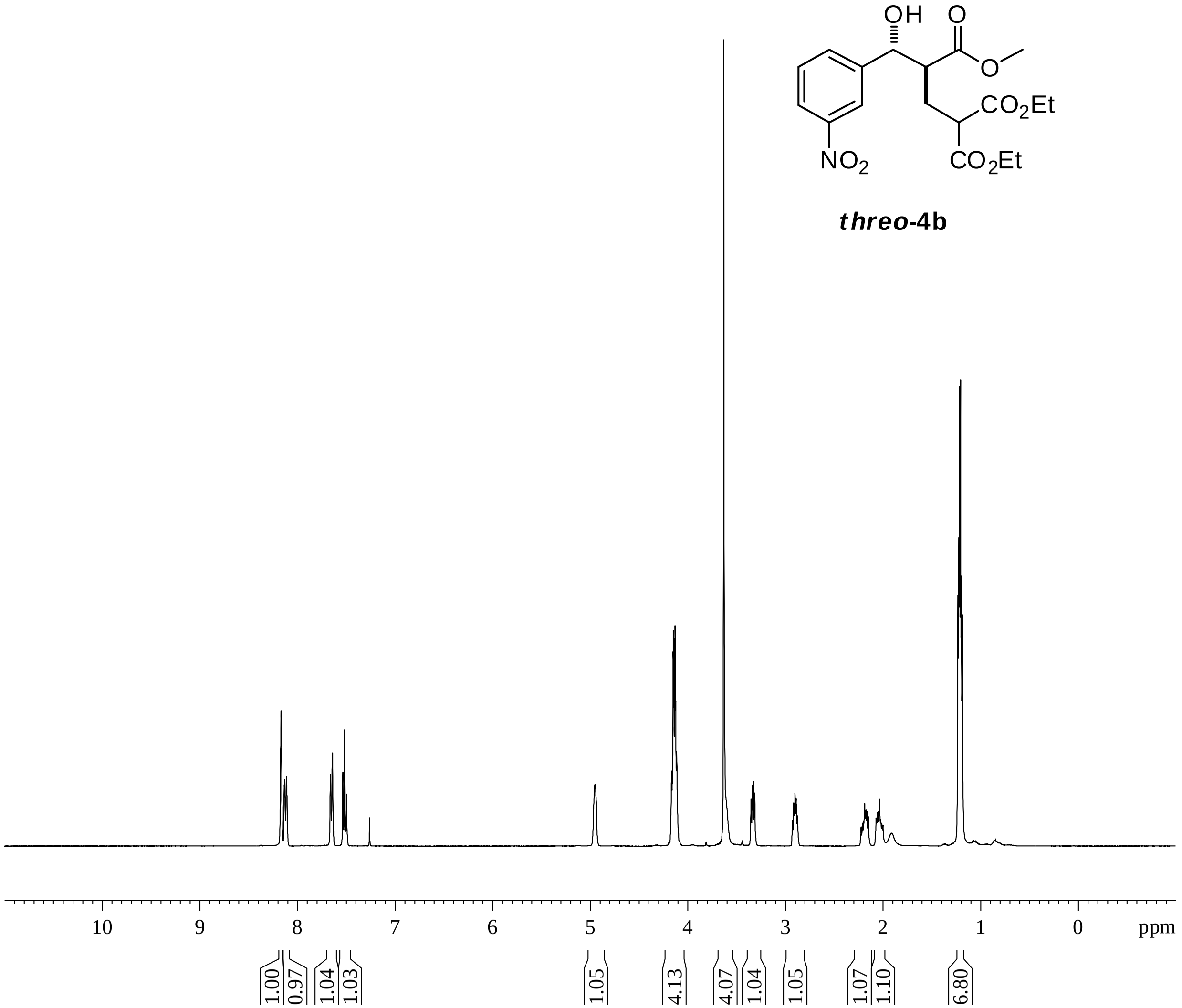

***threo*-4b**

```
Current Data Parameters
NAME              ncy123
EXPNO                  9
PROCNO                 1

F2 - Acquisition Parameters
Date_           20090705
Time               13.01
INSTRUM            spect
PROBHD   5 mm BBO BB-1H
PULPROG             zg30
TD                 16384
SOLVENT            CDCl3
NS                    16
DS                     0
SWH             5995.204 Hz
FIDRES          0.365918 Hz
AQ             1.3664756 sec
RG                  45.3
DW                83.400 usec
DE                  6.50 usec
TE                 300.2 K
D1            1.50000000 sec
MCREST        0.00000000 sec
MCWRK         0.01500000 sec

======== CHANNEL f1 ========
NUC1                  1H
P1                 10.00 usec
PL1                 0.00 dB
SFO1         400.1326008 MHz

F2 - Processing parameters
SI                 16384
SF           400.1300084 MHz
WDW                   EM
SSB                    0
LB                  0.10 Hz
GB                     0
PC                  1.00

1D NMR plot parameters
CX                 20.00 cm
CY                 10.45 cm
F1P               10.500 ppm
F1               4201.37 Hz
F2P               -0.500 ppm
F2               -200.06 Hz
PPMCM            0.55000 ppm/cm
HZCM           220.07150 Hz/cm
```

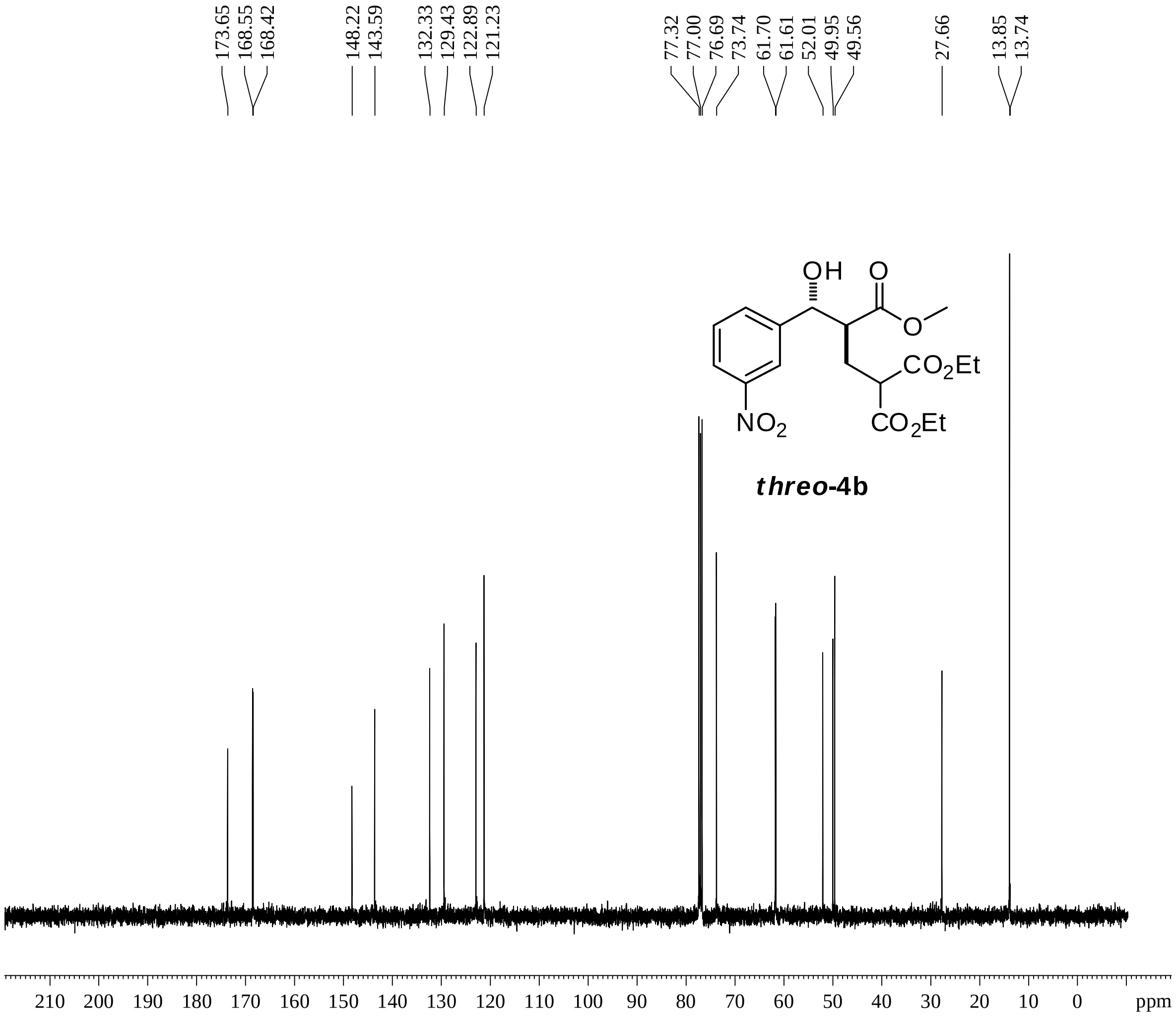

***threo*-4b**

```
Current Data Parameters
NAME             ncy123
EXPNO                 4
PROCNO                1

F2 - Acquisition Parameters
Date_          20090617
Time              20.59
INSTRUM           spect
PROBHD   5 mm BBO BB-1H
PULPROG          zgpg30
TD                65536
SOLVENT           CDCl3
NS                   80
DS                    0
SWH           25125.629 Hz
FIDRES         0.383387 Hz
AQ            1.3042164 sec
RG                 4096
DW               19.900 usec
DE                 6.50 usec
TE                300.2 K
D1           2.00000000 sec
d11          0.03000000 sec
DELTA        1.89999998 sec
MCREST       0.00000000 sec
MCWRK        0.01500000 sec

======== CHANNEL f1 ========
NUC1                13C
P1                 8.90 usec
PL1                5.00 dB
SFO1        100.6242995 MHz

======== CHANNEL f2 ========
CPDPRG2         waltz16
NUC2                 1H
PCPD2             90.00 usec
PL2                0.00 dB
PL12              19.10 dB
PL13              22.10 dB
SFO2        400.1319000 MHz

F2 - Processing parameters
SI                32768
SF          100.6127782 MHz
WDW                  EM
SSB                   0
LB                 1.00 Hz
GB                    0
PC                 1.40

1D NMR plot parameters
CX                20.00 cm
CY                11.91 cm
F1P             220.000 ppm
F1             22134.81 Hz
```

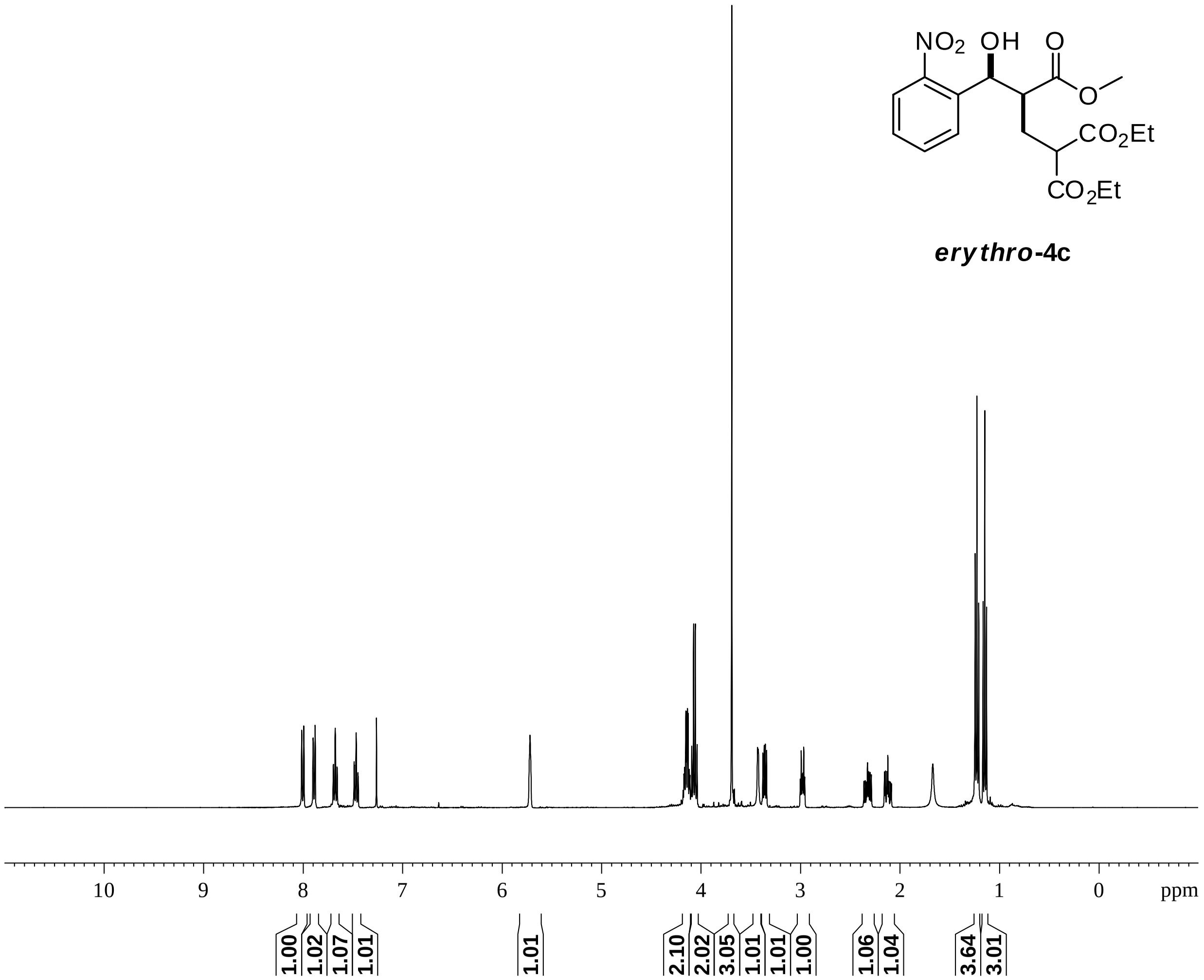

***erythro*-4c**

```
Current Data Parameters
NAME             ncy124
EXPNO                 3
PROCNO                1

F2 - Acquisition Parameters
Date_          20090704
Time              13.31
INSTRUM           spect
PROBHD   5 mm BBO BB-1H
PULPROG            zg30
TD                16384
SOLVENT           CDCl3
NS                   16
DS                    0
SWH            5995.204 Hz
FIDRES         0.365918 Hz
AQ            1.3664756 sec
RG                  114
DW               83.400 usec
DE                 6.50 usec
TE                299.8 K
D1           1.50000000 sec
MCREST       0.00000000 sec
MCWRK        0.01500000 sec

======== CHANNEL f1 ========
NUC1                 1H
P1                10.00 usec
PL1                0.00 dB
SFO1        400.1326008 MHz

F2 - Processing parameters
SI                16384
SF          400.1300088 MHz
WDW                  EM
SSB                   0
LB                 0.10 Hz
GB                    0
PC                 1.00

1D NMR plot parameters
CX                20.00 cm
CY                10.47 cm
F1P              10.500 ppm
F1              4201.37 Hz
F2P              -0.500 ppm
F2              -200.06 Hz
PPMCM           0.55000 ppm/cm
HZCM          220.07150 Hz/cm
```

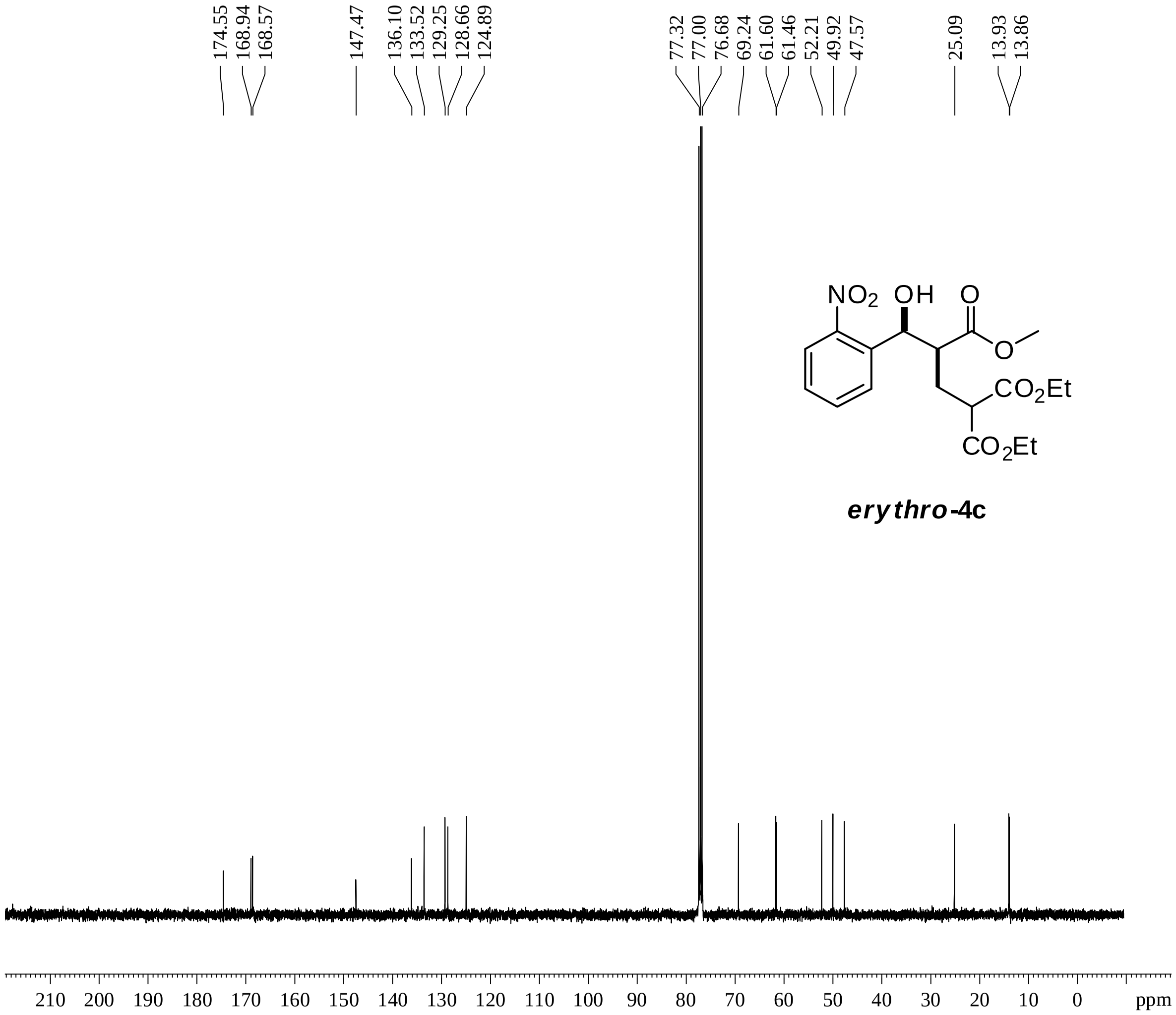

***erythro*-4c**

```
Current Data Parameters
NAME              ncy124
EXPNO                 10
PROCNO                 1

F2 - Acquisition Parameters
Date_           20090719
Time               14.55
INSTRUM            spect
PROBHD   5 mm BBO BB-1H
PULPROG          zgpg30
TD                 65536
SOLVENT            CDCl3
NS                   282
DS                     0
SWH          25062.656 Hz
FIDRES        0.382426 Hz
AQ           1.3074932 sec
RG                  4096
DW               19.950 usec
DE                 6.50 usec
TE                299.7 K
D1         2.00000000 sec
d11        0.03000000 sec
DELTA      1.89999998 sec
MCREST     0.00000000 sec
MCWRK      0.01500000 sec

======== CHANNEL f1 ========
NUC1                 13C
P1                  8.90 usec
PL1                 5.00 dB
SFO1         100.6243390 MHz

======== CHANNEL f2 ========
CPDPRG2          waltz16
NUC2                  1H
PCPD2             100.00 usec
PL2                 0.00 dB
PL12               19.10 dB
PL13               22.10 dB
SFO2         400.1319000 MHz

F2 - Processing parameters
SI                 32768
SF          100.6127729 MHz
WDW                   EM
SSB                    0
LB                  1.00 Hz
GB                     0
PC                  1.40

1D NMR plot parameters
CX                 20.00 cm
CY                 32.21 cm
F1P              220.000 ppm
F1              22134.81 Hz
```

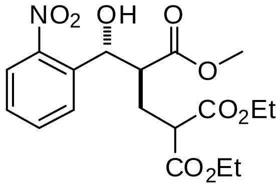

***threo*-4c**

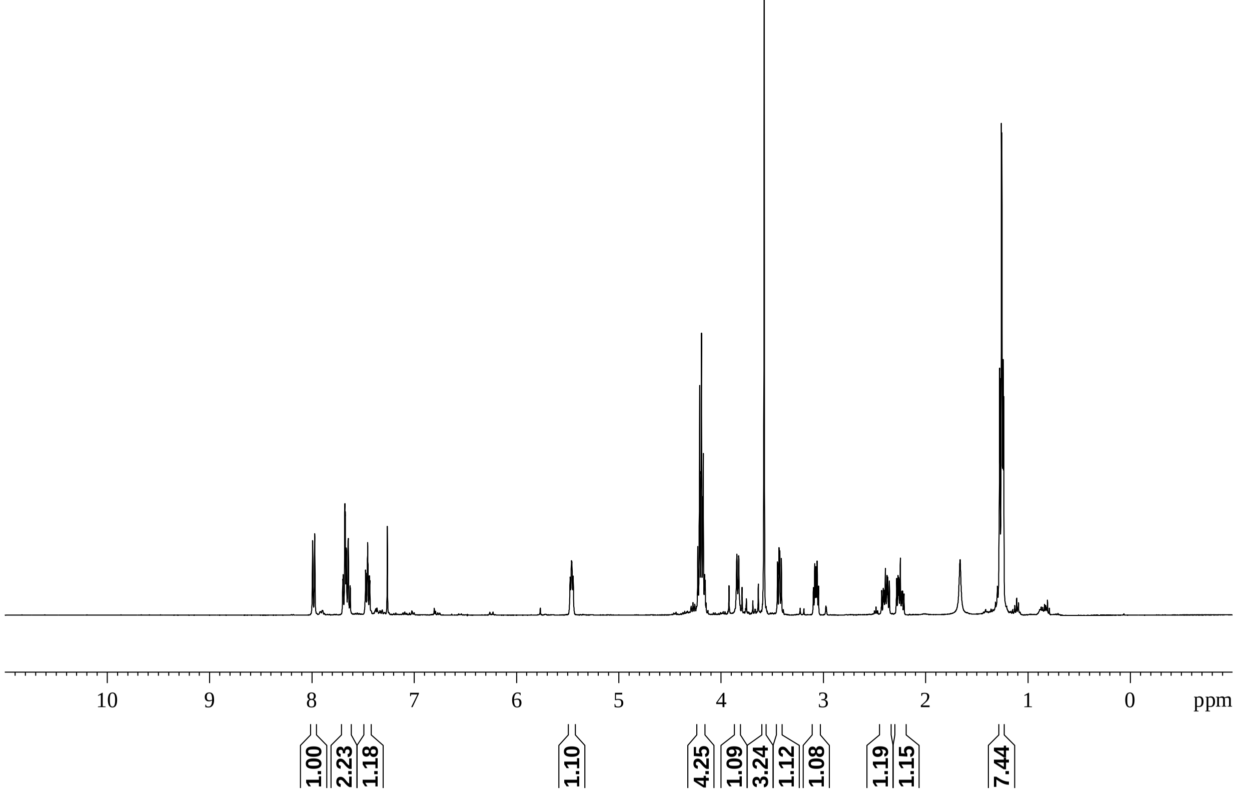


```
Current Data Parameters
NAME              ncy124
EXPNO                  7
PROCNO                 1

F2 - Acquisition Parameters
Date_           20090708
Time               10.32
INSTRUM            spect
PROBHD   5 mm BBO BB-1H
PULPROG             zg30
TD                 16384
SOLVENT            CDCl3
NS                    16
DS                     0
SWH             5995.204 Hz
FIDRES          0.365918 Hz
AQ             1.3664756 sec
RG                  90.5
DW                83.400 usec
DE                  6.50 usec
TE                 301.3 K
D1            1.50000000 sec
MCREST        0.00000000 sec
MCWRK         0.01500000 sec

======== CHANNEL f1 ========
NUC1                  1H
P1                 10.00 usec
PL1                 0.00 dB
SFO1         400.1326008 MHz

F2 - Processing parameters
SI                 16384
SF           400.1300088 MHz
WDW                   EM
SSB                    0
LB                  0.10 Hz
GB                     0
PC                  1.00

1D NMR plot parameters
CX                 20.00 cm
CY                  9.97 cm
F1P               10.500 ppm
F1               4201.37 Hz
F2P               -0.500 ppm
F2               -200.06 Hz
PPMCM            0.55000 ppm/cm
HZCM            220.07150 Hz/cm
```

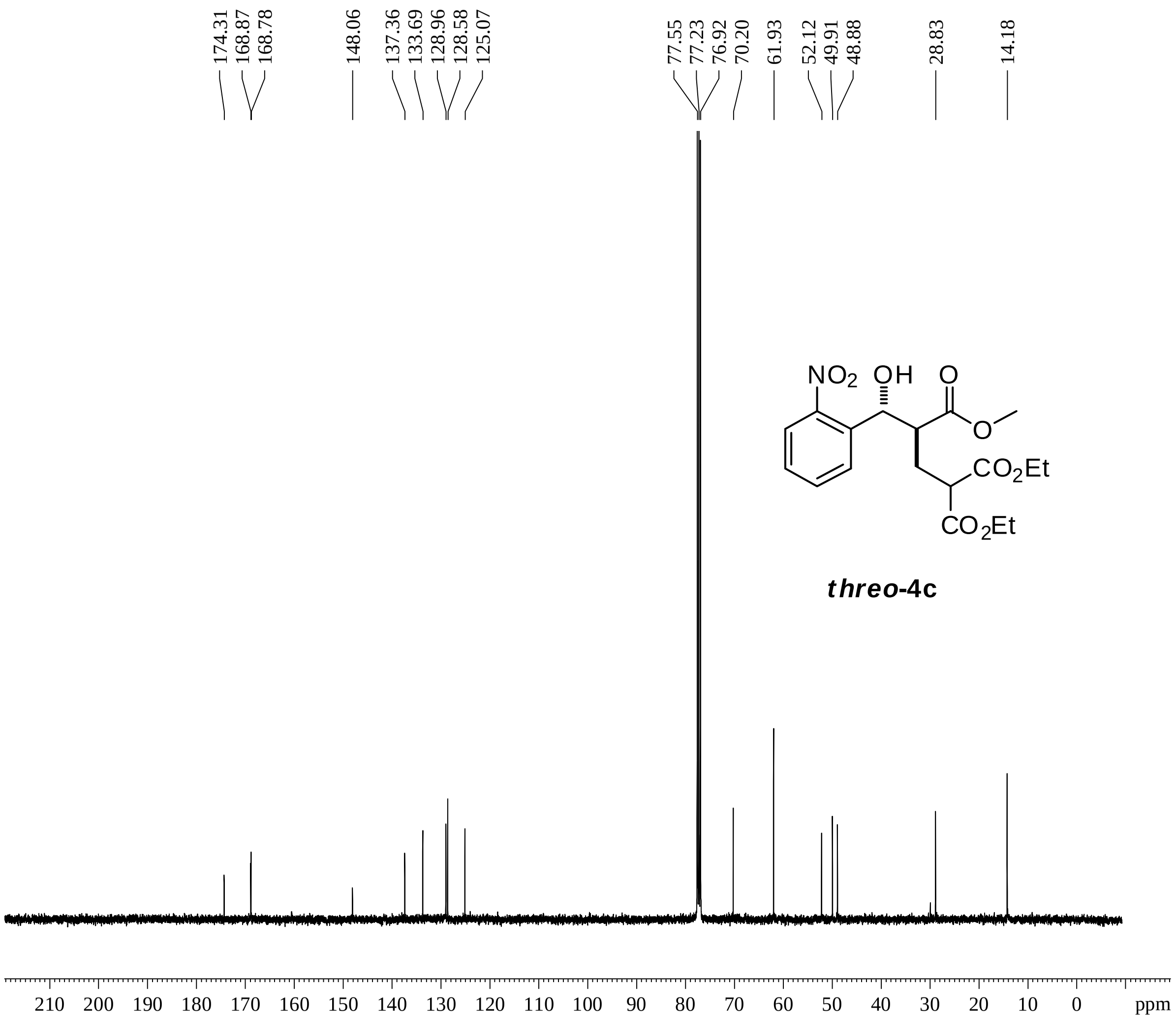

***threo*-4c**

```
Current Data Parameters
NAME             ncy124
EXPNO                14
PROCNO                1

F2 - Acquisition Parameters
Date_          20090820
Time              18.46
INSTRUM           spect
PROBHD   5 mm BBO BB-1H
PULPROG         zgpg30
TD                65536
SOLVENT           CDCl3
NS                  615
DS                    0
SWH           25062.656 Hz
FIDRES         0.382426 Hz
AQ            1.3074932 sec
RG                 4096
DW               19.950 use
DE                 6.50 use
TE                298.4 K
D1           2.00000000 sec
d11          0.03000000 sec
DELTA        1.89999998 sec
MCREST       0.00000000 sec
MCWRK        0.01500000 sec

======== CHANNEL f1 =======
NUC1                13C
P1                 8.90 use
PL1                5.00 dB
SFO1        100.6243390 MHz

======== CHANNEL f2 =======
CPDPRG2         waltz16
NUC2                 1H
PCPD2            100.00 use
PL2                0.00 dB
PL12              19.10 dB
PL13              22.10 dB
SFO2        400.1319000 MHz

F2 - Processing parameters
SI                32768
SF          100.6127505 MHz
WDW                  EM
SSB                   0
LB                 1.00 Hz
GB                    0
PC                 1.40

1D NMR plot parameters
CX                20.00 cm
CY                12.03 cm
F1P             220.000 ppm
F1             22134.80 Hz
```

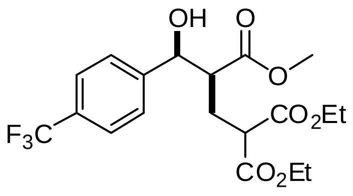

***erythro*-4d**

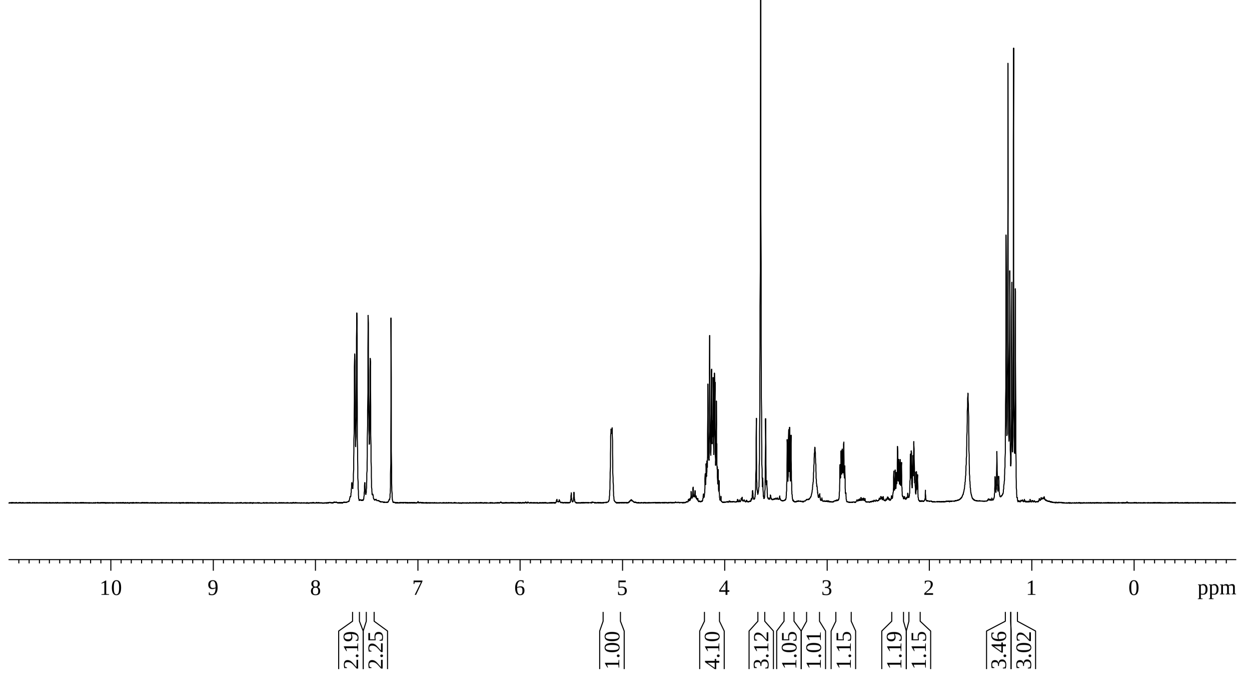


```
Current Data Parameters
NAME              ncy115
EXPNO                  7
PROCNO                 1

F2 - Acquisition Parameters
Date_           20090620
Time               16.00
INSTRUM            spect
PROBHD   5 mm BBO BB-1H
PULPROG             zg30
TD                 16384
SOLVENT            CDCl3
NS                    16
DS                     0
SWH             5995.204 Hz
FIDRES          0.365918 Hz
AQ             1.3664756 sec
RG                 161.3
DW                83.400 usec
DE                  6.50 usec
TE                 299.7 K
D1            1.50000000 sec
MCREST        0.00000000 sec
MCWRK         0.01500000 sec

======== CHANNEL f1 ========
NUC1                  1H
P1                 10.00 usec
PL1                 0.00 dB
SFO1         400.1326008 MHz

F2 - Processing parameters
SI                 16384
SF           400.1300091 MHz
WDW                   EM
SSB                    0
LB                  0.10 Hz
GB                     0
PC                  1.00

1D NMR plot parameters
CX                 20.00 cm
CY                 10.00 cm
F1P               10.500 ppm
F1               4201.37 Hz
F2P               -0.500 ppm
F2               -200.06 Hz
PPMCM            0.55000 ppm/cm
HZCM           220.07150 Hz/cm
```

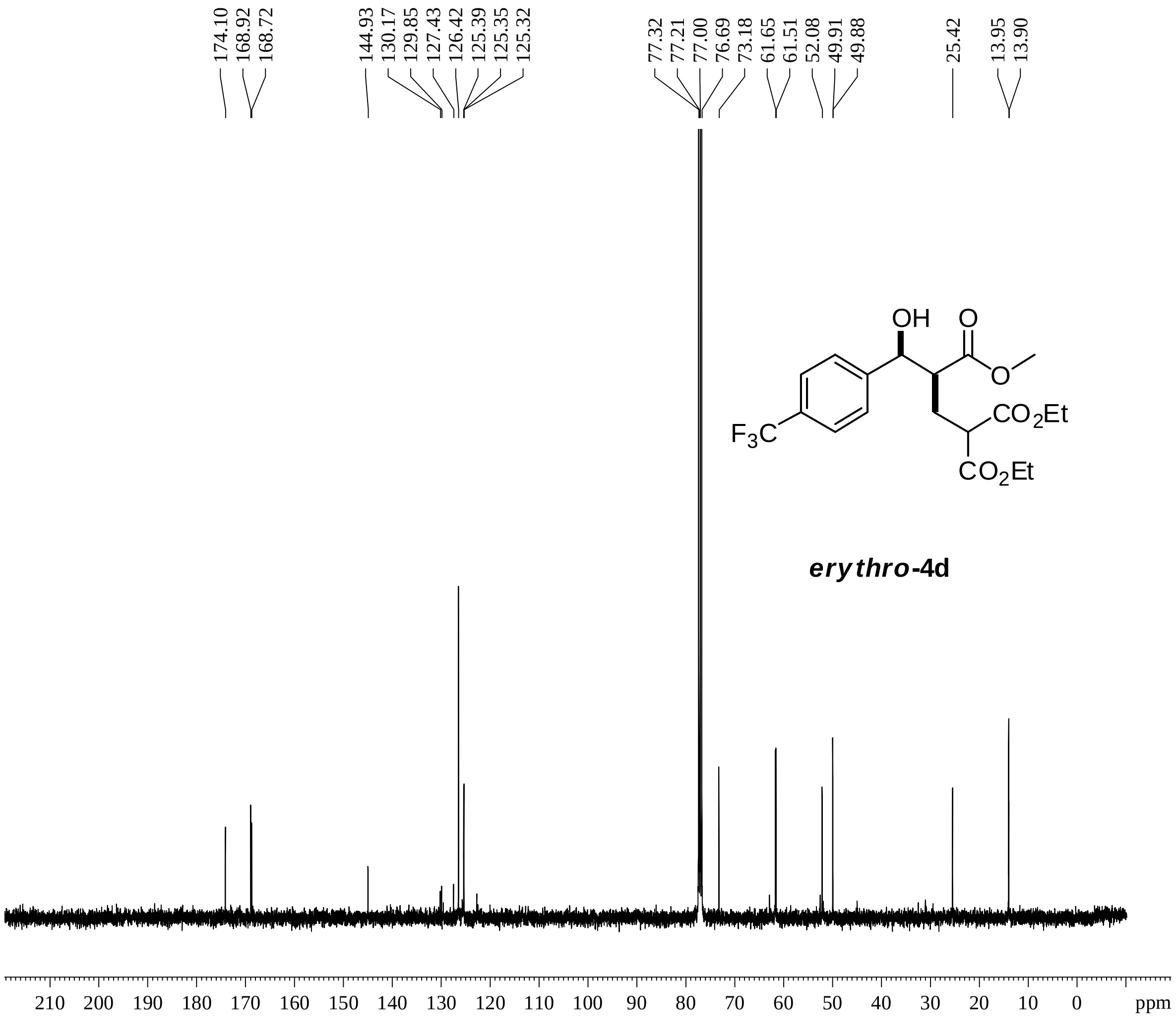

***erythro*-4d**

```
Current Data Parameters
NAME              ncy115
EXPNO                  8
PROCNO                 1

F2 - Acquisition Parameters
Date_           20090620
Time               16.04
INSTRUM            spect
PROBHD   5 mm BBO BB-1H
PULPROG          zgpg30
TD                 65536
SOLVENT            CDCl3
NS                   818
DS                     0
SWH            25125.629 Hz
FIDRES          0.383387 Hz
AQ             1.3042164 sec
RG                  4096
DW                19.900 usec
DE                  6.50 usec
TE                 300.1 K
D1            2.00000000 sec
d11           0.03000000 sec
DELTA         1.89999998 sec
MCREST        0.00000000 sec
MCWRK         0.01500000 sec

======== CHANNEL f1 ========
NUC1                 13C
P1                  8.90 usec
PL1                 5.00 dB
SFO1         100.6242995 MHz

======== CHANNEL f2 ========
CPDPRG2          waltz16
NUC2                  1H
PCPD2              90.00 usec
PL2                 0.00 dB
PL12               19.10 dB
PL13               22.10 dB
SFO2         400.1319000 MHz

F2 - Processing parameters
SI                 32768
SF           100.6127698 MHz
WDW                   EM
SSB                    0
LB                  1.00 Hz
GB                     0
PC                  1.40

1D NMR plot parameters
CX                 20.00 cm
CY                 17.17 cm
F1P              220.000 ppm
F1              22134.81 Hz
```

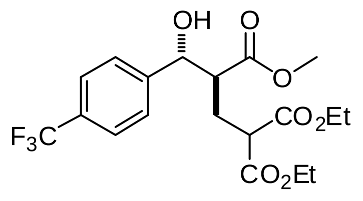

***threo*-4d**

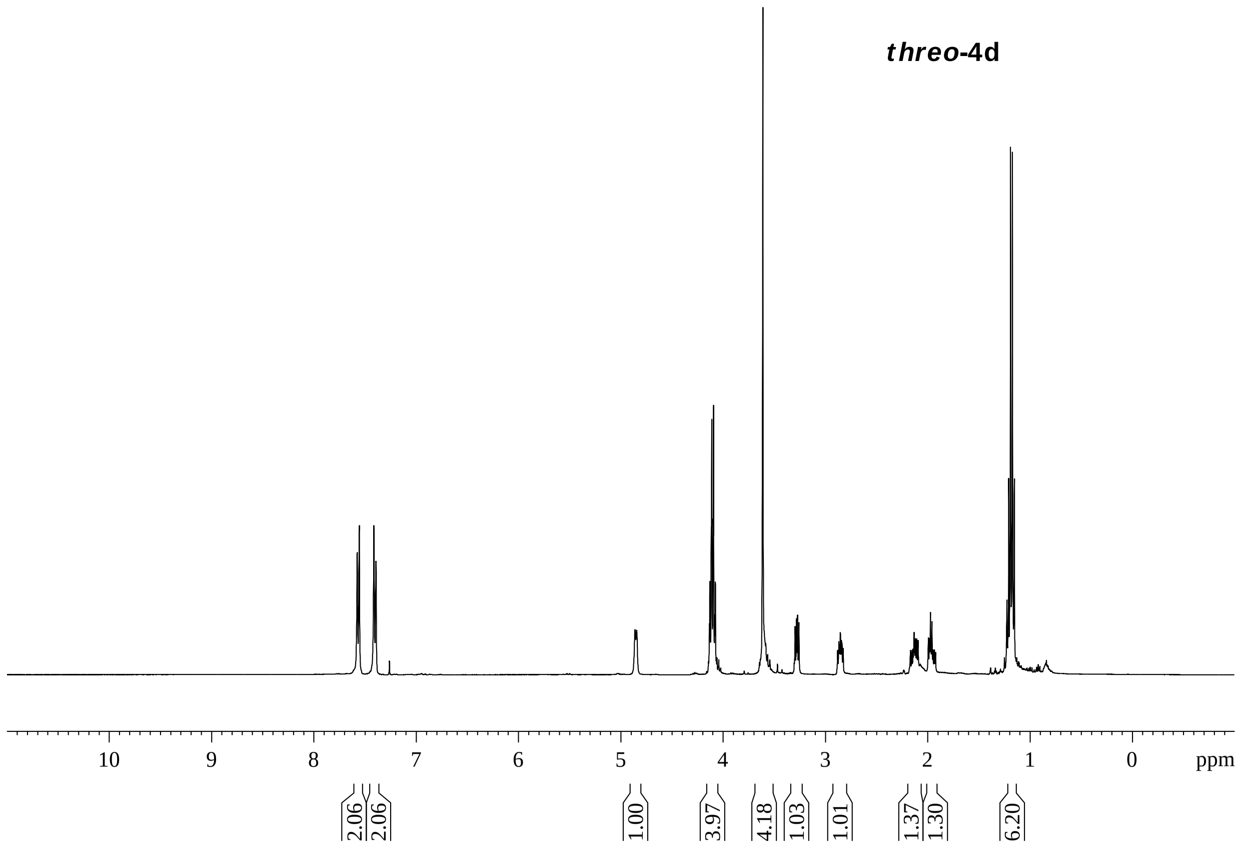


```
Current Data Parameters
NAME              ncy115
EXPNO                  5
PROCNO                 1

F2 - Acquisition Parameters
Date_           20090522
Time               22.41
INSTRUM            spect
PROBHD   5 mm BBO BB-1H
PULPROG             zg30
TD                 16384
SOLVENT            CDCl3
NS                    16
DS                     0
SWH             5995.204 Hz
FIDRES          0.365918 Hz
AQ             1.3664756 sec
RG                  22.6
DW                83.400 usec
DE                  6.50 usec
TE                 299.6 K
D1            1.50000000 sec
MCREST        0.00000000 sec
MCWRK         0.01500000 sec

======== CHANNEL f1 ========
NUC1                  1H
P1                 10.00 usec
PL1                 0.00 dB
SFO1         400.1326008 MHz

F2 - Processing parameters
SI                 16384
SF           400.1300088 MHz
WDW                   EM
SSB                    0
LB                  0.10 Hz
GB                     0
PC                  1.00

1D NMR plot parameters
CX                 20.00 cm
CY                 10.00 cm
F1P               10.500 ppm
F1               4201.37 Hz
F2P               -0.500 ppm
F2               -200.06 Hz
PPMCM            0.55000 ppm/cm
HZCM           220.07150 Hz/cm
```

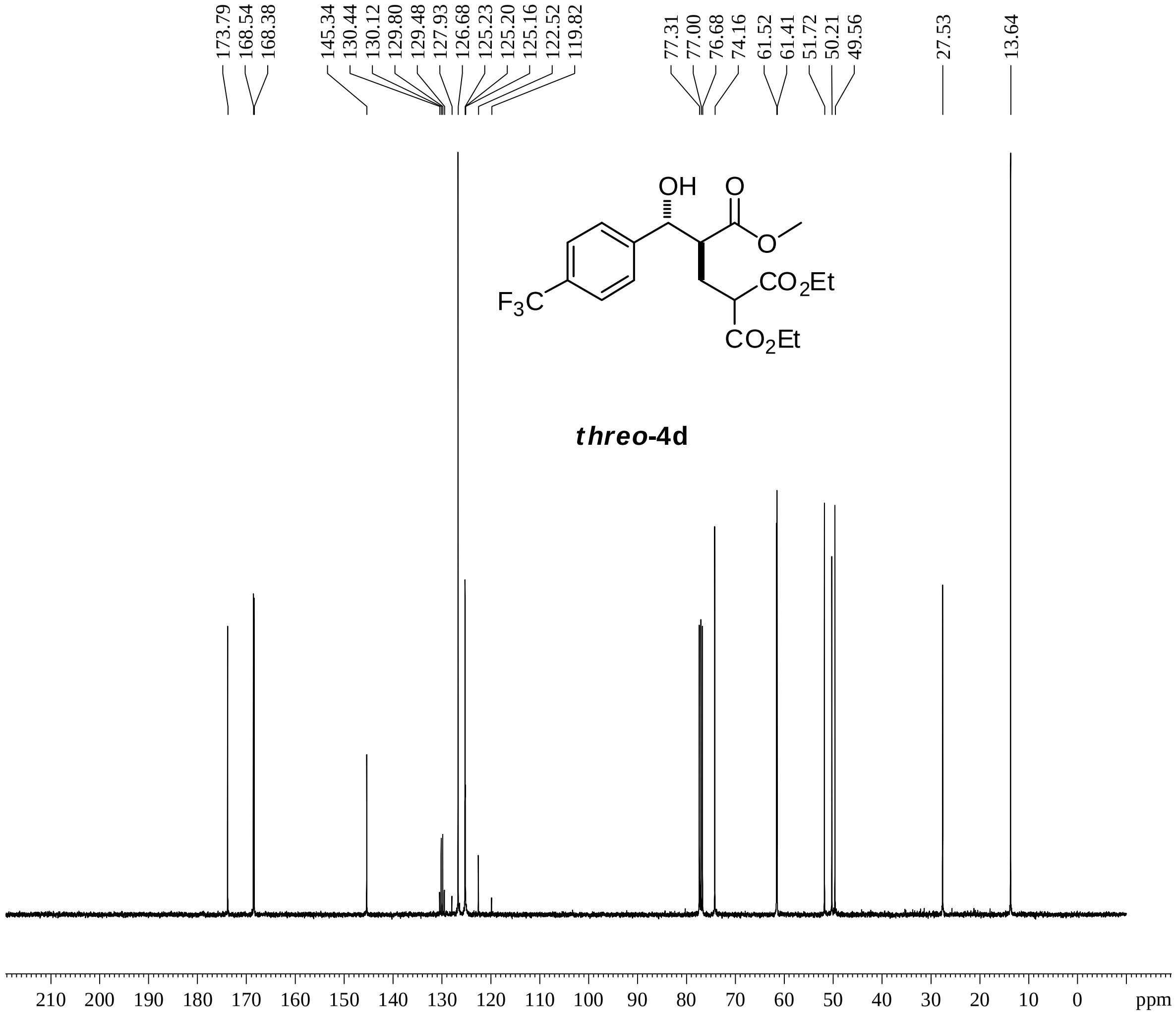


```
Current Data Parameters
NAME            ncy252
EXPNO                9
PROCNO               1

F2 - Acquisition Parameters
Date_         20100606
Time             18.21
INSTRUM          spect
PROBHD   5 mm BBO BB-1H
PULPROG         zgpg30
TD               65536
SOLVENT          CDCl3
NS                 413
DS                   0
SWH          25062.656 Hz
FIDRES        0.382426 Hz
AQ           1.3074932 sec
RG                4096
DW              19.950 usec
DE                6.50 usec
TE               301.2 K
D1          2.00000000 sec
d11         0.03000000 sec
DELTA       1.89999998 sec
MCREST      0.00000000 sec
MCWRK       0.01500000 sec

======== CHANNEL f1 ========
NUC1               13C
P1               10.00 usec
PL1               5.00 dB
SFO1       100.6242995 MHz

======== CHANNEL f2 ========
CPDPRG2        waltz16
NUC2                1H
PCPD2            90.00 usec
PL2               4.00 dB
PL12             20.80 dB
PL13             23.80 dB
SFO2       400.1319000 MHz

F2 - Processing parameters
SI               32768
SF         100.6127816 MHz
WDW                 EM
SSB                  0
LB                1.00 Hz
GB                   0
PC                1.40
```

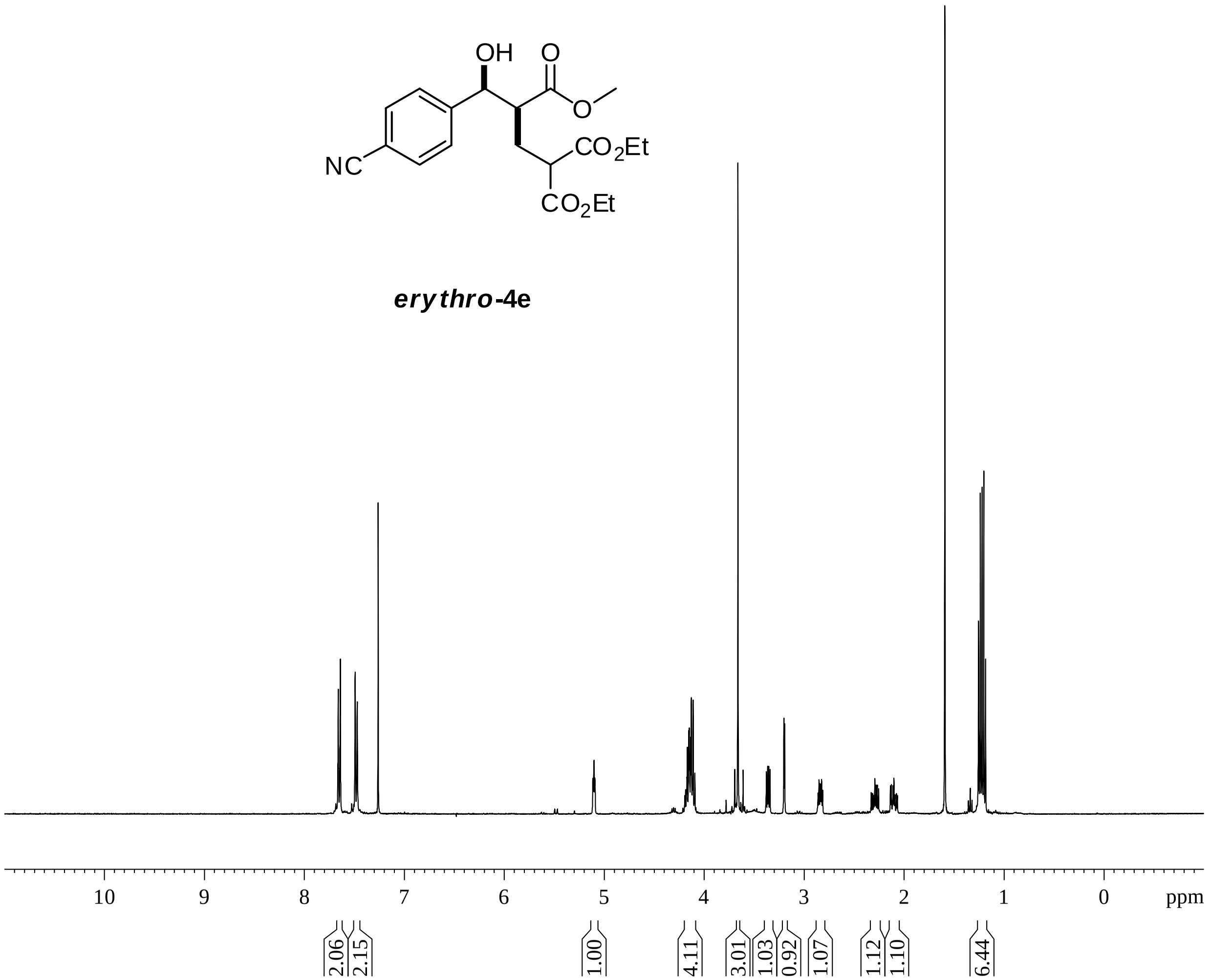

erythro-4e

```
Current Data Parameters
NAME             ncy130
EXPNO                 5
PROCNO                1

F2 - Acquisition Parameters
Date_          20090710
Time              20.25
INSTRUM           spect
PROBHD   5 mm BBO BB-1H
PULPROG            zg30
TD                16384
SOLVENT           CDCl3
NS                   16
DS                    0
SWH            5995.204 Hz
FIDRES         0.365918 Hz
AQ            1.3664756 sec
RG                228.1
DW               83.400 usec
DE                 6.50 usec
TE                299.2 K
D1           1.50000000 sec
MCREST       0.00000000 sec
MCWRK        0.01500000 sec

======== CHANNEL f1 ========
NUC1                 1H
P1                10.00 usec
PL1                0.00 dB
SFO1        400.1326008 MHz

F2 - Processing parameters
SI                16384
SF          400.1300088 MHz
WDW                  EM
SSB                   0
LB                 0.10 Hz
GB                    0
PC                 1.00

1D NMR plot parameters
CX                20.00 cm
CY                12.87 cm
F1P              10.500 ppm
F1              4201.37 Hz
F2P              -0.500 ppm
F2              -200.06 Hz
PPMCM           0.55000 ppm/cm
HZCM          220.07150 Hz/cm
```

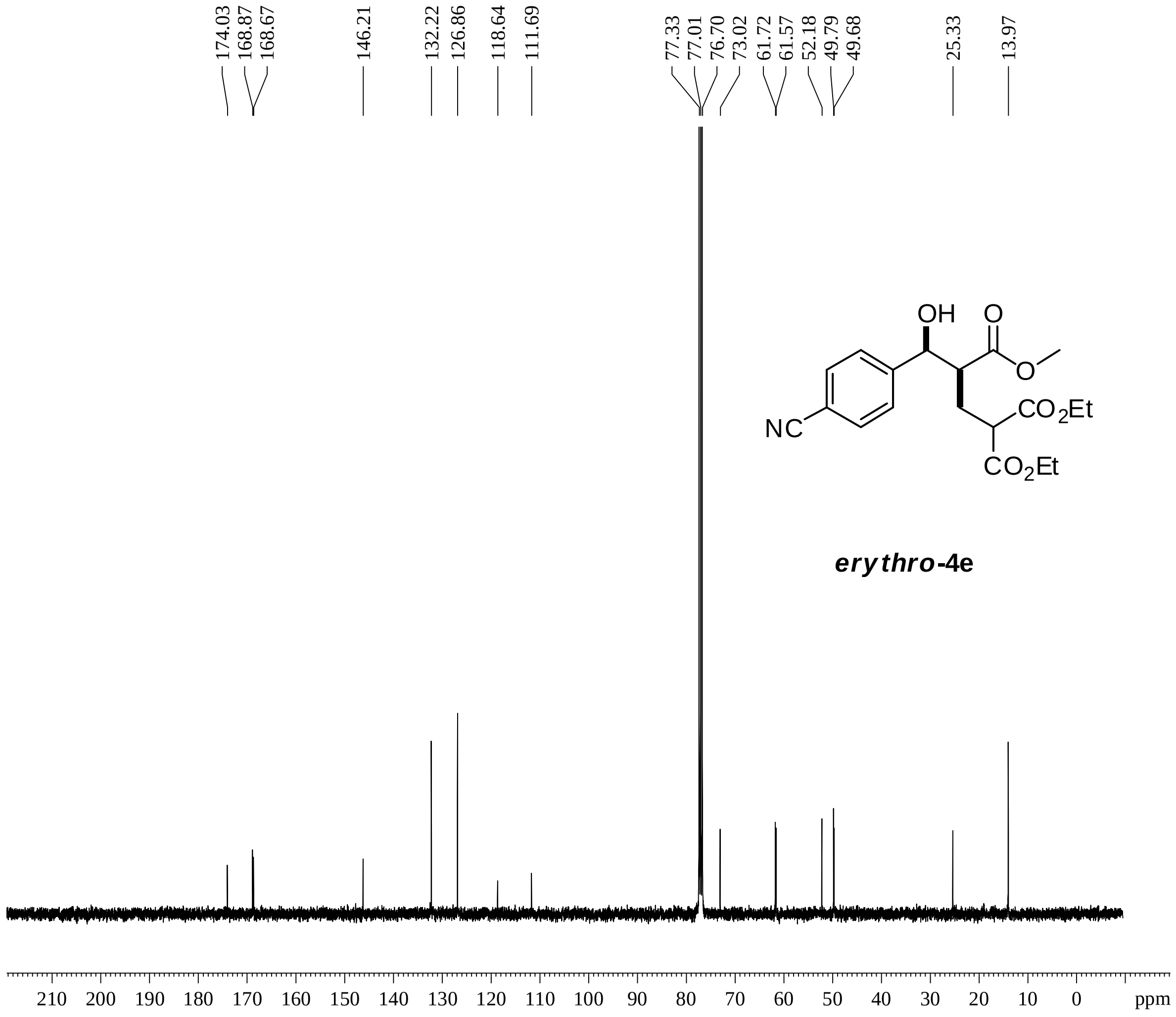

***erythro*-4e**

```
Current Data Parameters
NAME             ncy130
EXPNO                 8
PROCNO                1

F2 - Acquisition Parameters
Date_          20090730
Time              18.01
INSTRUM           spect
PROBHD   5 mm BBO BB-1H
PULPROG         zgpg30
TD                65536
SOLVENT           CDCl3
NS                 1023
DS                    0
SWH          25062.656 Hz
FIDRES        0.382426 Hz
AQ           1.3074932 sec
RG                 4096
DW               19.950 usec
DE                 6.50 usec
TE                298.4 K
D1           2.00000000 sec
d11          0.03000000 sec
DELTA        1.89999998 sec
MCREST       0.00000000 sec
MCWRK        0.01500000 sec

======== CHANNEL f1 ========
NUC1                13C
P1                 8.90 usec
PL1                5.00 dB
SFO1        100.6243390 MHz

======== CHANNEL f2 ========
CPDPRG2         waltz16
NUC2                 1H
PCPD2            100.00 usec
PL2                0.00 dB
PL12              19.10 dB
PL13              22.10 dB
SFO2        400.1319000 MHz

F2 - Processing parameters
SI                32768
SF          100.6127690 MHz
WDW                  EM
SSB                   0
LB                 1.00 Hz
GB                    0
PC                 1.40

1D NMR plot parameters
CX                20.00 cm
CY                34.78 cm
F1P             220.000 ppm
F1             22134.81 Hz
```

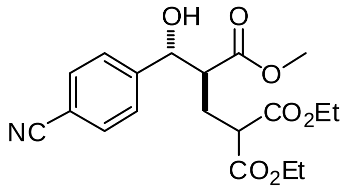

***threo*-4e**

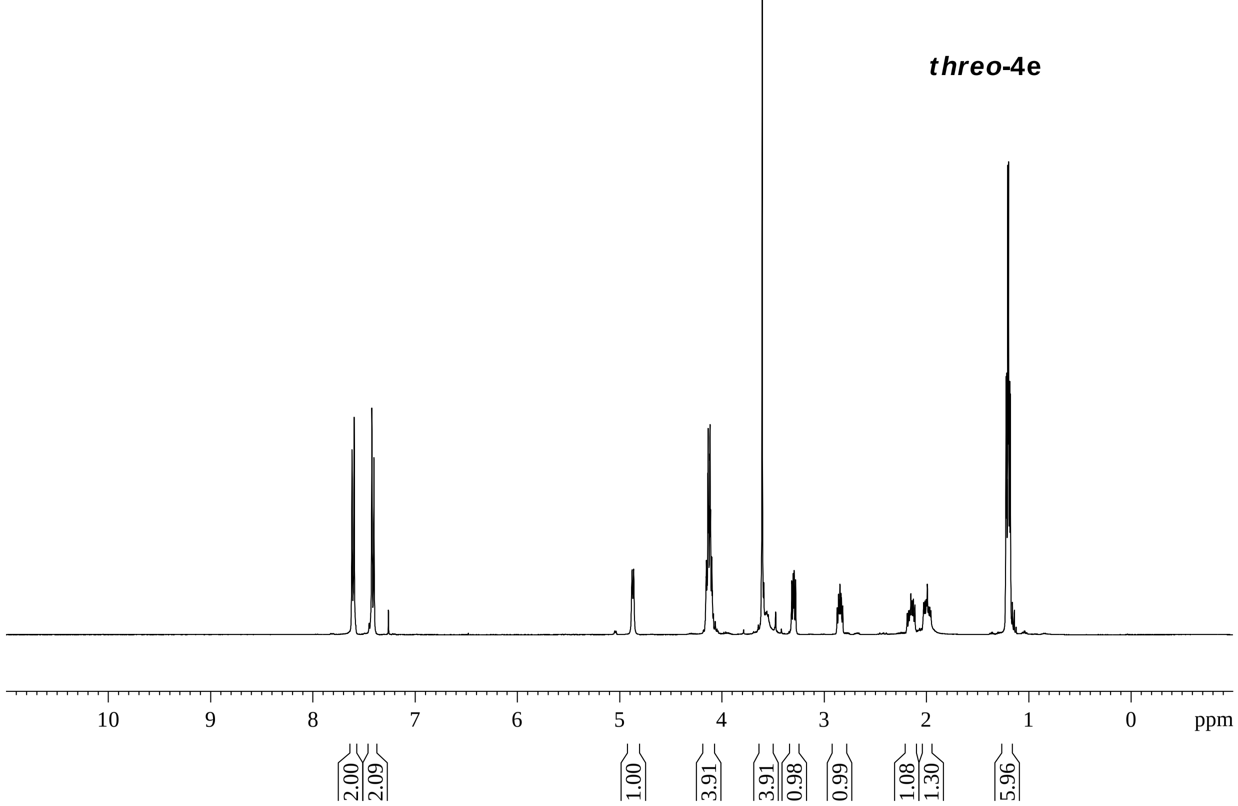


```
Current Data Parameters
NAME              ncy130
EXPNO                  3
PROCNO                 1

F2 - Acquisition Parameters
Date_           20090710
Time               20.12
INSTRUM            spect
PROBHD   5 mm BBO BB-1H
PULPROG             zg30
TD                 16384
SOLVENT            CDCl3
NS                    16
DS                     0
SWH             5995.204 Hz
FIDRES          0.365918 Hz
AQ             1.3664756 sec
RG                  35.9
DW                83.400 usec
DE                  6.50 usec
TE                 299.1 K
D1            1.50000000 sec
MCREST        0.00000000 sec
MCWRK         0.01500000 sec

======== CHANNEL f1 ========
NUC1                  1H
P1                 10.00 usec
PL1                 0.00 dB
SFO1         400.1326008 MHz

F2 - Processing parameters
SI                 16384
SF           400.1300088 MHz
WDW                   EM
SSB                    0
LB                  0.10 Hz
GB                     0
PC                  1.00

1D NMR plot parameters
CX                 20.00 cm
CY                 10.46 cm
F1P               10.500 ppm
F1               4201.37 Hz
F2P               -0.500 ppm
F2               -200.06 Hz
PPMCM            0.55000 ppm/cm
HZCM           220.07150 Hz/cm
```

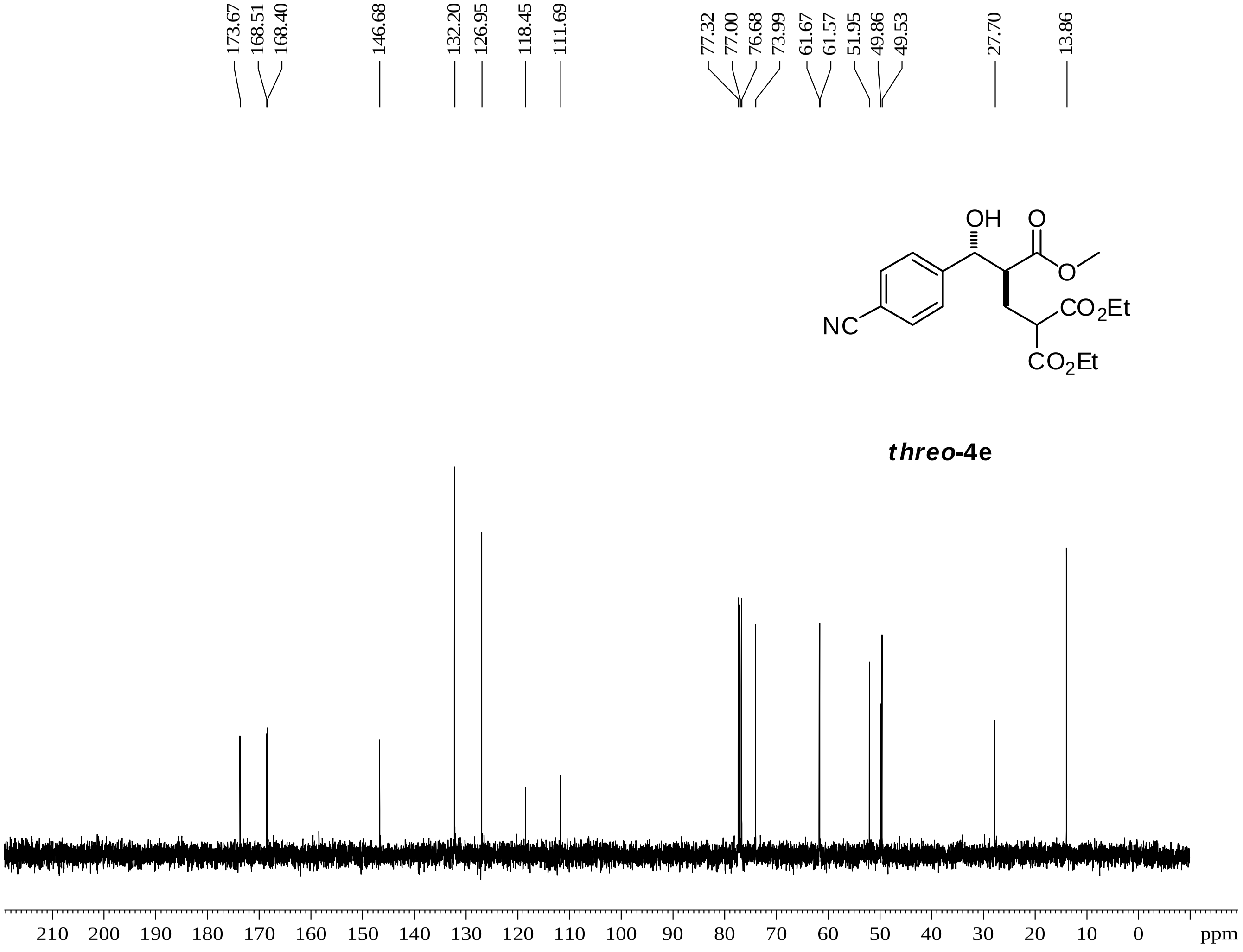

threo-4e

```
Current Data Parameters
NAME                 ncy130
EXPNO                     4
PROCNO                    1

F2 - Acquisition Parameters
Date_          20090710
Time              20.17
INSTRUM           spect
PROBHD   5 mm BBO BB-1H
PULPROG         zgpg30
TD                65536
SOLVENT           CDCl3
NS                   12
DS                    0
SWH          25125.629 Hz
FIDRES        0.383387 Hz
AQ           1.3042164 sec
RG                 4096
DW               19.900 usec
DE                 6.50 usec
TE                299.7 K
D1           2.00000000 sec
d11          0.03000000 sec
DELTA        1.89999998 sec
MCREST       0.00000000 sec
MCWRK        0.01500000 sec

======== CHANNEL f1 ========
NUC1                13C
P1                 8.90 usec
PL1                5.00 dB
SFO1        100.6243390 MHz

======== CHANNEL f2 ========
CPDPRG2         waltz16
NUC2                 1H
PCPD2            100.00 usec
PL2                0.00 dB
PL12              19.10 dB
PL13              22.10 dB
SFO2        400.1319000 MHz

F2 - Processing parameters
SI                32768
SF          100.6127794 MHz
WDW                  EM
SSB                   0
LB                 1.00 Hz
GB                    0
PC                 1.40

1D NMR plot parameters
CX                20.00 cm
CY                11.82 cm
F1P             220.000 ppm
F1             22134.81 Hz
```

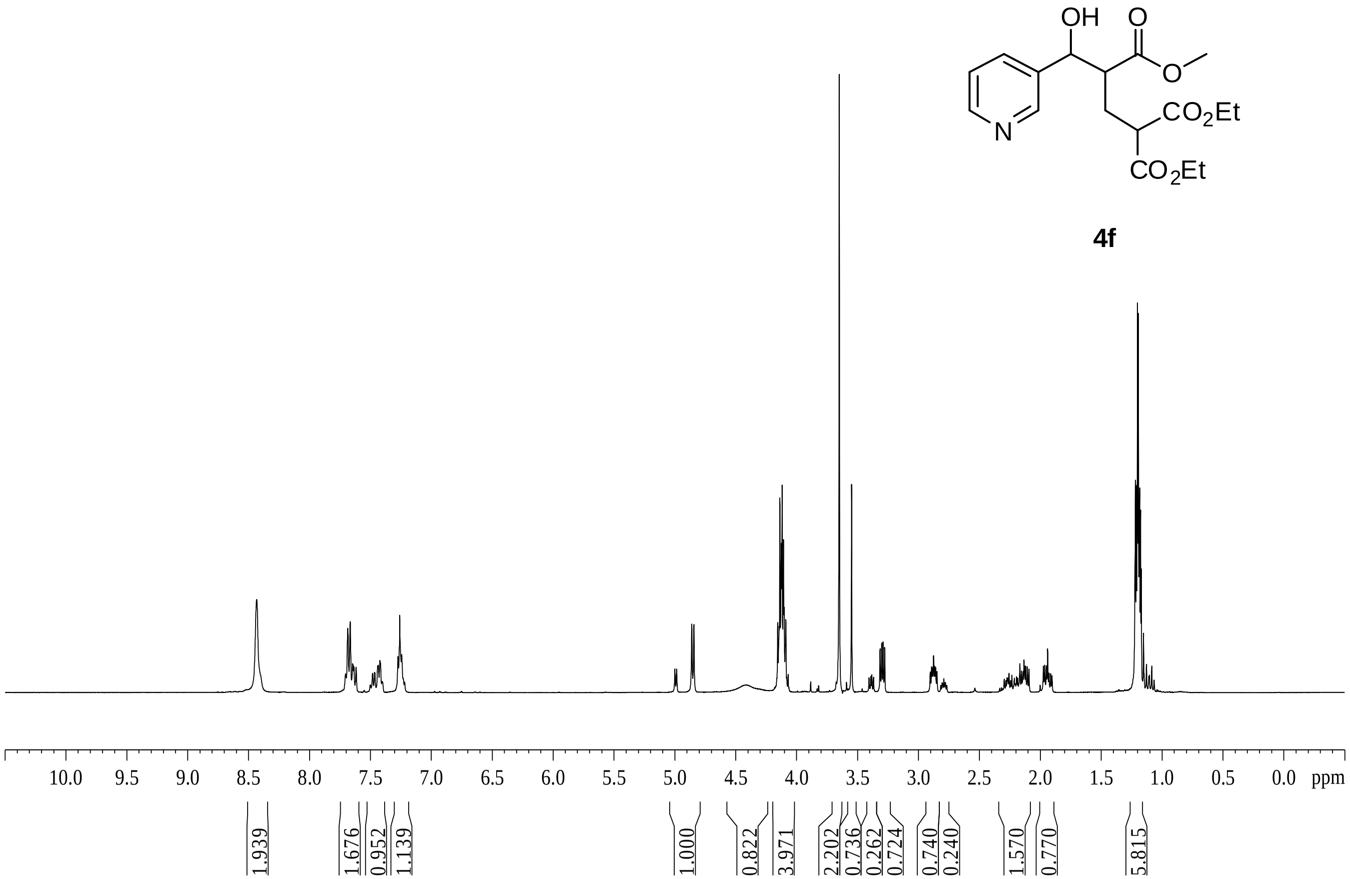

4f

```
Current Data Parameters
NAME              ncy250
EXPNO                 12
PROCNO                 1

F2 - Acquisition Parameters
Date_           20100503
Time               16.26
INSTRUM            spect
PROBHD   5 mm BBO BB-1H
PULPROG             zg30
TD                 16384
SOLVENT            CDCl3
NS                    16
DS                     0
SWH             5995.204 Hz
FIDRES          0.365918 Hz
AQ             1.3664756 sec
RG                    64
DW                83.400 usec
DE                  8.00 usec
TE                 297.6 K
D1            2.00000000 sec
MCREST        0.00000000 sec
MCWRK         0.01500000 sec

======== CHANNEL f1 ========
NUC1                  1H
P1                 10.00 usec
PL1                 0.00 dB
SFO1         400.1326008 MHz

F2 - Processing parameters
SI                 16384
SF           400.1300099 MHz
WDW                   EM
SSB                    0
LB                  0.10 Hz
GB                     0
PC                  1.00
```

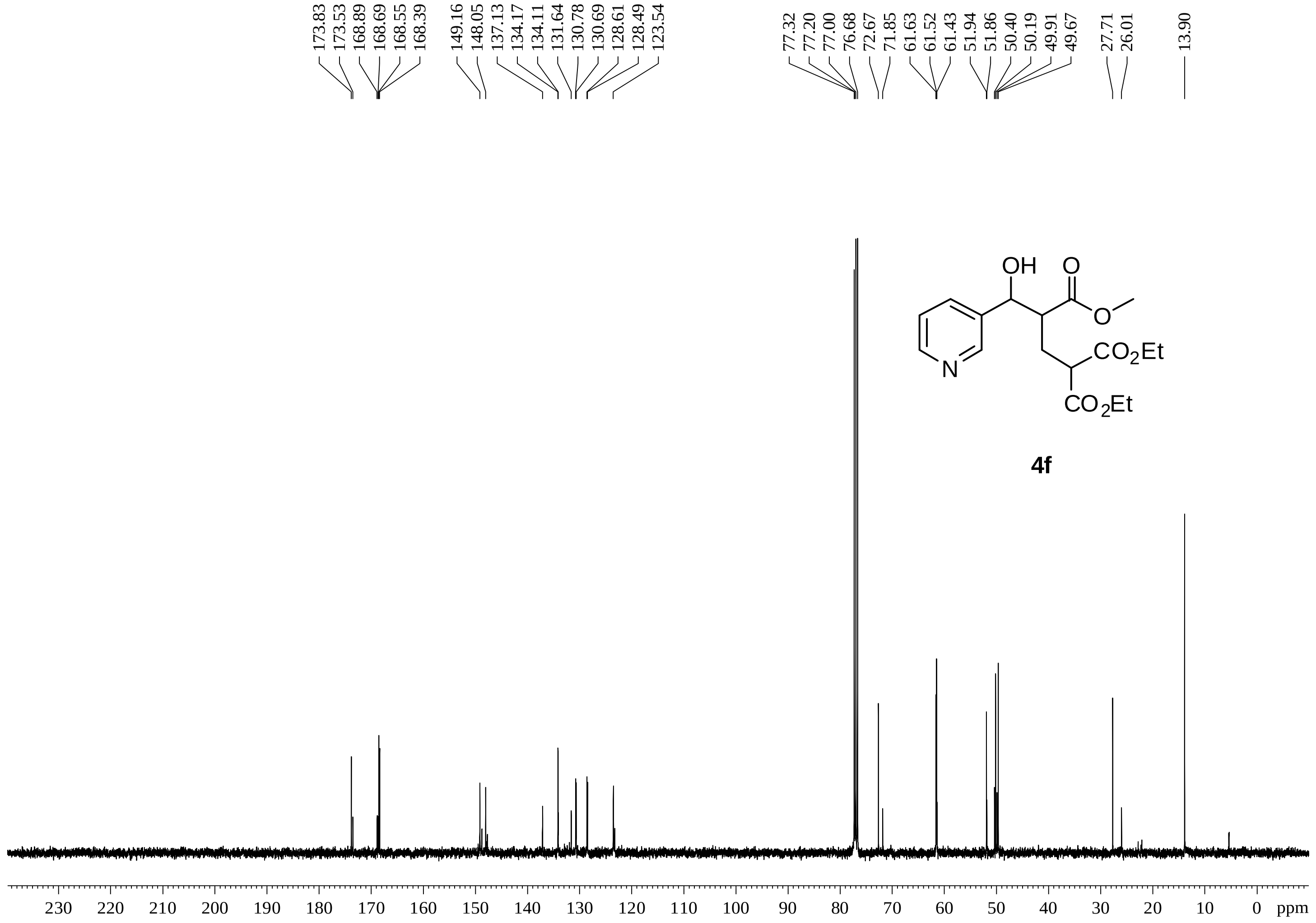

4f

```
Current Data Parameters
NAME              ncy250
EXPNO                 13
PROCNO                 1

F2 - Acquisition Parameters
Date_           20100503
Time               16.27
INSTRUM            spect
PROBHD   5 mm BBO BB-1H
PULPROG          zgpg30
TD                 65536
SOLVENT            CDCl3
NS                   436
DS                     0
SWH          25125.629 Hz
FIDRES        0.383387 Hz
AQ           1.3042164 sec
RG                  4096
DW              19.900 usec
DE                8.00 usec
TE               297.8 K
D1          2.00000000 sec
d11         0.03000000 sec
DELTA       1.89999998 sec
MCREST      0.00000000 sec
MCWRK       0.01500000 sec

======== CHANNEL f1 ========
NUC1                 13C
P1                  8.90 usec
PL1                 5.00 dB
SFO1        100.6243390 MHz

======== CHANNEL f2 ========
CPDPRG2           waltz16
NUC2                  1H
PCPD2              90.00 usec
PL2                 0.00 dB
PL12               19.10 dB
PL13               22.10 dB
SFO2        400.1319000 MHz

F2 - Processing parameters
SI                 32768
SF          100.6127756 MHz
WDW                   EM
SSB                    0
LB                  1.00 Hz
GB                     0
PC                  1.40
```

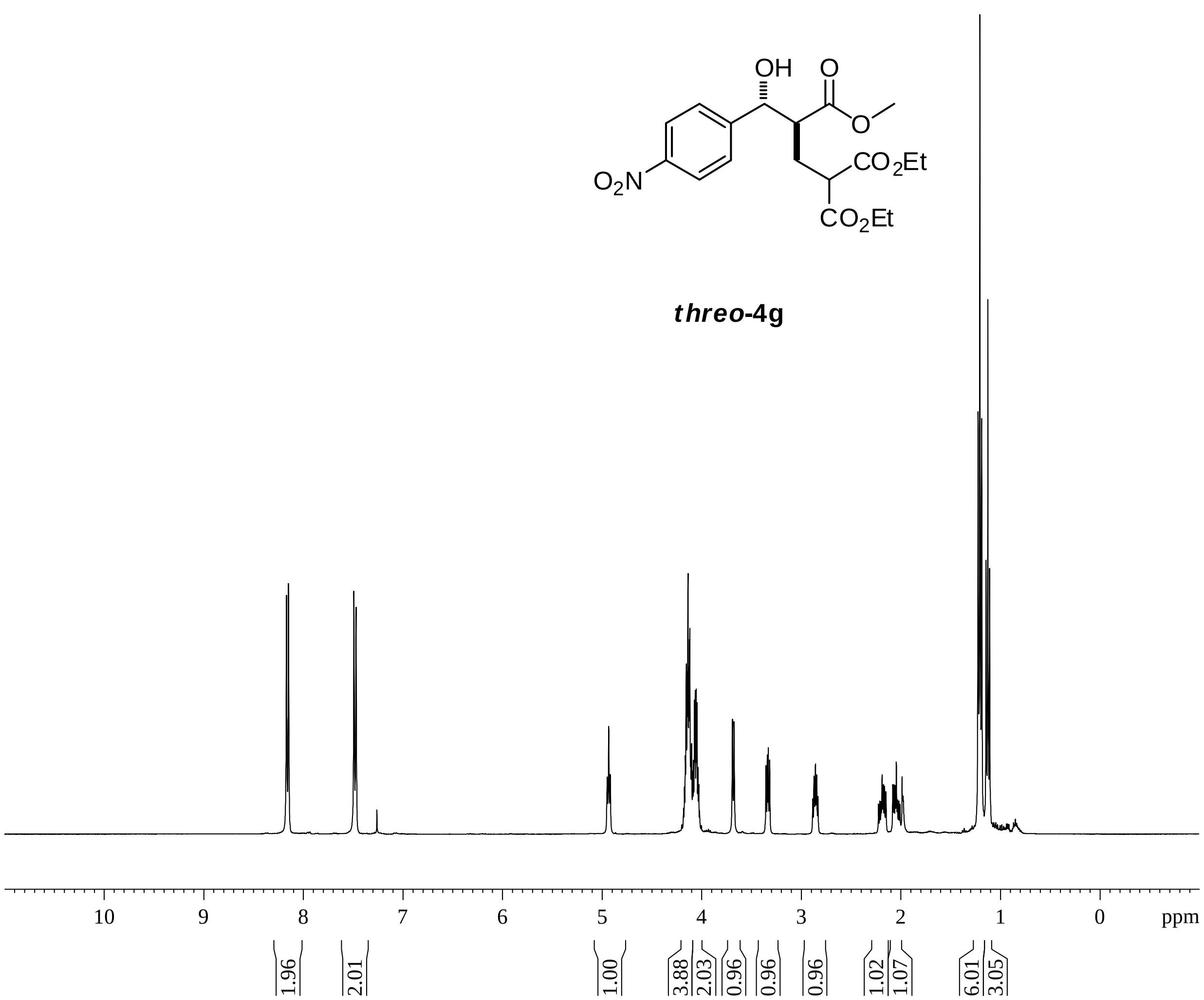


```
Current Data Parameters
NAME              ncy138
EXPNO                  5
PROCNO                 1

F2 - Acquisition Parameters
Date_           20090731
Time               20.26
INSTRUM            spect
PROBHD   5 mm BBO BB-1H
PULPROG             zg30
TD                 16384
SOLVENT            CDCl3
NS                    16
DS                     0
SWH             5995.204 Hz
FIDRES          0.365918 Hz
AQ             1.3664756 sec
RG                    32
DW                83.400 usec
DE                  6.50 usec
TE                 298.8 K
D1            1.50000000 sec
MCREST        0.00000000 sec
MCWRK         0.01500000 sec

======== CHANNEL f1 ========
NUC1                  1H
P1                 10.00 usec
PL1                 0.00 dB
SFO1         400.1326008 MHz

F2 - Processing parameters
SI                 16384
SF           400.1300084 MHz
WDW                   EM
SSB                    0
LB                  0.10 Hz
GB                     0
PC                  1.00

1D NMR plot parameters
CX                 20.00 cm
CY                 10.43 cm
F1P               10.500 ppm
F1               4201.37 Hz
F2P               -0.500 ppm
F2               -200.06 Hz
PPMCM            0.55000 ppm/cm
HZCM           220.07150 Hz/cm
```

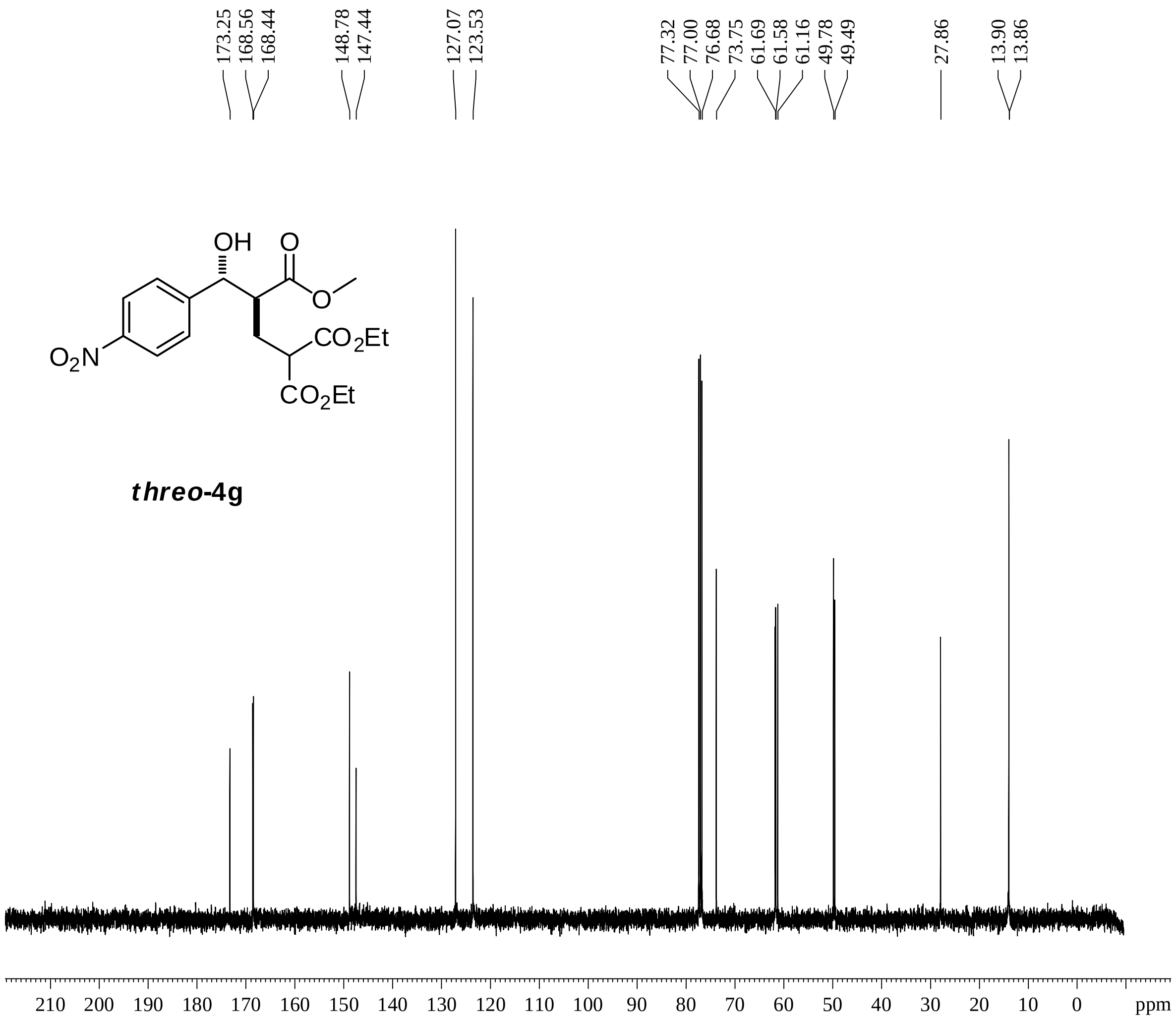

***threo*-4g**

```
Current Data Parameters
NAME              ncy138
EXPNO                  6
PROCNO                 1

F2 - Acquisition Parameters
Date_           20090731
Time               20.29
INSTRUM            spect
PROBHD   5 mm BBO BB-1H
PULPROG          zgpg30
TD                 65536
SOLVENT            CDCl3
NS                    59
DS                     0
SWH            25062.656 Hz
FIDRES          0.382426 Hz
AQ             1.3074932 sec
RG                  4096
DW                19.950 usec
DE                  6.50 usec
TE                 299.2 K
D1            2.00000000 sec
d11           0.03000000 sec
DELTA         1.89999998 sec
MCREST        0.00000000 sec
MCWRK         0.01500000 sec

======== CHANNEL f1 ========
NUC1                 13C
P1                  8.90 usec
PL1                 5.00 dB
SFO1        100.6243390 MHz

======== CHANNEL f2 ========
CPDPRG2          waltz16
NUC2                  1H
PCPD2             100.00 usec
PL2                 0.00 dB
PL12               19.10 dB
PL13               22.10 dB
SFO2        400.1319000 MHz

F2 - Processing parameters
SI                 32768
SF          100.6127783 MHz
WDW                   EM
SSB                    0
LB                  1.00 Hz
GB                     0
PC                  1.40

1D NMR plot parameters
CX                 20.00 cm
CY                 11.02 cm
F1P              220.000 ppm
F1              22134.81 Hz
```

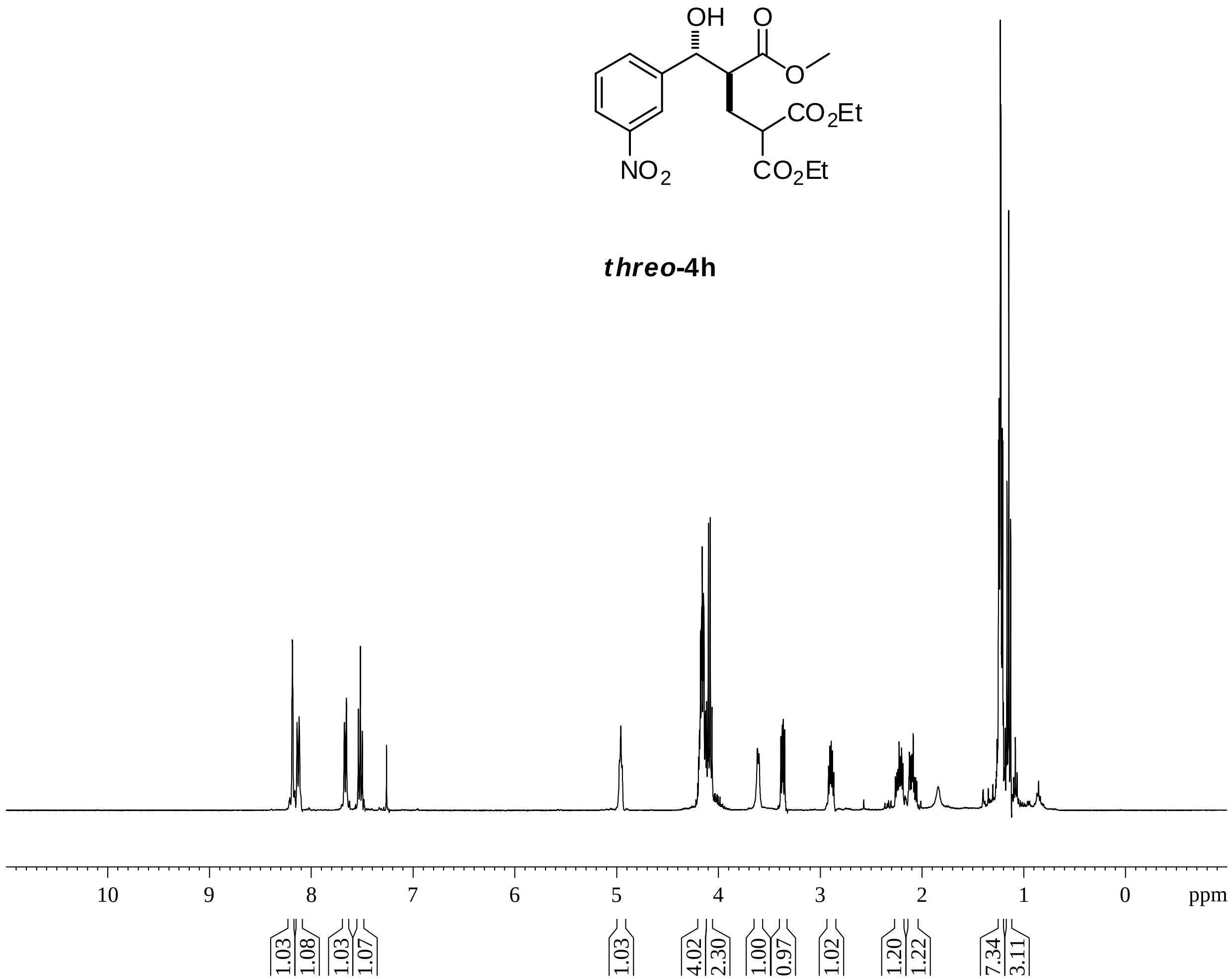

***threo*-4h**

```
Current Data Parameters
NAME              ncy116
EXPNO                  3
PROCNO                 1

F2 - Acquisition Parameters
Date_           20090602
Time               21.40
INSTRUM            spect
PROBHD   5 mm BBO BB-1H
PULPROG             zg30
TD                 16384
SOLVENT            CDCl3
NS                     1
DS                     0
SWH             5995.204 Hz
FIDRES          0.365918 Hz
AQ             1.3664756 sec
RG                  50.8
DW                83.400 usec
DE                  6.50 usec
TE                 299.1 K
D1            1.50000000 sec
MCREST        0.00000000 sec
MCWRK         0.01500000 sec

======== CHANNEL f1 ========
NUC1                  1H
P1                 10.00 usec
PL1                 0.00 dB
SFO1         400.1326008 MHz

F2 - Processing parameters
SI                 16384
SF           400.1300091 MHz
WDW                   EM
SSB                    0
LB                  0.10 Hz
GB                     0
PC                  1.00
```

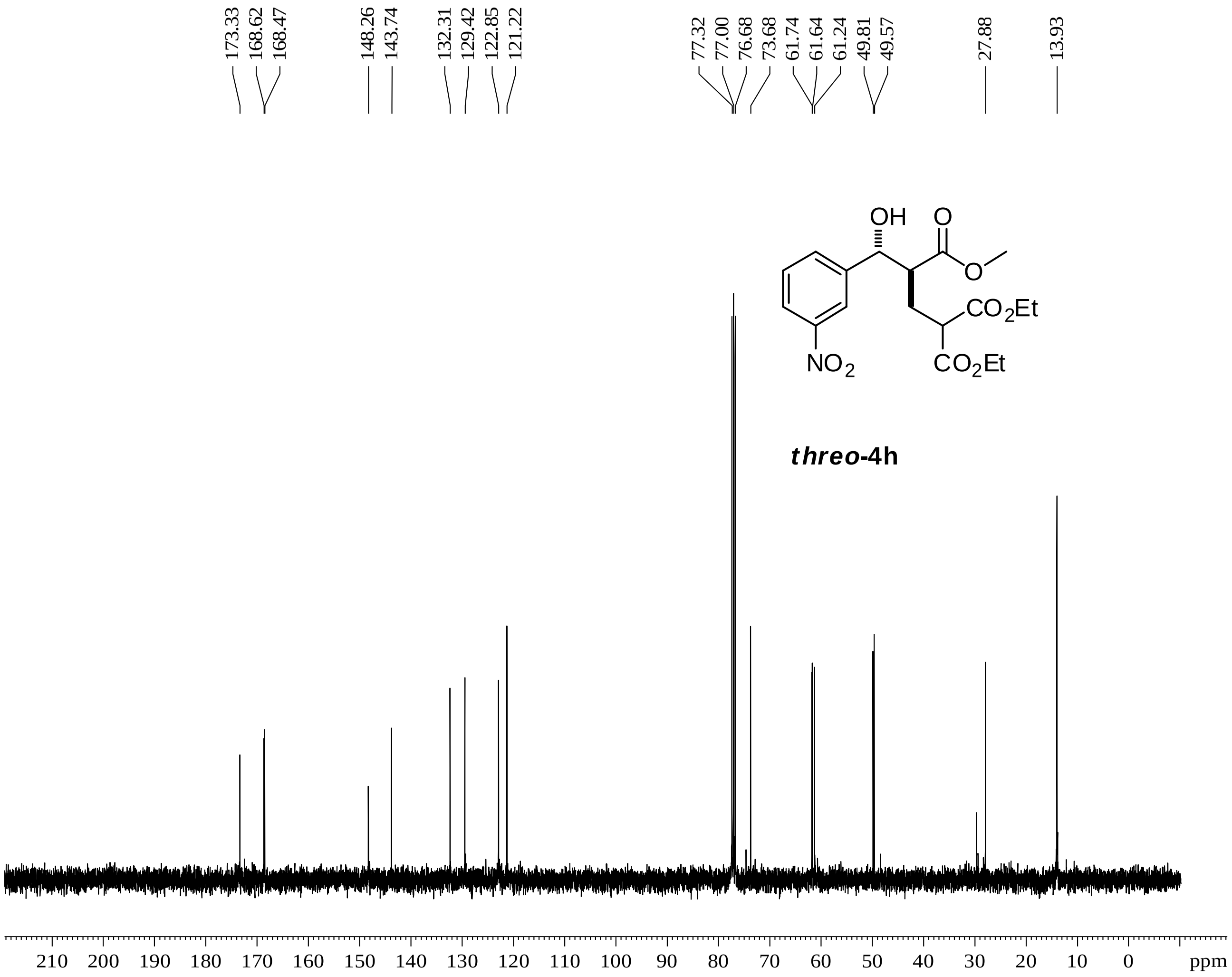


```
Current Data Parameters
NAME              ncy116
EXPNO                  4
PROCNO                 1

F2 - Acquisition Parameters
Date_           20090602
Time               21.42
INSTRUM            spect
PROBHD   5 mm BBO BB-1H
PULPROG          zgpg30
TD                 65536
SOLVENT            CDCl3
NS                    51
DS                     0
SWH            25125.629 Hz
FIDRES          0.383387 Hz
AQ             1.3042164 sec
RG                  4096
DW                19.900 usec
DE                  6.50 usec
TE                 299.1 K
D1            2.00000000 sec
d11           0.03000000 sec
DELTA         1.89999998 sec
MCREST        0.00000000 sec
MCWRK         0.01500000 sec

======== CHANNEL f1 ========
NUC1                 13C
P1                  8.90 usec
PL1                 5.00 dB
SFO1         100.6242995 MHz

======== CHANNEL f2 ========
CPDPRG2          waltz16
NUC2                  1H
PCPD2              90.00 usec
PL2                 0.00 dB
PL12               19.10 dB
PL13               22.10 dB
SFO2         400.1319000 MHz

F2 - Processing parameters
SI                 32768
SF           100.6127752 MHz
WDW                   EM
SSB                    0
LB                  1.00 Hz
GB                     0
PC                  1.40

1D NMR plot parameters
CX                 20.00 cm
CY                 11.02 cm
F1P              220.000 ppm
F1              22134.81 Hz
```

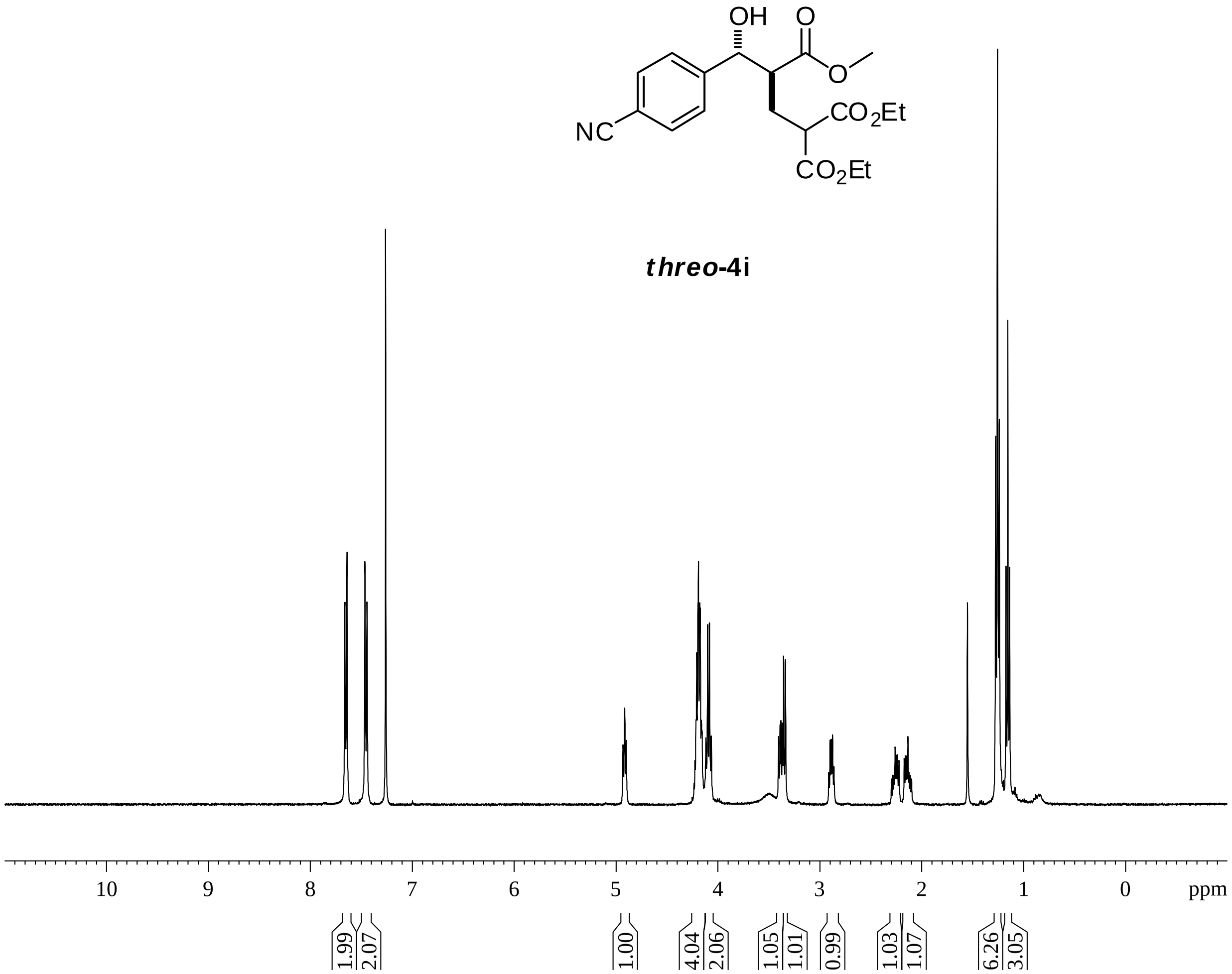

***threo*-4i**

```
Current Data Parameters
NAME             ncy140
EXPNO                 5
PROCNO                1

F2 - Acquisition Parameters
Date_          20100529
Time              15.49
INSTRUM           spect
PROBHD   5 mm BBO BB-1H
PULPROG            zg30
TD                16384
SOLVENT           CDCl3
NS                   16
DS                    0
SWH            6009.615 Hz
FIDRES         0.366798 Hz
AQ            1.3631988 sec
RG                406.4
DW               83.200 usec
DE                 6.50 usec
TE                299.6 K
D1           1.50000000 sec
MCREST       0.00000000 sec
MCWRK        0.01500000 sec

======== CHANNEL f1 ========
NUC1                 1H
P1                14.50 usec
PL1                5.20 dB
SFO1        400.1326008 MHz

F2 - Processing parameters
SI                16384
SF          400.1300095 MHz
WDW                  EM
SSB                   0
LB                 0.10 Hz
GB                    0
PC                 1.00

1D NMR plot parameters
CX                20.00 cm
CY                10.44 cm
F1P              10.500 ppm
F1              4201.37 Hz
F2P              -0.500 ppm
F2              -200.06 Hz
PPMCM           0.55000 ppm/cm
HZCM          220.07150 Hz/cm
```

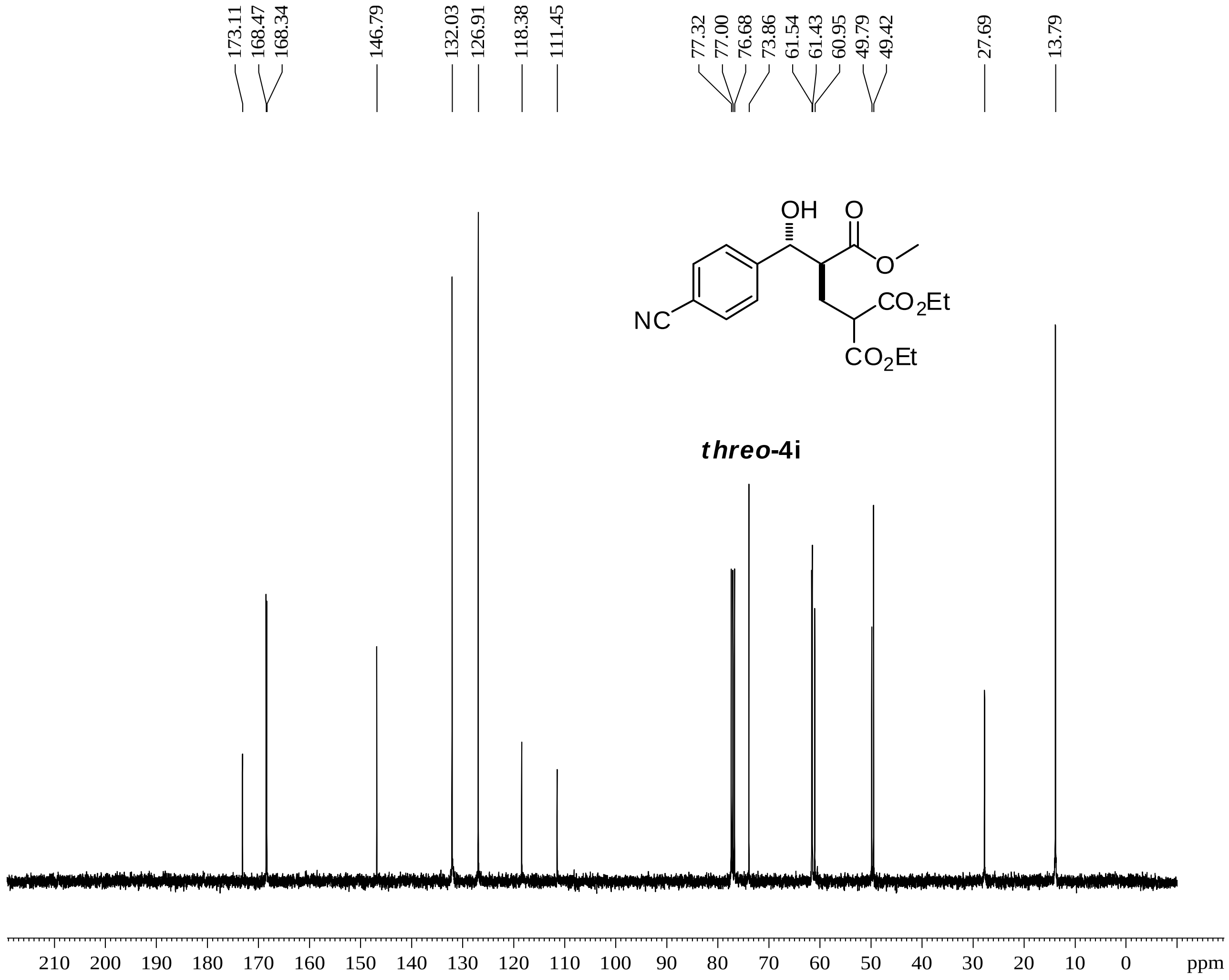

threo-4i

```
Current Data Parameters
NAME              ncy140
EXPNO                  6
PROCNO                 1

F2 - Acquisition Parameters
Date_           20100529
Time               18.07
INSTRUM            spect
PROBHD   5 mm BBO BB-1H
PULPROG          zgpg30
TD                 65536
SOLVENT            CDCl3
NS                   153
DS                     0
SWH            25062.656 Hz
FIDRES          0.382426 Hz
AQ             1.3074932 sec
RG                  4096
DW                19.950 usec
DE                  6.50 usec
TE                 300.0 K
D1            2.00000000 sec
d11           0.03000000 sec
DELTA         1.89999998 sec
MCREST        0.00000000 sec
MCWRK         0.01500000 sec

======== CHANNEL f1 ========
NUC1                 13C
P1                 10.00 usec
PL1                 5.00 dB
SFO1         100.6242995 MHz

======== CHANNEL f2 ========
CPDPRG2          waltz16
NUC2                  1H
PCPD2              90.00 usec
PL2                 4.00 dB
PL12               20.80 dB
PL13               23.80 dB
SFO2         400.1319000 MHz

F2 - Processing parameters
SI                 32768
SF           100.6127862 MHz
WDW                   EM
SSB                    0
LB                  1.00 Hz
GB                     0
PC                  1.40

1D NMR plot parameters
CX                 20.00 cm
CY                 10.96 cm
F1P              220.000 ppm
F1              22134.81 Hz
```

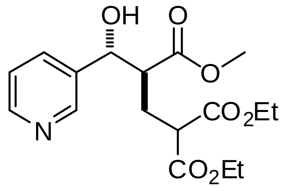

threo-4j

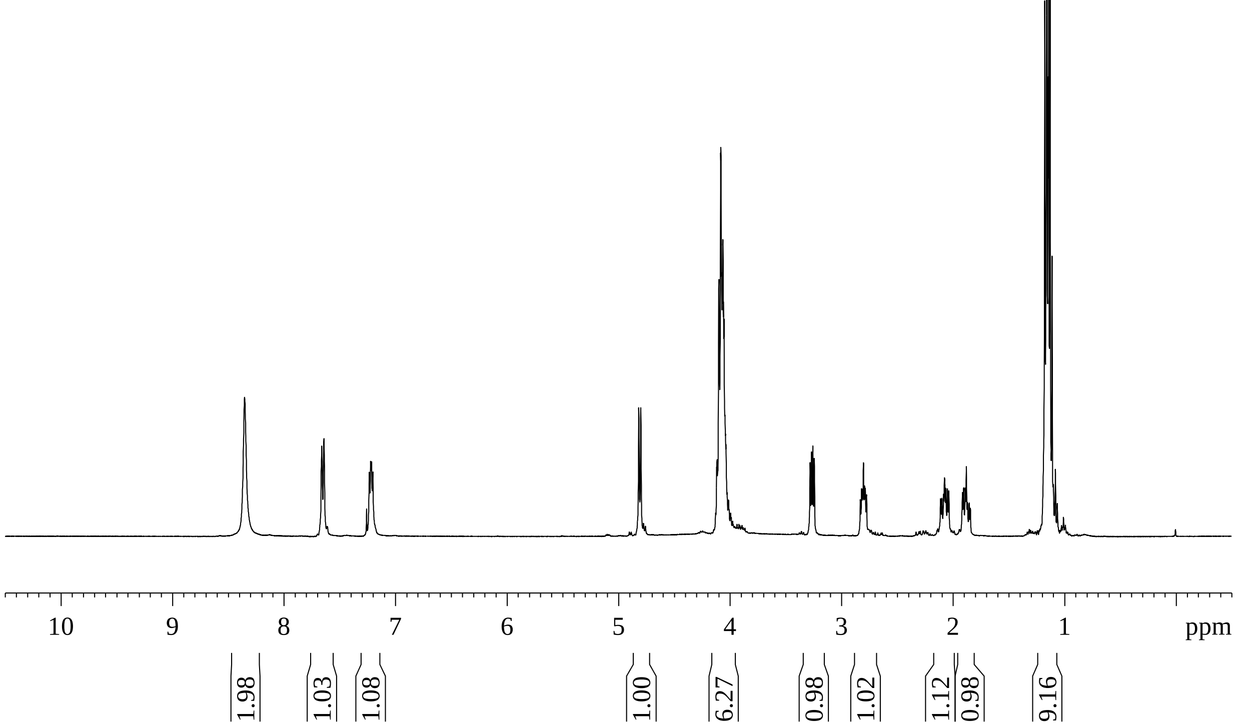


```
Current Data Parameters
NAME             ncy153
EXPNO                 7
PROCNO                1

F2 - Acquisition Parameters
Date_          20090919
Time              18.27
INSTRUM           spect
PROBHD   5 mm BBO BB-1H
PULPROG            zg30
TD                16384
SOLVENT           CDCl3
NS                    1
DS                    0
SWH            5995.204 Hz
FIDRES         0.365918 Hz
AQ            1.3664756 sec
RG                 22.6
DW               83.400 usec
DE                 6.50 usec
TE                299.9 K
D1           1.50000000 sec
MCREST       0.00000000 sec
MCWRK        0.01500000 sec

======== CHANNEL f1 ========
NUC1                 1H
P1                10.00 usec
PL1                0.00 dB
SFO1        400.1326008 MHz

F2 - Processing parameters
SI                16384
SF          400.1300084 MHz
WDW                  EM
SSB                   0
LB                 0.10 Hz
GB                    0
PC                 1.00

1D NMR plot parameters
CX                20.00 cm
CY                 6.21 cm
F1P              10.500 ppm
F1              4201.37 Hz
F2P              -0.500 ppm
F2              -200.06 Hz
PPMCM           0.55000 ppm/cm
HZCM          220.07150 Hz/cm
```

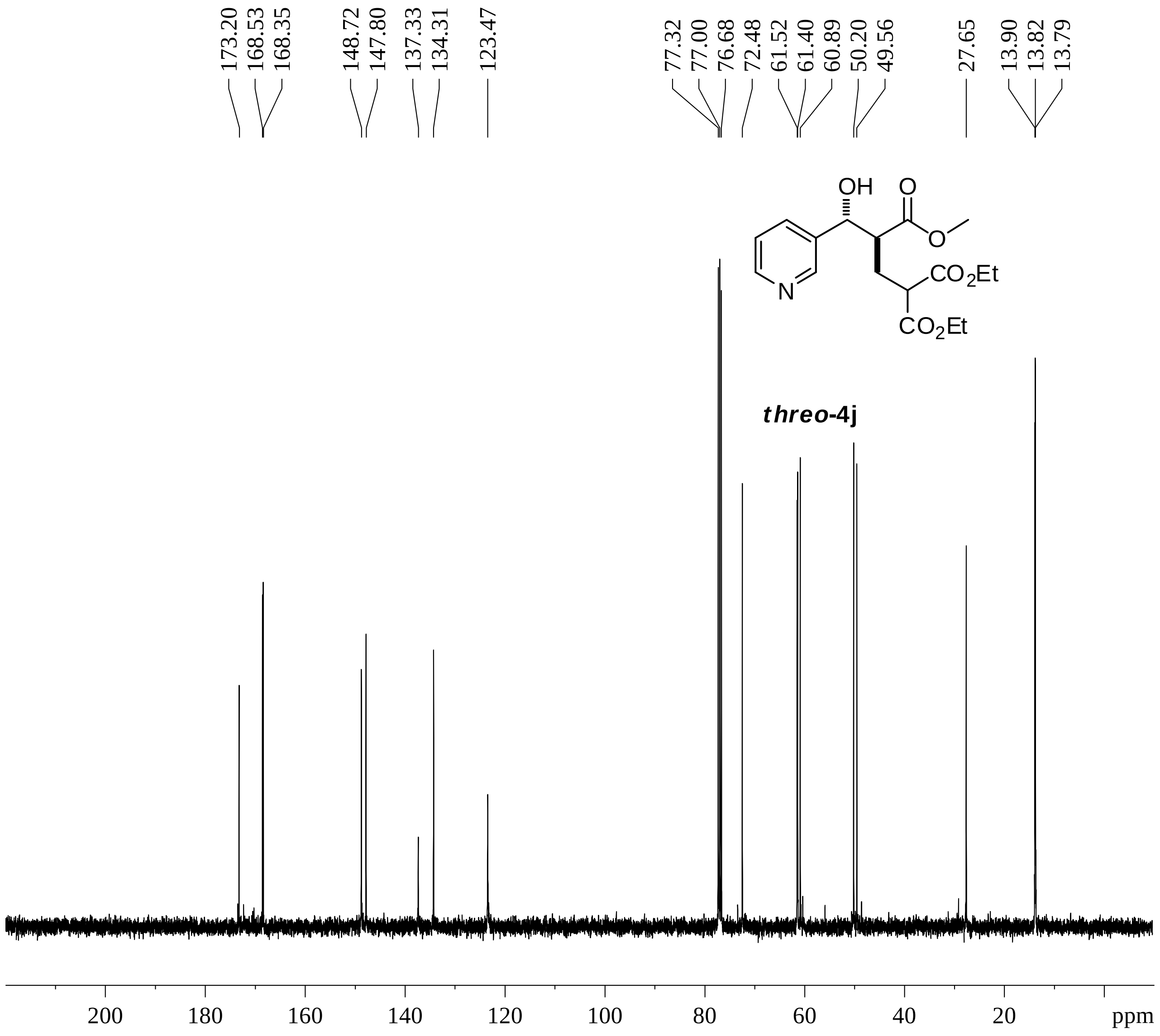


```
Current Data Parameters
NAME             ncy153
EXPNO                 8
PROCNO                1

F2 - Acquisition Parameters
Date_          20090919
Time              18.34
INSTRUM           spect
PROBHD   5 mm BBO BB-1H
PULPROG         zgpg30
TD                65536
SOLVENT           CDCl3
NS                  156
DS                    0
SWH           25062.656 Hz
FIDRES         0.382426 Hz
AQ            1.3074932 sec
RG                 2048
DW               19.950 usec
DE                 6.50 usec
TE                300.5 K
D1           2.00000000 sec
d11          0.03000000 sec
DELTA        1.89999998 sec
MCREST       0.00000000 sec
MCWRK        0.01500000 sec

======== CHANNEL f1 ========
NUC1                13C
P1                 8.90 usec
PL1                5.00 dB
SFO1        100.6243390 MHz

======== CHANNEL f2 ========
CPDPRG2         waltz16
NUC2                 1H
PCPD2            100.00 usec
PL2                0.00 dB
PL12              19.10 dB
PL13              22.10 dB
SFO2        400.1319000 MHz

F2 - Processing parameters
SI                32768
SF          100.6127806 MHz
WDW                  EM
SSB                   0
LB                 1.00 Hz
GB                    0
PC                 1.40

1D NMR plot parameters
CX                20.00 cm
CY                11.04 cm
F1P             220.000 ppm
F1             22134.81 Hz
F2P             -10.000 ppm
F2             -1006.13 Hz
PPMCM          11.50000 ppm/cm
HZCM         1157.04688 Hz/cm
```

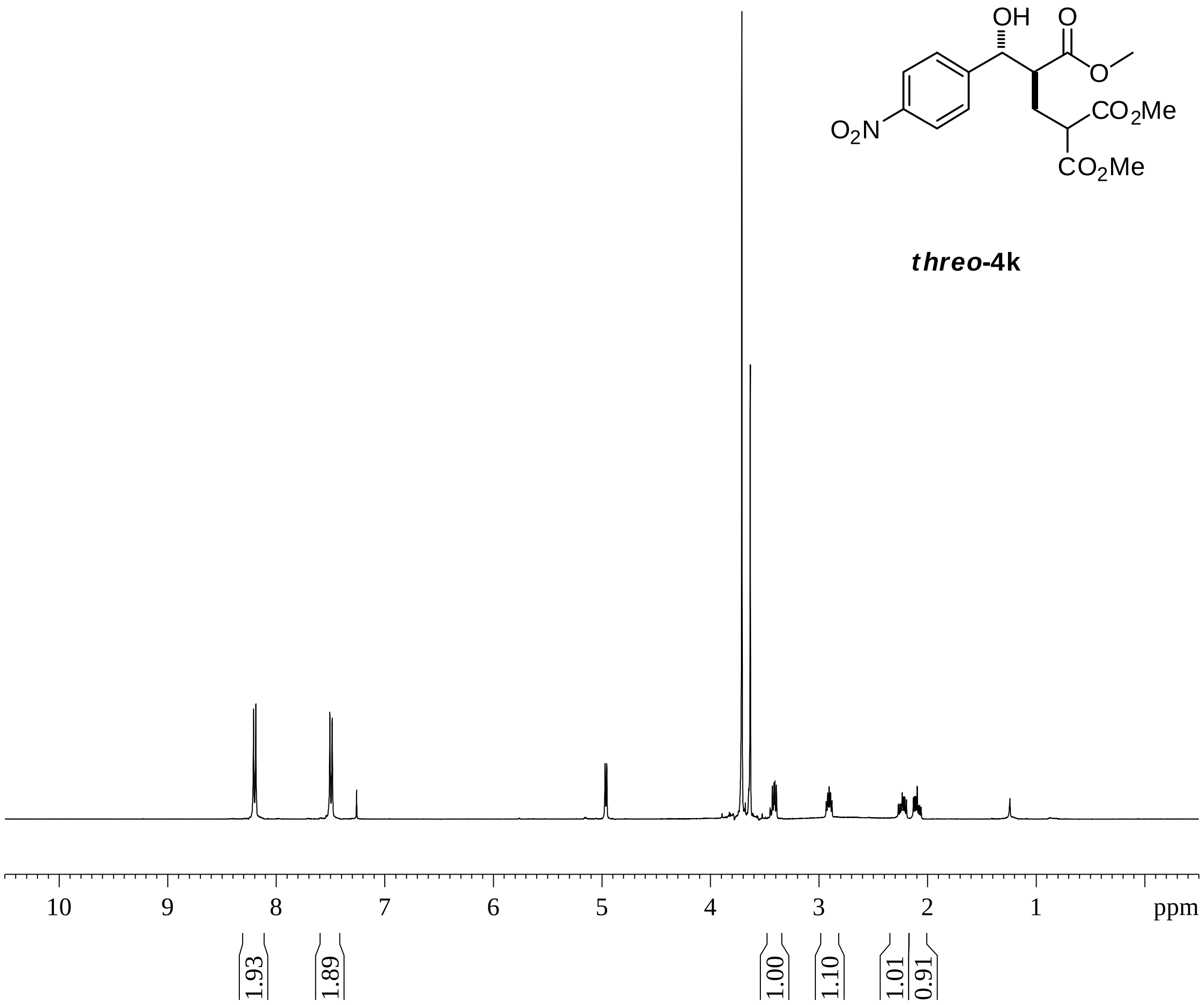


```
Current Data Parameters
NAME              ncy152
EXPNO                  5
PROCNO                 1

F2 - Acquisition Parameters
Date_           20090919
Time               18.44
INSTRUM            spect
PROBHD   5 mm BBO BB-1H
PULPROG             zg30
TD                 16384
SOLVENT            CDCl3
NS                     1
DS                     0
SWH             5995.204 Hz
FIDRES          0.365918 Hz
AQ             1.3664756 sec
RG                 101.6
DW                83.400 usec
DE                  6.50 usec
TE                 299.9 K
D1            1.50000000 sec
MCREST        0.00000000 sec
MCWRK         0.01500000 sec

======== CHANNEL f1 ========
NUC1                  1H
P1                 10.00 usec
PL1                 0.00 dB
SFO1        400.1326008 MHz

F2 - Processing parameters
SI                 16384
SF           400.1300088 MHz
WDW                   EM
SSB                    0
LB                  0.10 Hz
GB                     0
PC                  1.00

1D NMR plot parameters
CX                 20.00 cm
CY                 13.29 cm
F1P               10.500 ppm
F1               4201.37 Hz
F2P               -0.500 ppm
F2               -200.06 Hz
PPMCM            0.55000 ppm/cm
HZCM           220.07150 Hz/cm
```

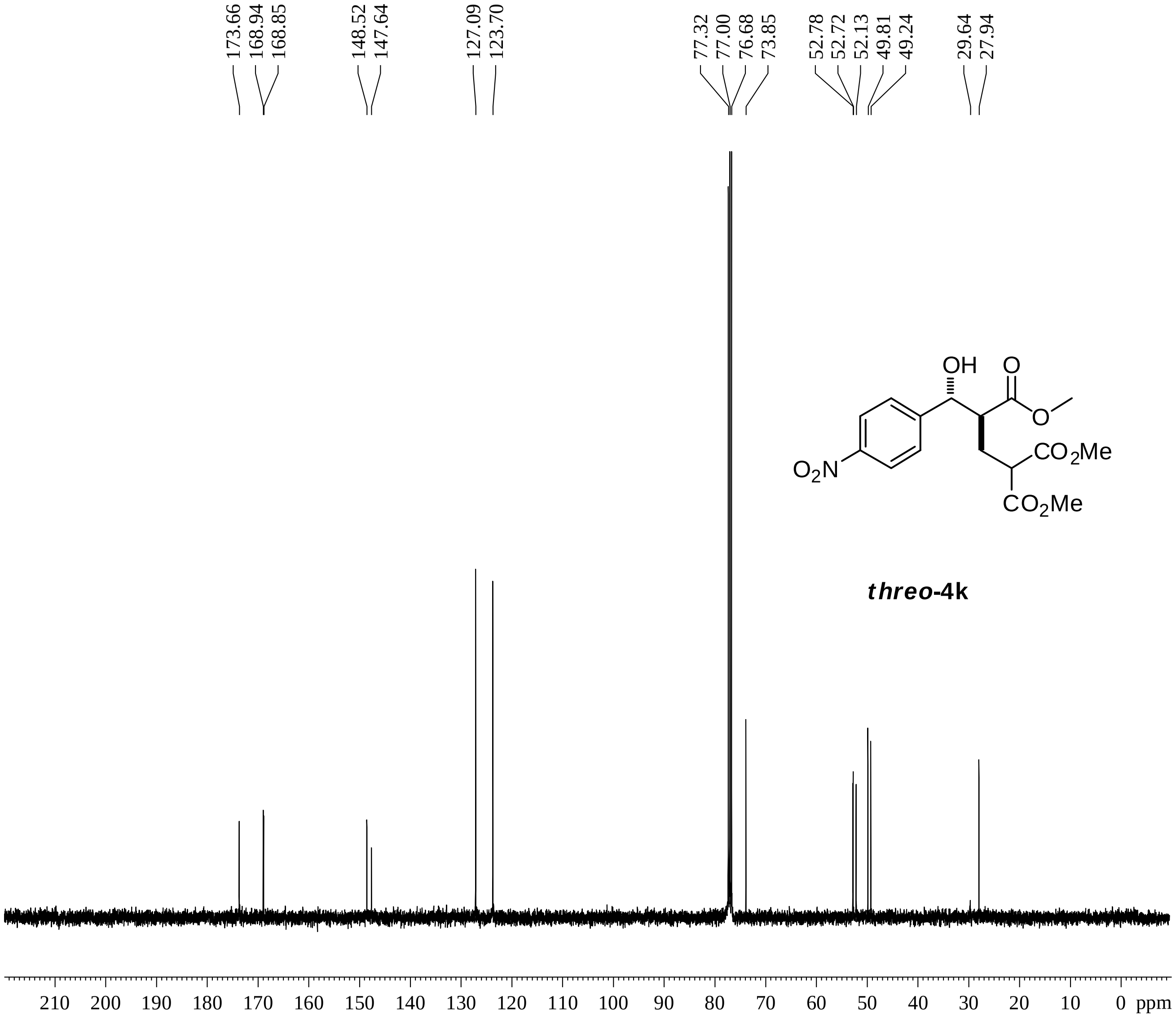

***threo*-4k**

```
Current Data Parameters
NAME             ncy152
EXPNO                 6
PROCNO                1

F2 - Acquisition Parameters
Date_          20090919
Time              18.49
INSTRUM           spect
PROBHD   5 mm BBO BB-1H
PULPROG         zgpg30
TD                65536
SOLVENT           CDCl3
NS                  187
DS                    0
SWH           25062.656 Hz
FIDRES         0.382426 Hz
AQ            1.3074932 sec
RG                 2048
DW               19.950 usec
DE                 6.50 usec
TE                300.4 K
D1           2.00000000 sec
d11          0.03000000 sec
DELTA        1.89999998 sec
MCREST       0.00000000 sec
MCWRK        0.01500000 sec

======== CHANNEL f1 ========
NUC1                13C
P1                 8.90 usec
PL1                5.00 dB
SFO1        100.6243390 MHz

======== CHANNEL f2 ========
CPDPRG2         waltz16
NUC2                 1H
PCPD2            100.00 usec
PL2                0.00 dB
PL12              19.10 dB
PL13              22.10 dB
SFO2        400.1319000 MHz

F2 - Processing parameters
SI                32768
SF          100.6127729 MHz
WDW                  EM
SSB                   0
LB                 1.00 Hz
GB                    0
PC                 1.40

1D NMR plot parameters
CX                20.00 cm
CY                12.50 cm
F1P             220.000 ppm
F1             22134.81 Hz
F2P             -10.000 ppm
F2             -1006.13 Hz
PPMCM          11.50000 ppm/cm
HZCM         1157.04688 Hz/cm
```

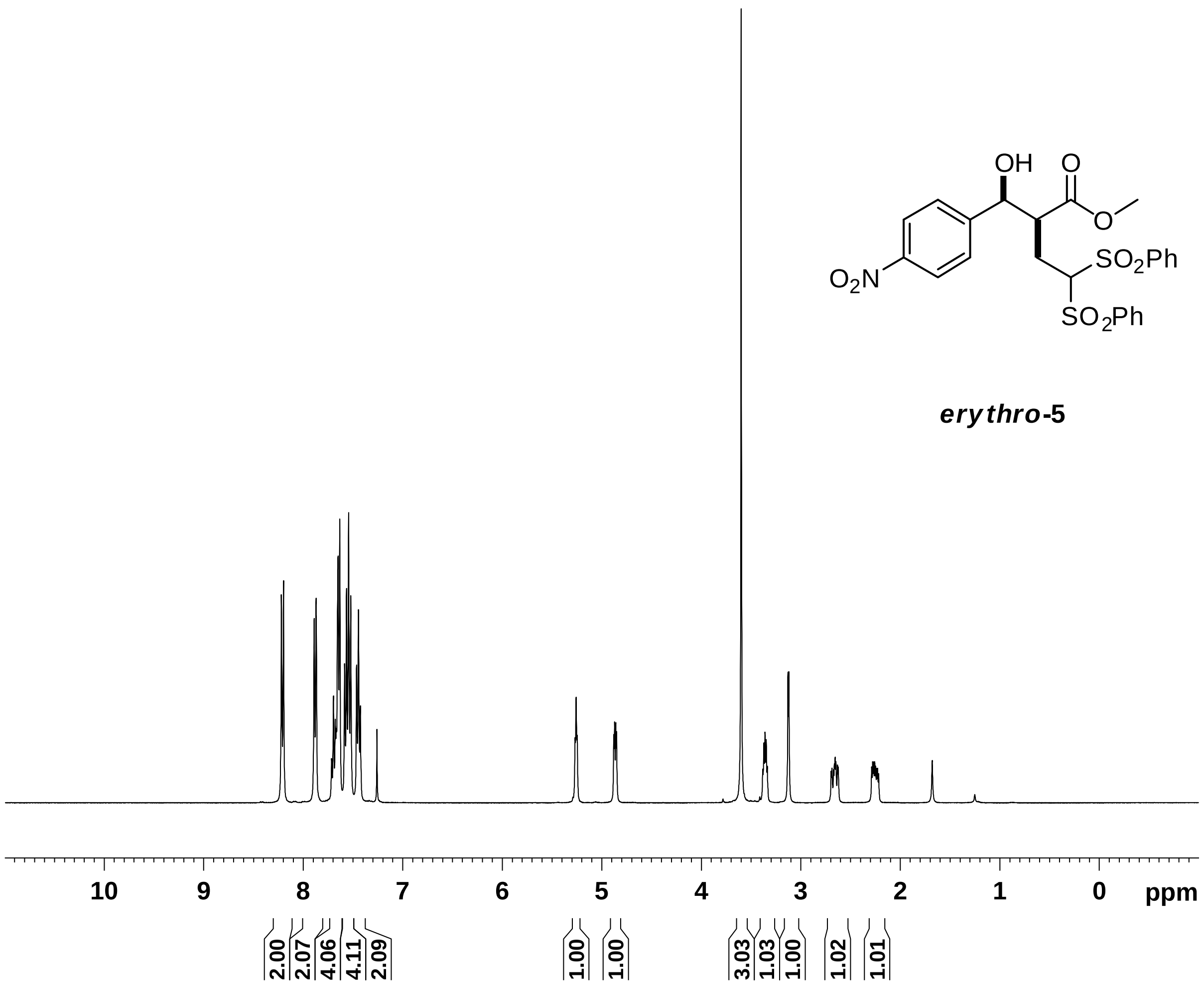


```
Current Data Parameters
NAME              ncy151
EXPNO                 15
PROCNO                 1

F2 - Acquisition Parameters
Date_           20100505
Time               20.40
INSTRUM            spect
PROBHD   5 mm BBO BB-1H
PULPROG             zg30
TD                 16384
SOLVENT            CDCl3
NS                    16
DS                     0
SWH             5995.204 Hz
FIDRES          0.365918 Hz
AQ             1.3664756 sec
RG                   128
DW                83.400 usec
DE                  8.00 usec
TE                 298.4 K
D1            2.00000000 sec
MCREST        0.00000000 sec
MCWRK         0.01500000 sec

======== CHANNEL f1 ========
NUC1                  1H
P1                 10.00 usec
PL1                 0.00 dB
SFO1         400.1326008 MHz

F2 - Processing parameters
SI                 16384
SF           400.1300095 MHz
WDW                   EM
SSB                    0
LB                  0.10 Hz
GB                     0
PC                  1.00
```

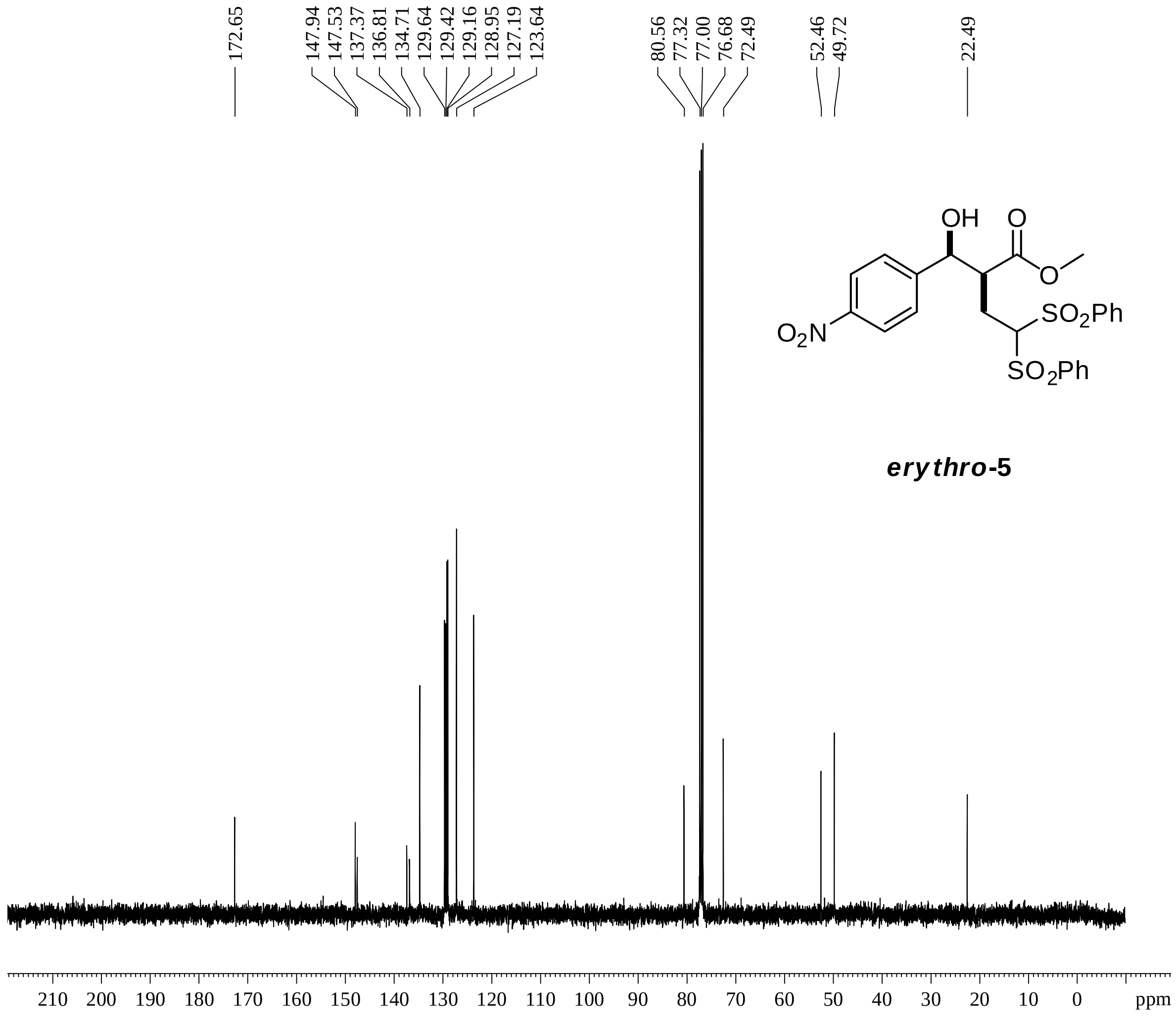

***erythro*-5**

```
Current Data Parameters
NAME             ncy151
EXPNO                17
PROCNO                1

F2 - Acquisition Parameters
Date_          20100514
Time              14.52
INSTRUM           spect
PROBHD   5 mm BBO BB-1H
PULPROG          zgpg30
TD                65536
SOLVENT           CDCl3
NS                  222
DS                    0
SWH           25125.629 Hz
FIDRES         0.383387 Hz
AQ            1.3042164 sec
RG                 4096
DW               19.900 usec
DE                 8.00 usec
TE                299.0 K
D1           2.00000000 sec
d11          0.03000000 sec
DELTA        1.89999998 sec
MCREST       0.00000000 sec
MCWRK        0.01500000 sec

======== CHANNEL f1 ========
NUC1                13C
P1                 8.90 usec
PL1                5.00 dB
SFO1        100.6243390 MHz

======== CHANNEL f2 ========
CPDPRG2         waltz16
NUC2                 1H
PCPD2             90.00 usec
PL2                0.00 dB
PL12              19.10 dB
PL13              22.10 dB
SFO2        400.1319000 MHz

F2 - Processing parameters
SI                32768
SF          100.6127756 MHz
WDW                  EM
SSB                   0
LB                 1.00 Hz
GB                    0
PC                 1.40

1D NMR plot parameters
CX                20.00 cm
CY                12.01 cm
F1P             220.000 ppm
F1             22134.81 Hz
```

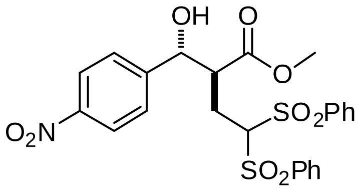

***threo*-5**

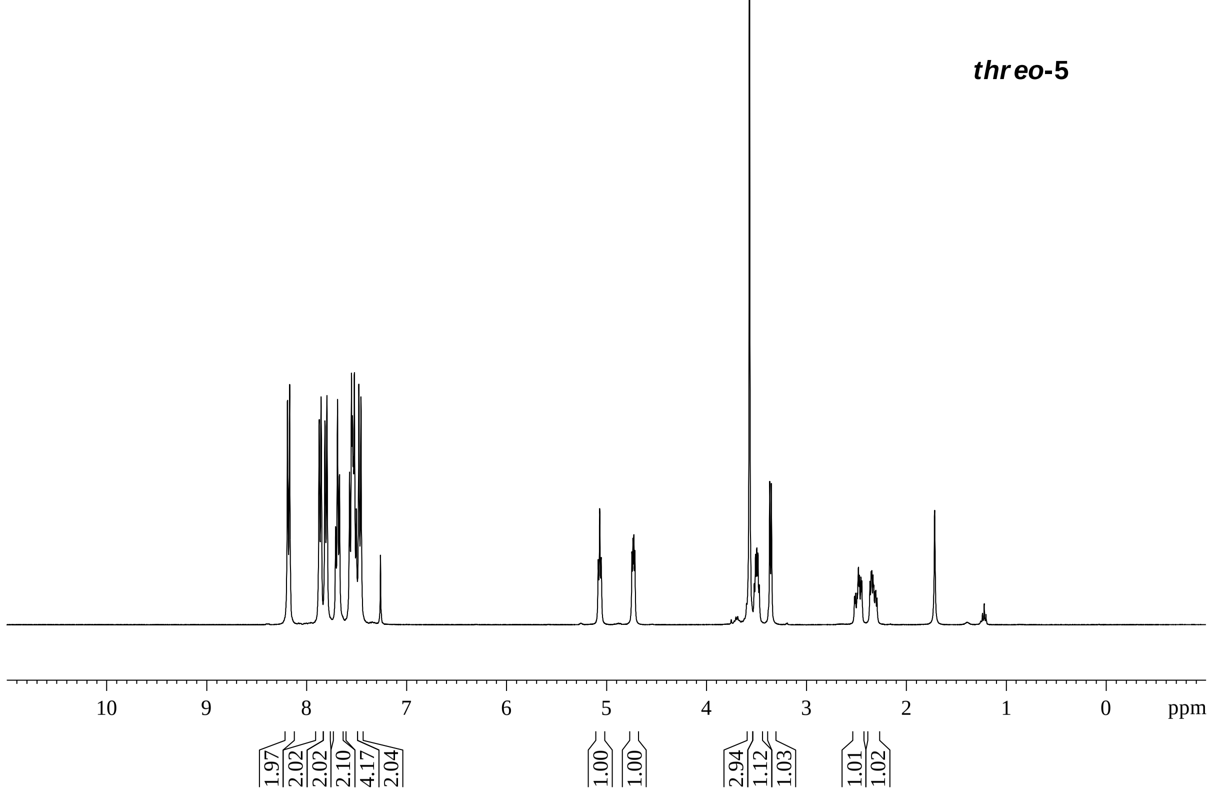


```
Current Data Parameters
NAME              ncy151
EXPNO                 13
PROCNO                 1

F2 - Acquisition Parameters
Date_           20100505
Time               12.23
INSTRUM            spect
PROBHD   5 mm BBO BB-1H
PULPROG             zg30
TD                 16384
SOLVENT            CDCl3
NS                    16
DS                     0
SWH             5995.204 Hz
FIDRES          0.365918 Hz
AQ             1.3664756 sec
RG                   128
DW                83.400 usec
DE                  8.00 usec
TE                 298.7 K
D1            2.00000000 sec
MCREST        0.00000000 sec
MCWRK         0.01500000 sec

======== CHANNEL f1 ========
NUC1                  1H
P1                 10.00 usec
PL1                 0.00 dB
SFO1         400.1326008 MHz

F2 - Processing parameters
SI                 16384
SF           400.1300091 MHz
WDW                   EM
SSB                    0
LB                  0.10 Hz
GB                     0
PC                  1.00
```

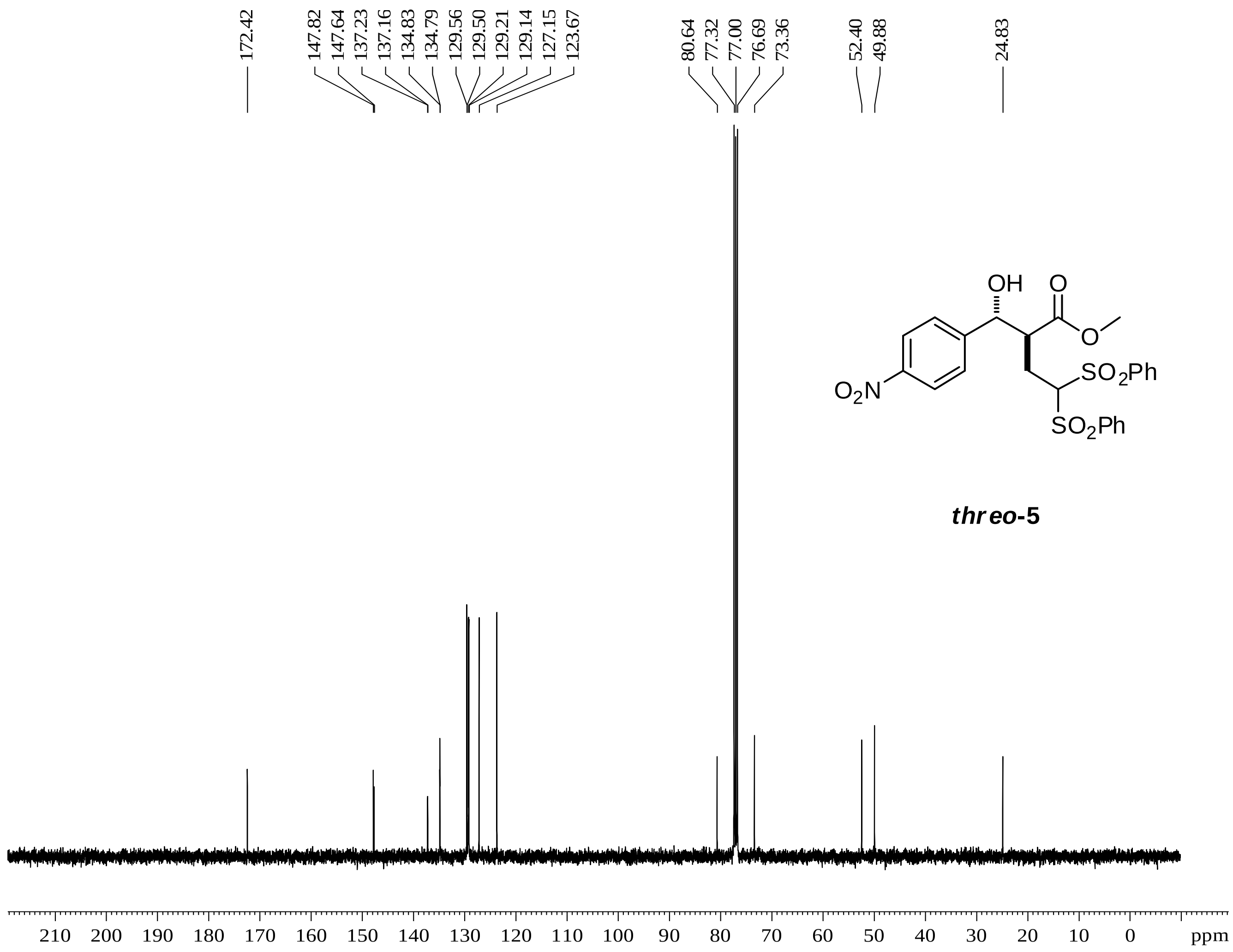

***threo*-5**

```
Current Data Parameters
NAME              ncy151
EXPNO                 14
PROCNO                 1

F2 - Acquisition Parameters
Date_           20100505
Time               12.26
INSTRUM            spect
PROBHD   5 mm BBO BB-1H
PULPROG          zgpg30
TD                 65536
SOLVENT            CDCl3
NS                   231
DS                     0
SWH            25125.629 Hz
FIDRES          0.383387 Hz
AQ             1.3042164 sec
RG                  4096
DW                19.900 usec
DE                  8.00 usec
TE                 299.0 K
D1            2.00000000 sec
d11           0.03000000 sec
DELTA         1.89999998 sec
MCREST        0.00000000 sec
MCWRK         0.01500000 sec

======== CHANNEL f1 ========
NUC1                 13C
P1                  8.90 usec
PL1                 5.00 dB
SFO1         100.6243390 MHz

======== CHANNEL f2 ========
CPDPRG2          waltz16
NUC2                  1H
PCPD2              90.00 usec
PL2                 0.00 dB
PL12               19.10 dB
PL13               22.10 dB
SFO2         400.1319000 MHz

F2 - Processing parameters
SI                 32768
SF           100.6127740 MHz
WDW                   EM
SSB                    0
LB                  1.00 Hz
GB                     0
PC                  1.40

1D NMR plot parameters
CX                 20.00 cm
CY                 11.99 cm
F1P              220.000 ppm
F1              22134.81 Hz
```

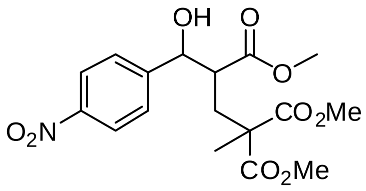

6-d1

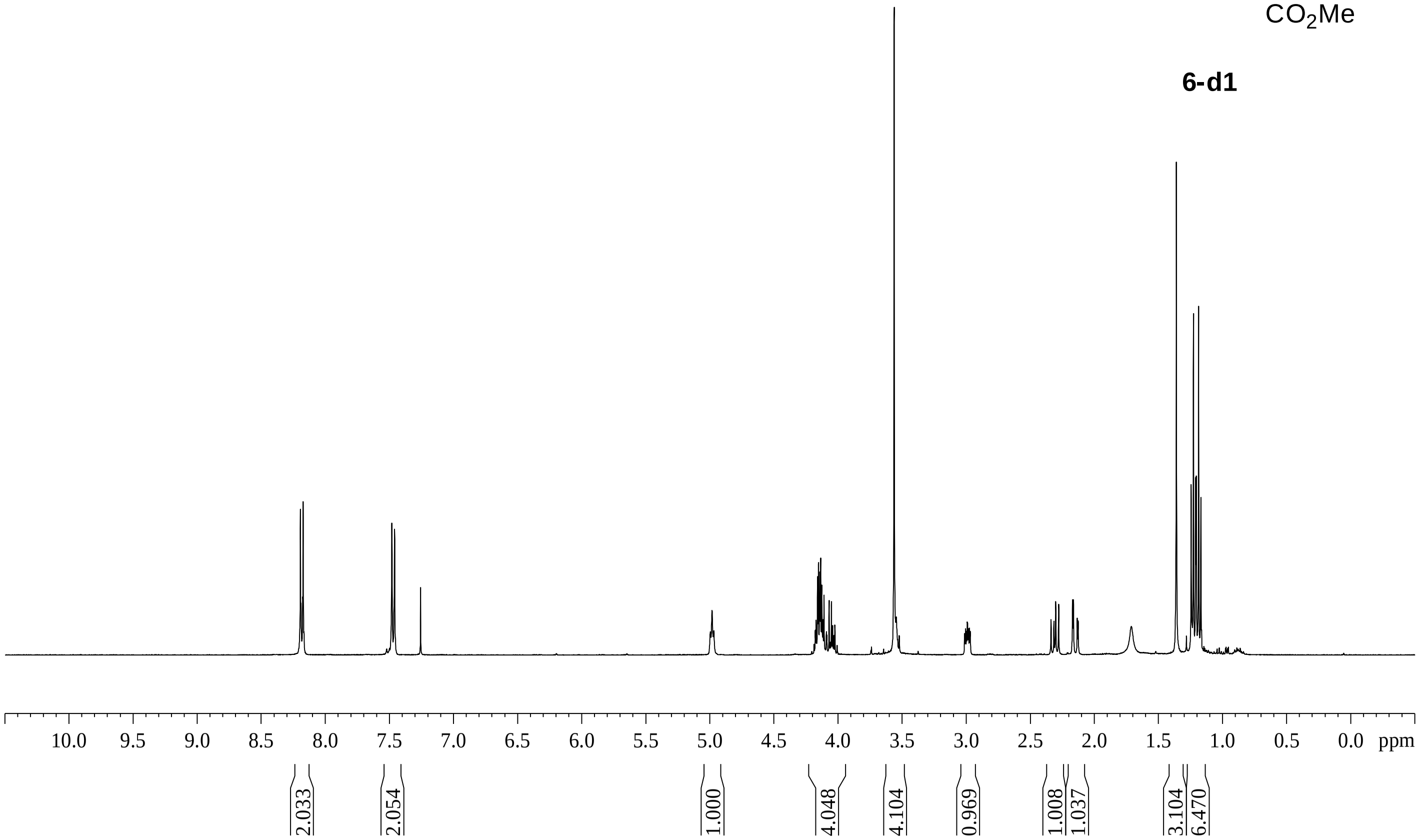


```
Current Data Parameters
NAME              ED0356
EXPNO                 12
PROCNO                 1

F2 - Acquisition Parameters
Date_           20100413
Time                7.14
INSTRUM            spect
PROBHD   5 mm BBO BB-1H
PULPROG             zg30
TD                 16384
SOLVENT            CDCl3
NS                     8
DS                     0
SWH             5995.204 Hz
FIDRES          0.365918 Hz
AQ             1.3664756 sec
RG                 161.3
DW                83.400 usec
DE                  8.00 usec
TE                 673.2 K
D1            1.50000000 sec
MCREST        0.00000000 sec
MCWRK         0.01500000 sec

======== CHANNEL f1 ========
NUC1                  1H
P1                  9.70 usec
PL1                 3.00 dB
SFO1         400.1326008 MHz

F2 - Processing parameters
SI                 16384
SF           400.1300095 MHz
WDW                   EM
SSB                    0
LB                  0.10 Hz
GB                     0
PC                  1.00
```

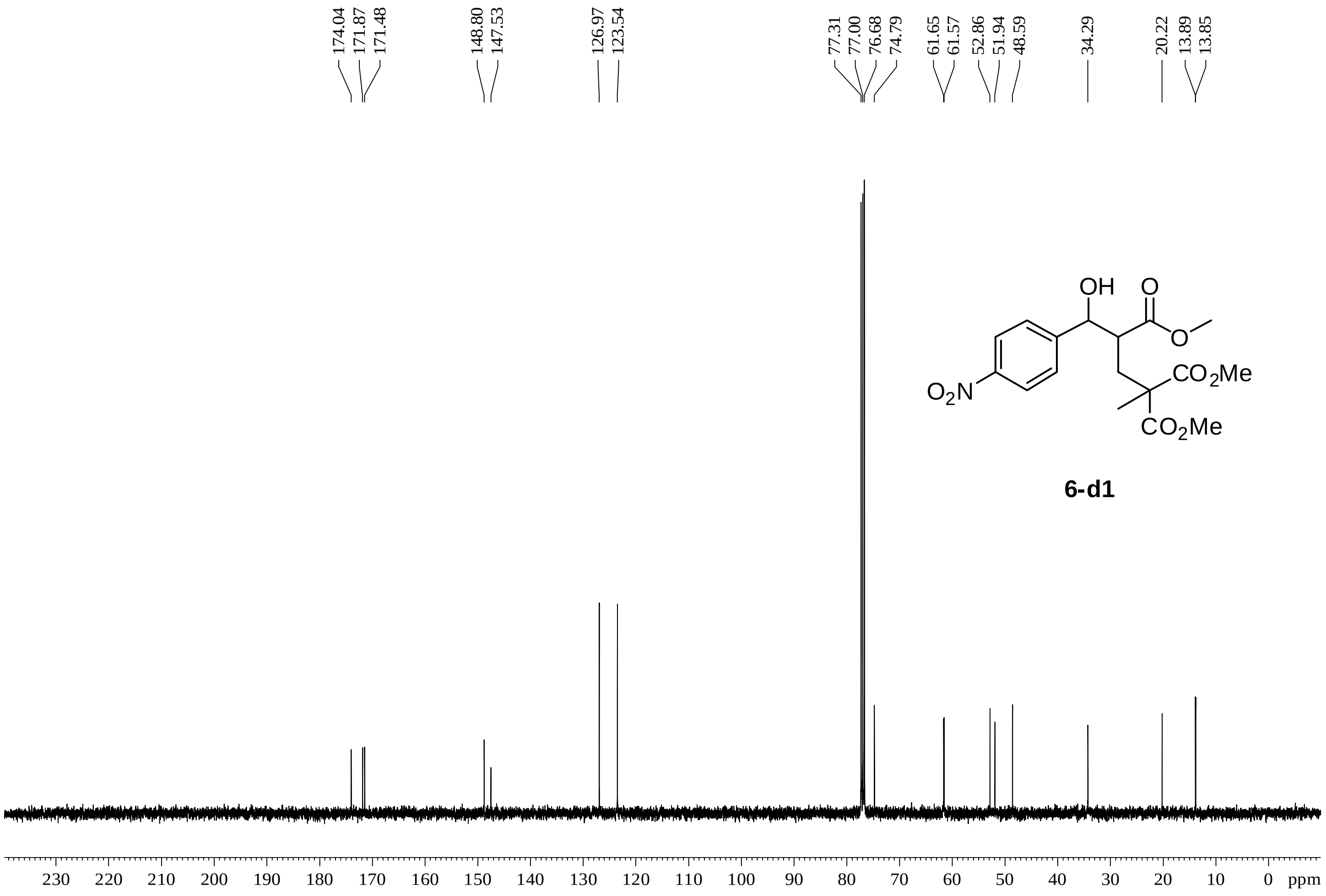


```
Current Data Parameters
NAME              ED0356
EXPNO                 13
PROCNO                 1

F2 - Acquisition Parameters
Date_           20100413
Time                7.15
INSTRUM            spect
PROBHD   5 mm BBO BB-1H
PULPROG           zgpg30
TD                 65536
SOLVENT            CDCl3
NS                   217
DS                     0
SWH            25125.629 Hz
FIDRES          0.383387 Hz
AQ             1.3042164 sec
RG                  4096
DW                19.900 usec
DE                  8.00 usec
TE                 673.2 K
D1            2.00000000 sec
d11           0.03000000 sec
DELTA         1.89999998 sec
MCREST        0.00000000 sec
MCWRK         0.01500000 sec

======== CHANNEL f1 ========
NUC1                 13C
P1                  9.60 usec
PL1                 7.00 dB
SFO1        100.6243390 MHz

======== CHANNEL f2 ========
CPDPRG2          waltz16
NUC2                  1H
PCPD2              90.00 usec
PL2                 3.00 dB
PL12               22.20 dB
PL13               25.20 dB
SFO2        400.1319000 MHz

F2 - Processing parameters
SI                 32768
SF          100.6127725 MHz
WDW                   EM
SSB                    0
LB                  1.00 Hz
GB                     0
PC                  1.40
```
